# 2016 SOSORT guidelines: orthopaedic and rehabilitation treatment of idiopathic scoliosis during growth

**DOI:** 10.1186/s13013-017-0145-8

**Published:** 2018-01-10

**Authors:** Stefano Negrini, Sabrina Donzelli, Angelo Gabriele Aulisa, Dariusz Czaprowski, Sanja Schreiber, Jean Claude de Mauroy, Helmut Diers, Theodoros B. Grivas, Patrick Knott, Tomasz Kotwicki, Andrea Lebel, Cindy Marti, Toru Maruyama, Joe O’Brien, Nigel Price, Eric Parent, Manuel Rigo, Michele Romano, Luke Stikeleather, James Wynne, Fabio Zaina

**Affiliations:** 1 Clinical and Experimental Sciences Department, University of Brescia Viale Europa 11, Brescia, Italy; 2 IRCCS Fondazione Don Gnocchi, Milan, Italy; 3 ISICO (Italian Scientific Spine Institute), Via R. Bellarmino 13/1, 20141 Milan, Italy; 4 U.O.C. of Orthopedics and Traumatology, Children’s Hospital Bambino Gesù, Institute of Scientific Research, 00165 Rome, Italy; 5 Center of Body Posture, Olsztyn, Poland; 6 Department of Physiotherapy, Józef Rusiecki University College, Olsztyn, Poland; 7 Faculty of Rehabilitation Medicine, University of Alberta, Edmonton, Canada; 8 Alberta Health Services, Department of Surgery, Edmonton, Canada; 9 Orthopedic Medicine - Clinique du Parc, Lyon, France; 10 Department of Orthopedics and Trauma Surgery, University Medical Center, Mainz, Germany; 11 Department of Orthopaedics and Traumatology, “Tzaneio” General Hospital of Piraeus, Piraeus, Greece; 12 Rosalind Franklin University of Medicine and Science, North Chicago, IL USA; 13 Department of Spine Disorders and Pediatric Orthopedics, University of Medical Sciences, Poznan, Poland; 14 Scoliosis Physiotherapy & Posture Centre, 231 McLeod Street, Ottawa, Ontario K2P0Z8 Canada; 15 Schroth-Barcelona Institute, LLC, Spinal Dynamics of Wisconsin, SC., Barcelona, Spain; 16 Saitama Prefectural Rehabilitation Center, Saitama, Japan; 17 National Scoliosis Foundation, Stoughton, MA USA; 18 Section of Spine Surgery, Children’s Mercy Hospitals and Clinics, UMKC Orthopedics, Kansas City, MO USA; 19 Department of Physical Therapy, 2-50 Corbett Hall, Edmonton, AB T6G 2G4 Canada; 20 National Scoliosis Center, 3023 Hamaker Court, Suite LL-50, Fairfax, VA 22124 USA; 21 Boston Orthotics & Prosthetics, Boston, MA USA; 22 Salvá SLP (E. Salvá Institute), Vía Augusta 185, 08021 Barcelona, Spain

**Keywords:** Idiopathic scoliosis, Treatment, Guidelines

## Abstract

**Background:**

The International Scientific Society on Scoliosis Orthopaedic and Rehabilitation Treatment (SOSORT) produced its first guidelines in 2005 and renewed them in 2011. Recently published high-quality clinical trials on the effect of conservative treatment approaches (braces and exercises) for idiopathic scoliosis prompted us to update the last guidelines’ version. The objective was to align the guidelines with the new scientific evidence to assure faster knowledge transfer into clinical practice of conservative treatment for idiopathic scoliosis (CTIS).

**Methods:**

Physicians, researchers and allied health practitioners working in the area of CTIS were involved in the development of the 2016 guidelines. Multiple literature reviews reviewing the evidence on CTIS (assessment, bracing, physiotherapy, physiotherapeutic scoliosis-specific exercises (PSSE) and other CTIS) were conducted. Documents, recommendations and practical approach flow charts were developed using a Delphi procedure. The process was completed with the Consensus Session held during the first combined SOSORT/IRSSD Meeting held in Banff, Canada, in May 2016.

**Results:**

The contents of the new 2016 guidelines include the following: background on idiopathic scoliosis, description of CTIS approaches for various populations with flow-charts for clinical practice, as well as literature reviews and recommendations on assessment, bracing, PSSE and other CTIS. The present guidelines include a total of 68 recommendations divided into following topics: bracing (*n* = 25), PSSE to prevent scoliosis progression during growth (*n* = 12), PSSE during brace treatment and surgical therapy (*n* = 6), other conservative treatments (*n* = 2), respiratory function and exercises (*n* = 3), general sport activities (*n* = 6); and assessment (*n* = 14). According to the agreed strength and level of evidence rating scale, there were 2 recommendations on bracing and 1 recommendation on PSSE that reached level of recommendation “I” and level of evidence “II”. Three recommendations reached strength of recommendation A based on the level of evidence I (2 for bracing and one for assessment); 39 recommendations reached strength of recommendation B (20 for bracing, 13 for PSSE, and 6 for assessment).The number of paper for each level of evidence for each treatment is shown in Table 8.

**Conclusion:**

The 2016 SOSORT guidelines were developed based on the current evidence on CTIS. Over the last 5 years, high-quality evidence has started to emerge, particularly in the areas of efficacy of bracing (one large multicentre trial) and PSSE (three single-centre randomized controlled trials). Several grade A recommendations were presented. Despite the growing high-quality evidence, the heterogeneity of the study protocols limits generalizability of the recommendations. There is a need for standardization of research methods of conservative treatment effectiveness, as recognized by SOSORT and the Scoliosis Research Society (SRS) non-operative management Committee.

**Electronic supplementary material:**

The online version of this article (10.1186/s13013-017-0145-8) contains supplementary material, which is available to authorized users.

## Premise

### Mandate

This is the third edition of the guidelines promoted by the international Scientific Society on Scoliosis Orthopaedic and Rehabilitation Treatment (SOSORT). The first guidelines were produced in Milan in 2005 and published in 2006 in Scoliosis and Spinal Deformities Journal [1, 2], followed by the guidelines update published in 2012 [3]. In the light of emerging evidence in the past 5 years on conservative treatment for scoliosis, we revised them again. The objective of the SOSORT Committee was to align the guidelines with the new scientific evidence and offer updated recommendations to assure faster knowledge transfer into clinical practice of conservative treatment of idiopathic scoliosis (CTIS). In the attempt to update each section in depth, it was decided that the next updates of the guidelines will be divided into different section, the next update will be on 2019 and will regard the chapter of General informations on idiopathic scoliosis, then 2 years later (2021) brace chapter will be published and updating the current knowledge. The exercises chapter will follow 2 years later in 2023, and evaluations will be updated in 2025.

### Committee

The Committee was open to all SOSORT Members who decided to adhere to the project, and it is now composed by a group of SOSORT member lead by Stefano Negrini, member of the SOSORT Advisory Board and Past President of the SOSORT, helped by Angelo Gabriele Aulisa, member of the SOSORT Scientific Board.

### Content

The contents of the document of the 2016 SOSORT guidelines on “Orthopaedic and Rehabilitation Treatment of Idiopathic Scoliosis During Growth” include the following:

1. Methodology
2. Background on idiopathic scoliosis
3. Approach to conservative treatment of idiopathic scoliosis in different patients, with practical flow-charts
4. Literature review and recommendations on assessment, bracing, physiotherapy, physiotherapeutic scoliosis-specific exercises (PSSE) and other conservative treatments

A detailed description of the methods is presented in Additional file 1.

### Scope, purpose, and applications

The aim of these guidelines was to present the evidence-based updated review and clinical recommendations on the conservative treatment for scoliosis during growth. The multiple grey areas, important for everyday clinical practice, for which was not possible to provide evidence-based recommendations, were discussed in multiple structured surveys using Delphi method (Additional file 1).

The guidelines were meant to apply to all growing patients with idiopathic scoliosis. The main clinical questions that they assessed include the following:

- How should a patient be assessed?
- Which conservative treatment should be provided, and how?
- How and when should bracing be applied?
- How and when should exercises be used?

### Development of the guidelines

Various types of professionals engaged in the conservative treatment of scoliosis have been involved: specialty physicians (orthopaedics, physical and rehabilitation medicine, psychiatry) and allied health professionals (orthotists, physiotherapists, chiropractors).

These guidelines were developed by the Society on Scoliosis Orthopaedic and Rehabilitation Treatment (SOSORT), whose focus is the conservative treatment approaches for scoliosis. The other two international scientific societies dedicated to research into, and treatment for spinal deformities, primarily focus on the surgical treatment (Scoliosis Research Society) or on general research (International Research Society on Spinal Deformities). The SRS and IRSSD did not participate in the development of the guidelines, although several members of these Societies are also members of the SOSORT. Moreover, the final Consensus was held during a joint SOSORT/IRSSD meeting.

Patients have been involved in the development of the guidelines, through the US National Scoliosis Foundation, representing 25,000 patients with scoliosis.

### Methods

Methods are outlined in detail in the Appendix (Additional file 1). For the treatment sections, we updated the previously performed reviews of the literature looking for all papers from December 2010 to December 2015. The search strategies, the selection criteria, and the number of retrieved papers are listed in the individual sections. We also hand-searched the abstracts of all SOSORT Meetings, from 2010 to 2015; we checked the references of the included articles and consulted personal files and knowledge of all the authors. To update these guidelines, we revised the previous ones [1–4]. The final documents, recommendations, and practical approach flow charts have been developed according to a Delphi procedure listed in the Appendix (Additional file 1). After a review process, the final Consensus Session was held during the 2016 Banff SOSORT and IRSSD Joint Meeting. A classical Level of Evidence (LoE) table has been adopted (Table 1). As in the Italian Guidelines and the SOSORT 2011 guidelines [2, 3], levels V and VI have been added according to the Consensus session held during the SOSORT Meeting. A Strength of Recommendation Taxonomy (SoRT) has also been used (Table 2) that states the strength that each Recommendation should have in the clinical world, balancing all typical factors involved in this decision (patients, professionals, social). The SoRT scale is meant to accompany and complement the Strength of Evidence scale and it consists of grades A, B and C.

**Table 1:** Strength of evidence grading used in these guidelines. Questions on effectiveness (treatment results) and diagnosis (assessment) have been considered

Strength of evidence	Question	Meaning
I	Effectiveness	Multiple Randomized Controlled Trials or Systematic Reviews of such studies
	Diagnosis	Multiple Randomized Controlled Trials, or Cross-sectional Studies with verification by reference (gold) standard, or Systematic Reviews of such studies
II	Effectiveness	One Randomized Controlled Trial
	Diagnosis	One Randomized Controlled Trial, or one Cross-sectional Study with verification by reference (gold) standard
III	Effectiveness	Multiple Controlled nonrandomized Studies or Systematic Reviews of such studies
	Diagnosis	Multiple Cross-sectional Studies with incomplete & unbalanced verification with reference (gold) standard
IV	Effectiveness	Other studies
	Diagnosis	
V	Effectiveness	SOSORT consensus with more than 90% of agreement
	Diagnosis	
VI	Effectiveness	SOSORT consensus with 70 to 89% of agreement
	Diagnosis

**Table 2:** Strength of recommendation grading used in these guidelines

Strength of recommendation	Meaning
A	It must be applied widely and to all patients with this specific need
B	It is important, but does not have to be applied to all patients with this specific need
C	Less important, it can be applied on a voluntary basis
D	Very low importance

### Target users of the guidelines

These guidelines are targeted to the professionals involved in the Conservative Treatment of Scoliosis, and their patients.

### Updates

We project that these 2016 guidelines will be updated by SOSORT in 3 to 5 years. If important changes in practice occur before that, an earlier update may be warranted.

### Applicability

These guidelines will be published in the Open Access Journal “Scoliosis and Spinal Disorders” (http://www.scoliosisjournal.com). Open Access will ensure the visibility and accessibility to the worldwide community of stakeholders, including researchers and practitioners interested in conservative treatment of scoliosis, as well as patients. The Consensus process, involving professionals from all over the world, should provide an objective document that a wide variety of interested organizations and third party payers may review to gain insight into the treatment modalities. In the meantime, single national adaptations should eventually be considered. The guidelines itself should serve as basis for these national documents.

Translations in different languages have been planned. These translations will be published on the Official SOSORT website: http://www.sosort.mobi.

## General information on idiopathic scoliosis

### Definitions

Scoliosis is a general term comprising a heterogeneous group of conditions consisting in changes in the shape and position of the spine, thorax and trunk.

Hippocrates spoke of “spina luxate”, gathering all the vertebral deviations. It is Galen who defined the first “scoliosis” (sKolios, which means crooked or curved) [5], by meaning an abnormal lateral spinal curvature. “Structural scoliosis”, or just scoliosis, must be differentiated from “functional scoliosis” that is a spinal curvature secondary to known extra spinal causes (e.g. shortening of a lower limb or paraspinal muscle tone asymmetry). It is usually partially reduced or completely subsides after the underlying cause is eliminated (e.g. in a recumbent position). Functional scoliosis is not the subject of this paper. The term idiopathic scoliosis was introduced by Kleinberg [6], and it is applied to all patients in which it is not possible to find a specific disease causing the deformity; in fact, it occurs in apparently healthy children and can progress in relation to multiple factors during any rapid period of growth. By definition, idiopathic scoliosis is of unknown origin and is probably due to several causes. Etiopathogenetically, the spinal deformity caused by idiopathic scoliosis may be defined as a sign of a syndrome with a multifactorial etiology [7–9]. Nearly always, scoliosis manifests as a solitary deformity, but further investigation may reveal other significant subclinical signs [10, 11]. Idiopathic Scoliosis has been described as a torsional deformity of the spine, with several torsional regions joined by a junctional zone, every region including a variable number of morphologically lordotic vertebrae translated and rotated to the same side [12]. Notwithstanding, although the morphological lordotization (flat back), related to a secondary relative anterior spinal overgrowth is an almost constant when looking at the middle sagittal plane of the central scoliotic region (apex), the geometry of the spine is highly variable when observing the spine on a latero-lateral radiograph (middle sagittal plane of the patient), Trunk deformity and back asymmetry correlates with the spinal deformity, but there can be significant discrepancies in some cases [13].

The curvature in the frontal plane (AP radiograph in upright position) is limited by an “upper end vertebra” and a “lower end vertebra”, taken both as a reference level to measure the Cobb angle. The Scoliosis Research Society (SRS) suggests that the diagnosis is confirmed when the Cobb angle is 10° or higher and axial rotation can be recognized. Maximum axial rotation is measured at the apical vertebra. However, structural scoliosis can be seen with a Cobb angle under 10° [7], with a potential for progression. Progression is more common in girls during the growth spurt at puberty, and then, it is called progressive idiopathic scoliosis. When untreated, it may lead to severe trunk deformities, which limit the capacity and functional biomechanics of the chest, exercise capacity, general fitness and ability to work, all factors related with impairment on quality of life.

### Epidemiology

In approximately 20% of cases, scoliosis is secondary to another pathological process. The remaining 80% are cases of idiopathic scoliosis. Adolescent idiopathic scoliosis (AIS) with a Cobb angle above 10° occurs in the general population in a wide range of prevalence from 0.93 to 12% [8, 9, 14–29]: 2 to 3% is the value the most often found in the literature, and it has been suggested that the incidence changes according to latitude [15, 30].

Approximately 10% of these diagnosed cases require conservative treatment and approximately 0.1–0.3% require operative correction of the deformity. Progression of AIS is much more frequently seen in females. When the Cobb angle is 10 to 20°, the ratio of affected girls to boys is similar (1.3:1), increasing to 5.4:1 for Cobb angles between 20° and 30°, and 7:1 for angle values above 30° [31, 32]. If the scoliosis angle at completion of growth exceeds a “critical threshold” (most authors assume it to be between 30° and 50° [33], there is a higher risk of health problems in adult life, decreased quality of life, cosmetic deformity and visible disability, pain and progressive functional limitations [32, 34].

### Etiology

The etiopathogenesis of scoliosis has not been elucidated. The causes of scoliosis are being sought in congenital or acquired disorders of vertebral structure. Patients with this type of deformity are usually noted to suffer from such co-existent abnormalities as asymmetrical structure of the brain stem, sensory and balance impairment, disorders of blood platelet and collagen function [4, 5]. The role of genetic factors in the development of spinal axial disorders is also emphasized and is confirmed by the tendency of scoliosis to run in families, with researchers suggesting a hereditary disorder of oestrogen receptor structure and function [35]. Numerous authors indicate that the causes of scoliosis are systemic disorders of, among others, mucopolysaccharide and lipoprotein synthesis [36, 37]. In the 1990s, a group of researchers under the guidance of Dubousset proposed that scoliosis develops as a result of melatonin synthesis disorder [38–42]. They produced spinal curvatures in chickens via pinealectomy and later ameliorated the melatonin deficiency to find decreased incidence of scoliosis in the animals. Machida reported reduced serum melatonin levels in girls with rapidly progressive idiopathic scoliosis. His finding has been questioned by other authors, who found no differences between melatonin levels in scoliotic girls and those in a healthy control group [37–41]. Currently, melatonin is attributed only a limited role in scoliosis pathogenesis [43]. The possible role of melatonin in scoliosis etiology is also discussed in connection to age at menarche in different geographic latitudes [15]. According to more recent studies, calmodulin may disturb melatonin levels. Kindsfater et al. [44] assessed calmodulin levels in order to determine the risk of curve progression. Based on this hypothesis, melatonin plays a secondary role in the spontaneous induction of scoliosis. It is a consequence of interaction with calmodulin, a protein that has receptors for calcium ions and is thus able to influence the contractility of skeletal muscles; it can also be found in blood platelets (its level in platelets was higher in patients with scoliotic progression rates of more than 10° over 12 months) [35]. Other authors have evaluated the possibility that gene variants of IL-6 and MMPs might be associated with scoliosis and suggest that MMP-3 and IL-6 promoter polymorphisms constitute important factors for the genetic predisposition to scoliosis [45]. More recently, an increased BNC2 expression has been implicated in the etiology of AIS [44]. In summary, the etiology of scoliosis has not been fully elucidated [46, 47]. Based on the variety of opinions on idiopathic scoliosis development, we can assume a multifactorial origin. The opinions presented above are supplementary rather than mutually exclusive. At the same time, they explain the complex determinants of and relationships between disorders of spinal development in children and adolescents.

### Natural history

Idiopathic scoliosis (IS) may develop at any time during childhood and adolescence. It most commonly appears in periods of growth spurt-the first is in the first months of life, generally between 6 and 24 months, the between the age of 5 and 8 years, there is a height peak growth and at puberty the most important and rapid growth spurt, generally at age 11 to 14 years of life [2, 3, 48]. The rate of development of spinal curvature changes the most rapidly at the beginning of puberty [14, 15].

According to the Tanner scale, which assesses tertiary sex maturation characteristics, this period corresponds to stage S2 and P2 in girls and T2 and P2 in boys [16]. The pubertal growth spurt begins with accelerated longitudinal growth of limbs, which causes a temporary disproportion of the body (long limbs and short trunk). Then, longitudinal growth is seen in the axial skeleton. It is the period of the most marked progression of IS. After approximately 2/3 of the period of pubescent growth spurt, girls experience menarche, which indicates that the peak of growth has been passed, with a gradual decrease in the risk of scoliosis progression. There is a much lower potential for progression of idiopathic scoliosis after the spinal growth is complete. In adulthood, IS may intensify as a result of progressive osseous deformities and collapsing of the spine. This phenomenon is reported especially in scoliosis that is more severe than 50°, while the risk of progression starts to increase as the curve grows above 30° [17, 21, 22, 34]; less severe idiopathic scoliosis curves often remain stable. Nevertheless, the natural history of adult scoliosis is not well known to date, and it is still possible the progression can have some peak periods [49]. Typically, in adult scoliosis, the evolution of AIS with delayed risk of rotatory dislocation is differentiated from a “de novo” scoliosis rapidly changing in a few years to the rotatory dislocation [50, 51].

### Classifications

During the years, many different classifications of idiopathic scoliosis have been proposed, but not all of them are either relevant for conservative care or currently used beyond research purposes. Recent developments in 3D reconstructions of all spine deformities using standard or digital radiography allow to deepen the analysis of the scoliosis deformity in all space planes. In the text, we present the classifications endorsed by SOSORT Consensus (Table 3).

**Table 3:** Classifications of idiopathic scoliosis

Chronological (SoE: V)		Angular (SoE: VI)		Topographic (SoE: V)		
Age at diagnosis (years.months)		Cobb degrees			Apex	
					from	to
Infantile	0–2.	Low	Up to 20	Cervical	–	Disc C6–7
Juvenile	3–9.	Moderate	21–35	Cervico-thoracic	C7	T1
Adolescent	10–17.	Moderate to severe	36–40	Thoracic	Disc T1–2	Disc T11–12
Adult	18+	Severe	41–50	Thoraco-lumbar	T12	L1
		Severe to very severe	51–55	Lumbar		Disc L1–2
		Very severe	56 or more			

#### Chronological

James [52, 53] proposed that scoliosis should be classified based on the age of the child at which the deformity was diagnosed (Table 3). This classification is important since the longer the period between diagnosis of scoliosis and completion of growth by the developing child, the greater the risk of developing a more severe and complicated deformity. Today, the general term “Early onset scoliosis” is sometimes used to classify together Infantile and Juvenile scoliosis, but we prefer the James classification, due to the fact that infantile scoliosis has a different prognosis. In fact, there are congenital postural scoliosis curves diagnosed in newborns, as a component of a syndrome usually resulting from intrauterine compression caused by malposition of the fetus during pregnancy, but they represent exceptional conditions. Such curvatures are not three-plane deformities and usually undergo spontaneous remission. As the range of hip motion is often asymmetrical and the child prefers to rest their head on one side only, exercises and correction of body position are usually employed. Examination usually reveals gradual remission of the curvature in these infants, and such scoliosis curves may thus be categorized as regressive [54].

#### Angular

The angle of scoliosis measured on the standing frontal radiograph according to the Cobb method is one of the decisive factors in managing idiopathic scoliosis, and it is directly correlated to all treatment decisions. Many different classifications have been proposed based on these angular measurements, but no one system today has widespread validity. Nevertheless, there is an agreement on some thresholds [32, 34, 55–57]:

Under 10° of scoliosis, the diagnosis of scoliosis should not be made. The inter-reliability of the Cobb angle is well known, and the potential limitation of this criterion are clear. On the other hand, a clear and simple criterion is needed for a generally accepted and a simple agreed definition of structural scoliosis.

- Over 30° of scoliosis, the risk of progression in adulthood increases, as well as the risk of health problems and reduction of quality of life.
- Over 50°, there is a consensus that it is almost certain that scoliosis is going to progress in adulthood and cause health problems and reduction of quality of life.

From these thresholds, and taking into account that the recognized measurement error in measuring Cobb angles is 5° [58–63], very important decisions are made. When measured manually on the radiograph, the most commonly cited measurement error of Cobb angle is indeed 5° [58–63]. However, new computer-assisted measurement methods have lesser measurement errors, ranging from 1.22° to 3.6° [64]. When making clinical decisions, measurement error thresholds of a corresponding method used should be taken into account.

#### Topographic

Most commonly used classifications of idiopathic scoliosis are based on the anatomical site of the spinal deformity in the frontal plane. A classification developed by Ponseti [65] (based on Schulthess work [66]) distinguishes four major types of scoliosis: thoracic, lumbar, thoraco-lumbar and S-shaped. This classification is the oldest. It is reported in Table 3. It is used both in conservative treatment and in the pre-operative classification of scoliosis [67]. Two other classification systems of idiopathic scoliosis based on the anatomical site of spinal deformity have been proposed and used in preoperative planning [68–73]. The most widely used for operative treatment is Lenke classification [69]. This classification however uses some objective criteria that make it not applicable to be used for non-operative treatment. Mild scoliosis with indication for non-operative treatment, specific exercises or bracing, cannot be properly classified according to Lenke objective criteria. Patients under non-operative treatment rarely are prescribed a side bending radiograph, and even in that case, the criterion of “finding a residual coronal curve on side-bending radiographs of at least 25° in the proximal thoracic, main thoracic, thoracolumbar or lumbar regions, as a definition of a structural curve”, is not applicable to scoliosis in the range of 15° to 30°. Since these guidelines concern conservative treatment, the abovementioned classification is not discussed beyond here. Moreover, efforts were made to clinically evaluate the third dimension, mainly for surgical purposes; recently, several 3D classifications have been proposed [74–82], but the most useful one in clinical practice is yet to be defined [83].

#### Rigo classification

Many clinicians and brace developers base the treatment on some general and individualized criteria [84, 85], rather than to a classification able to guide brace fitting and construction as in the Rigo Cheneau brace and in the Spinecor System [73, 86]. The Rigo classification has been accepted (LoE VI) by these guidelines. They have been developed specifically to correlate with Rigo-Chenau brace design and treatment. The Rigo Cheneau classification was developed in order [72] to define specific principles of correction required for efficacious brace design and fabrication. The classification includes radiological as well as clinical criteria. The radiological criteria are utilized to differentiate five basic types of curvatures including (I) imbalanced thoracic (or three curves pattern), (II) true double (or four curve pattern), (III) balanced thoracic and false double (non 3 non 4), (IV) single lumbar and (V) single thoracolumbar. In addition to the radiological criteria, the Rigo Classification incorporates the curve pattern according to SRS terminology, the balance/imbalance at the transitional point, and L4-5 counter-tilting. This classification has been evaluated for intra-and inter-observer reliability: the intra-observer Kappa value was 0.87 (acceptance > 0.70); the inter-observer Kappa values fluctuated from 0.61 to 0.81 with an average of 0.71 (acceptance > 0.70) [72].

## Evidence-based clinical practice approach to idiopathic scoliosis during growth

### Goals of conservative treatment

#### General goals

A SOSORT 2005 Consensus paper, titled “Why do we treat adolescent idiopathic scoliosis? What do we want to obtain and to avoid for our patients” [34], can serve as reference for specific insights on this topic. In the present guidelines, the most general goals of treatment are presented in Table 4 [34].

**Table 4:** Goals of treatment according to the SOSORT consensus paper. Only the goals that reached 80% of agreement are listed here, starting from the most important

Esthetics	
Quality of life	
Disability	
Back pain	
Psychological well-being	
Progression in adulthood	
Breathing function	
Scoliosis Cobb degrees	
Need of further treatments in adulthood	

The goals of conservative treatment of idiopathic scoliosis may be divided into two groups: morphological and functional. The first aspect is related to aesthetics which was defined as the first goal of treatment by SOSORT experts. Both aspects are related to patients’ quality of life, psychological well-being and disability (defined as the second, third and fourth goals according to the SOSORT experts) [34]. For didactic reasons, the goals will be present here in a different order. The basic objectives of comprehensive conservative treatment of Idiopathic Scoliosis are as follows:

1. To stop curve progression at puberty (or possibly even reduce it)
2. To prevent or treat respiratory dysfunction
3. To prevent or treat spinal pain syndromes
4. To improve aesthetics via postural correction

##### To stop curve progression at puberty (or possibly even reduce it)

Recently, a multi-centre RCT demonstrated that bracing is effective at preventing progression to the surgical range (defined as ≥ 50°) [87], although on average the curves did not improve. Moreover, a long-term RCT found that PSSE improved Cobb angles at skeletal maturity in patients with AIS [88]. Current evidence suggests that conservative treatment for scoliosis is effective at stopping curve from progression, as well as improving the curves at skeletal maturity.

It is possible and usually sufficient to prevent further progression, even if recent research papers conducted according to the SRS criteria have shown that it is also possible to obtain some amount of curve correction [89–93].

##### To prevent or treat respiratory dysfunctions

The morphological aspect of the deformity is closely related to the effects on bodily function. Depending on its degree and location, the curvature may affect respiratory function. The most prominent changes within the respiratory system are produced by curvatures of the thoracic spine [94–97].

##### To prevent or treat spinal pain syndromes

Statistically significant differences in pain prevalence are already noted in people with scoliosis between 20 and 30 years of age. In a follow-up study of over 40 years’ duration, a three-fold higher prevalence of chronic pain-related complaints and over 20-fold higher incidence of severe and darting pain were observed in a group of people with untreated idiopathic scoliosis compared to a control group. The occurrence of pain-related complaints is probably multifactorial in origin [33, 50, 98–101].

In adult with spinal deformities, sagittal parameters influence pain the most as compared to the magnitude of scoliosis curve [102]. The assessment of regional and global alignment parameters in full-length standing postero-anterior and lateral, as well as pelvic parameters, is strongly recommended due to their relation with pain and disability [103]. In addition, pain is significantly correlated to three dimensional olysthesis, L3 and L4 endplate obliquity angles, loss of lumbar lordosis, and thoracolumbar kyphosis [102]**.**

The SRS-Schwab classification based on curve type and magnitude associated with specific index based on sagittal pelvic and spine parameters has been showed to be reliable and to correlate with quality of life in adults with spinal deformities [104]. This new classification suggests that there are specific parameters able to predict the risk of pain and disability, in adulthood. Currently, no studies have confirmed if it is possible to treat sagittal alterations during growth, or if the conservative treatment play a role in creating unbalanced spine in adults previously braced, nor if the same treatment is able to prevent future alteration of the sagittal profile of the spine and pelvis. Despite this knowledge gap, there is a general agreement among experts that the best possible treatment should take into account not only the correction of the spine in the coronal plane but also the maintenance or the restoration of the normal sagittal profile of the spine.

##### To improve the appearance via postural correction

Quality of life is significantly affected by aesthetic self-perception and appearance. Therefore, visual correction of scoliosis-related external trunk deformity is an important issue in conservative treatment. Therapeutic outcomes may be subjectively visually assessed using specifically designed questionnaire or objectively assessed using surface topography and photographic methods [13, 105–111].

#### Specific goals of conservative treatment during growth

Specific goals of conservative treatment for patients during growth should be set at baseline (X-ray before treatment). These goals should be considered as a dynamic continuum, which can be adapted during treatment according to the change in the patient clinical status (change in deformity, compliance with the treatment, proposed therapies, etc.). In this respect, we can define the following goals:

- Absolute goal: these are the minimum expected goals of conservative treatment. If not anything else, at least these goals should be reached.
- Primary goals: these are the “best possible” goals for patients starting treatment in each specific clinical situation.
- Secondary goals: these are the compromise goals that come when it becomes clear that it is not possible to reach the primary goals

According to this approach, SOSORT has reached a Consensus (Strength of Evidence VI— Strength of Recommendation C) shown in Table 5. This table has been organized with a minimum and a maximum of primary and secondary goals that can be reached for each clinical situation. The absolute goals for all patients in every clinical situation are to avoid fusion surgery. A first similar scheme had been proposed in 2007 [112]: these goals were applied in some studies [90, 91, 112] and proved to be useful. Accordingly, we propose these goals of treatment here to be applied in clinical studies of conservative treatment of idiopathic scoliosis.

**Table 5:** Specific aims of conservative treatment during growth (strength of evidence VI–strength of recommendation C) at least 70% of agreement (SoE VI)

Absolute aim of treatments		Percentage
Avoid surgery		90.70
Improve aesthetics		86.05
Improve quality of life		82.56
Degree of curve	Primary aim	Secondary aim
Low	Remain below 20°	Remain below 45°
Moderate	Remain below 30°	Remain below 45°
Severe	Remain below 45°	Postpone surgery

#### Evidence-based clinical practice approach

This section is constituted mainly by a Practical Approach Scheme (PAS) (Table 6) that has been prepared through the Consensus Procedure reported in (Additional file 1). The PAS constitutes an Evidence-Based Clinical Practice approach to idiopathic scoliosis. The Level of Evidence of PAS is VI, while the Strength of Recommendation is B.

**Table 6:** Practical approach scheme (PAS) for an evidence-based clinical practice approach to idiopathic scoliosis (strength of evidence VI–strength of recommendation B)

1	Obs 36
2	Obs 12
3	Obs 8
4	Obs 6
5	Obs 3
6	PSSE
7	NTRB
8	SIR
9	SSB
10	HTRB
11	PTRB
12	FTRB
13	TTRB
14	Su

Here, we present a Strength of Treatments Scheme (STS) (Table 7) that reports all the possible treatments that can be proposed for Idiopathic Scoliosis starting from the least to the most demanding (both in terms of challenge for the patient, and possible efficacy). In addition, the STS is Consensus based (Level of Evidence V—Strength of Recommendation B). Starting from the STS, it is possible to state, for each single clinical situation of the PAS, a minimum and a maximum of possible treatments that could be proposed: consequently, all treatments that in the STS are reported between this minimum and maximum can be considered for that specific clinical situation. Tables 8 and 9 show the number of paper for each Level of Evidence and the Strength of recommendation for each treatment.

**Table 7:** Strength of treatments scheme (STS) (strength of evidence V–strength of recommendation B): it reports all the possible treatments that can be proposed for idiopathic scoliosis graduated from the less to the most demanding (both in terms of burden on

		Low		Moderate		Severe	
		*Min*	*Max*	*Min*	*Max*	*Min*	*Max*
Infantile		Obs3	Obs3	Obs3	TTRB	TTRB	Su
Juvenile		Obs3	PSSE	PSSE	FTRB	HTRB	Su
Adolescent	Risser 0	Obs6	SSB	HTRB	FTRB	TTRB	Su
	Risser 1	Obs6	SSB	PSSE	FTRB	FTRB	Su
	Risser 2	Obs6	SSB	PSSE	FTRB	FTRB	Su
	Risser 3	Obs6	SSB	PSSE	FTRB	FTRB	Su
	Risser 4	Obs12	SIR	PSSE	FTRB	FTRB	Su
Adult up to 25 y		Nothing	PSSE	Obs12	SIR	Obs6	Su
Adult	No Pain	Nothing	PSSE	PSSE	SIR	Obs12	HTRB
	Pain	PSSE	SSB	PSSE	HTRB	PSSE	Su
Elderly	No Pain	Nothing	PSSE	Obs36	PSSE	Obs12	HTRB
	Pain	PSSE	SSB	PSSE	HTRB	PSSE	Su
	trunk decompensation	Obs6	SSB	PSSE	PTRB	PSSE	Su

**Table 8:** Level of evidence of recommendations: the table shows the number of papers according to the level of evidence for each treatment

	I	II	III	IV	V	VI	Total
Bracing	2	3	3	6	12	1	25
Specific exercises to prevent scoliosis progression during growth	1	1	1	0	8	1	12
Specific exercises during brace treatment and surgical therapy	0	3	0	0	3	0	6
Other conservative treatments	0	0	0	0	2	0	2
Respiratory function and exercises	0	0	0	0	3	0	3
Sports activities	0	0	2	0	3	1	6
Assessment	0	0	1	9	1	3	14
Total	3	7	7	15	32	6	68

**Table 9:** Strength of recommendations: the table shows the strength of recommendation for each treatment

	A	B	C	D	E	Total
Bracing	2	20	3	0	0	25
Specific exercises to prevent scoliosis progression during growth	0	7	5	0	0	12
Specific exercises during brace treatment and surgical therapy	0	2	4	0	0	6
Other conservative treatments	0	0	2	0	0	2
Respiratory function and exercises	0	1	2	0	0	3
Sports activities	0	3	3	0	0	6
Assessment	1	6	4	1	2	14
Total	3	39	23	1	2	68

The PAS has some main characteristics that constitute its strength and justification:

- PAS is proposed to resolve the differences in treatment decisions between different clinicians in their clinical practice. PAS guards against presumably wrong clinical decisions (above maximum: overtreatment, below minimum: undertreatment).
- It reports a real approach, since most clinicians usually choose a variety of treatments for a single patient; the final decision comes after discussion with the patient, and weighting the various risk factors involved in the clinical situation. In fact, the PAS has been developed according to the “Step by Step” Sibilla’s theory [92, 112–115], which states that for each patient, it is mandatory to choose the correct step of treatment, where the most efficacious is also the most demanding. Accordingly, coming to a wrong decision means facing one of the two main mistakes in conservative treatment of idiopathic scoliosis, overtreatment (too much burden on the patient, without improved efficacy) or undertreatment (treatment that leads to little or no efficacy).
- Evidence-Based Clinical Practice is by definition the best integration between the knowledge offered by Evidence-Based Medicine, individual clinical expertise and patients’ preferences [116–118]. Consequently, different clinicians will treat a patient with the same clinical problem differently; the variation can be due to the patient’s preferences or because of the specific expertise of the clinician. Therefore, proposing a definitive clinical approach for a certain clinical situation is problematic. Rather, a range of options should be considered.

## Conservative treatments

All the treatment approaches below are listed in the STS (Table 7) and are presented from the treatments having least impact to those having greatest impact. For more details about each approach, it is possible to refer to the Brace Technology and the Rehabilitation Schools for Scoliosis Series [119, 120] and the Consensus paper on Terminology [121], published by the *Scoliosis and Spinal disorders* journal.

*Nothing (No)*: No treatment is needed.

*Observation (Ob)*: It is the first step of an active approach to idiopathic scoliosis, and it consists of regular clinical evaluation with a specific follow-up period. Timing of this follow-up can range from 2 to 3 to 36–60 months according to the specific clinical situation. Clinical evaluation does not need to include taking radiographs: radiographs are usually performed during alternate clinical evaluations.

*Physiotherapeutic scoliosis-specific exercises (PSSE)*: PSSE include all forms of outpatient physiotherapies with evidence of having an effect on some scoliosis outcomes and which will gradually be published in the Rehabilitation Schools for Scoliosis Series [120] in the *Scoliosis and Spinal Disorders* journal. They have been listed in the 3rd part of these guidelines. The frequency of therapeutic sessions varies from twice to 7 days a week depending on the complexity of the techniques, motivation and the ability of the patient to carry out the treatment. Long-term outpatient physiotherapy sessions usually take place two to four times a week if the patient is willing to cooperate fully. The actual form of exercise depends mainly on the character of the selected therapeutic method.

*Special Inpatient Rehabilitation (SIR)*: If SIR is recommended, patients spend several weeks (usually 3–6) at a specialized health centre (hospital department, sanatorium or a similar form of health care) where they undergo an intensive PSSE treatment (several hours per day).

*Bracing*: It consists of using a brace (a corrective orthosis) for a specified period of time each day. Usually, it is worn until maturity. The main therapeutic goal is to halt the scoliosis curves from progression. According to SOSORT, the use of a rigid brace implies the use of exercises when out of the brace. Bracing includes the following:

- *Night Time Rigid Bracing* (8–12 h per day) *(NTRB)*: wearing a brace mainly in bed.
- *Soft Bracing (SB)*: it includes mainly the SpineCor brace [122, 123] but also other similar designs [124, 125].
- *Part Time Rigid Bracing* (12–20 h per day) *(PTRB)*: wearing a rigid brace mainly outside school and in bed.
- *Full Time Rigid Bracing* (20–24 h per day) *or cast (FTRB)*: wearing a rigid brace all the time (at school, at home, in bed, etc.). Casts have been included here as well. Casts are used by some schools as the first stage to achieve correction to be maintained afterwards with rigid brace [126–128]; a cast is considered a standard approach in infantile scoliosis [129–132]. Recently, a new brace has been developed that has been claimed to achieve same results as casting [91, 133, 134].

A common feature of all forms of conservative treatment is the need to actively involve the patient and caregivers [135]. Therefore, education, psychotherapy, systematic monitoring of outcomes, assessment of patient’s compliance, and verification and modification of methods in the course of the therapy are deemed crucial elements of conservative treatment. In order to achieve the best possible outcome, conservative treatment should be delivered by an experienced therapeutic team including a physician, a physiotherapist, an orthotist and possibly a psychologist [135]. Support groups and Internet forums are also important in conservative treatment.

### Prognostic factors

Prognostic factors should be used with PAS, to select options appropriately between the minimum and maximum strength of treatment. The following factors have been suggested as possible determinants of a higher risk of scoliosis progression: positive family history, laxity of skin and joints (connective tissue defect), flattening of physiological thoracic kyphosis (impedes efficient bracing), angle of trunk rotation exceeding 10°, and growth spurt [136].

Bunnell reported that the risk of progression at the beginning of puberty is 20% in 10° scoliosis, 60% in 20° scoliosis, and as much as 90% in 30° scoliosis [55, 137]. At the age of peak height growth (13 years of osseous age in girls), the risk of progression is 10, 30 and 60%, in the curve severity threshold categories above, respectively. During the final stage of puberty (at least Risser grade II), the risk of deformity progression becomes considerably lower, falling to 2% in 10° scoliosis, 20% in 20° scoliosis and 30% in 30° scoliosis. The prognosis regarding IS progression seems to be more optimistic for boys [138].

Considering that the sagittal spine profile of mild (10°–20°) scoliotic curves was found to be similar to the lateral spine profile of their healthy controls [139], it has been proposed that thoracic hypokyphosis, coupled with axial rotation, could be compensatory rather than etiological in IS pathogenesis [140].

Scoliosis can affect the spine not only through translation in the frontal, and rotation about horizontal plane, but also through changes in the sagittal profile of the spine. Different types of scoliosis present with different sagittal profiles; one example is the typical association of flat back in thoracic scoliosis. Although the etiology of scoliosis is unknown, some authors have hypothesized that patients with certain sagittal spinal profiles seem more prone to developing scoliosis than others [141–145]. It has been demonstrated that the sagittal profile of the spine depends on the pelvic placement playing a major role in determining the sagittal balance of the spine [146–149].

The pathologic mechanism of progression of IS curve is described in recent publications [46, 47, 150, 151]. The factors that contribute to progression include the effect of gravity, the muscle action, the reactive forces causing increased lordosis, the human gait, and the growth induced torsion. The intervertebral disc could be included as an additional morphological factor involved in the progression of an IS curve [120, 152, 153].

Recently developed genetic assessment, with 53 identified loci [56, 154], can now help predict the risk of IS progression. The determination of the polymorphism of selected genes is meant to facilitate the assignment of a patient to a progressive or stable group [155–157]. Unfortunately, the data originating from one population often are not confirmed in replication studies involving other populations [158, 159]. A prognostic genetic test, known as ScoliScore, has also been developed [160]. Although these initial results have been promising, their generalizability is still uncertain [161].

Finally, during recent years, there have been several prognostic formulas that have been proposed [48, 162, 163]. The previous SOSORT guidelines [3] were based on the Lonstein and Carlson factor of progression [48] for the assessment of the risk of idiopathic scoliosis. Since there are no formulas that have been applied in specific studies after their development to verify their real accuracy, we do not apply them in these guidelines.

The wide range of normative values, already demonstrated in large population of healthy children, and the recognized changes of pelvic and sagittal parameters during growth [164, 165] can significantly affect these results and make it very difficult to reach definite conclusion. In addition, curve magnitude influences the sagittal profile of the spine. Therefore, some differences may be related to the mean Cobb angle of the population included in each study. Even though there still remain many unanswered questions, it appears that the sagittal parameters are correlated with the development of the spinal deformities, and we recommend they be monitored during therapy.

## Brace treatment

### Methods

In November 2015, we performed a search in MEDLINE from its inception, with no language limitations. We used the following search strategies:

“Braces”[Mesh] AND “Scoliosis”[Mesh] AND (has abstract[text] AND (Clinical Trial[ptyp] OR Meta-Analysis[ptyp] OR Practice Guideline[ptyp] OR Randomized Controlled Trial[ptyp] OR Review[ptyp])) (198 papers).

(“Scoliosis/therapy”[Mesh]) AND “Braces”[Mesh] AND compliance (100 papers)

“Scoliosis”[Mesh] AND “Braces”[Mesh] AND (“infant, newborn”[MeSH Terms] OR “infant”[MeSH Terms:noexp] OR “child, preschool”[MeSH Terms]) (194 references).

We selected from the titles a total of 250 references, and looking at the abstracts, 102 were selected and retrieved in full text. We also searched the following: the abstracts of all SOSORT meetings, from the first one in 2003 to 2010; the personal files and knowledge of all the authors; the articles retrieved with all the other searches listed in these guidelines; and the references sections of all retrieved papers. The selection criteria used in all these searches were as follows: pertinence for the topic “Brace treatment”; presence of the abstract; numerical results in relation to scoliosis; retrievability in full text; all languages.

### Results

SOSORT has published in *Scoliosis and Spinal Disorders Journal* two Consensus Papers on bracing titled “SOSORT consensus paper on brace action: TLSO biomechanics of correction (investigating the rationale for force vector selection)” [131] and “Guidelines on ‘Standards of management of idiopathic scoliosis with corrective braces in everyday clinics and in clinical research’: SOSORT Consensus 2008” [135]; in addition, previously published guidelines are also freely available in the Journal web page [3], which can serve as reference for specific insights.

#### Efficacy in adolescents

Recently, a Cochrane review and its update [166–168] found that there is very low-quality evidence in favour of using braces, making generalization very difficult. This review included seven articles:

five were planned as RCTs [93, 123, 169–171] and two as prospective controlled trials [90, 172, 173]. One of the RCTs failed due to very low recruitment of participants [174], while another [93] introduced a preference arm for the same reason.

Nachemson multicenter prospective international observational study provided very low-quality evidence in favour of the efficacy of bracing [173]. Nachemson evaluated 240 patients with thoracic or thoracolumbar curves between 25° and 35°, aged between 10 and 15 years, of which 129 were only observed and 111 treated with thoracolumbar braces. Progression of 6 or more degrees at any of 2 radiographic follow-ups was considered failure of the selected treatment (observation versus brace treatment). At 4 years of follow-up, the success rate for brace treatment was 74% (range, 52—84%), whereas the rate for observation was 34% (range, 16—49%).

In prospective trials, the results were in favour of brace [90]: Lusini reported that the rate of success (no progression of 5° or more, no fusion, or no waiting list for fusion) was 25/33 in the brace group and 0/10 in observation group in the per-protocol analysis (RR 15.21, 95% CI 1.00 to 230.23) and 31/39 in the brace group and 8/18 in the observation group in the ITT analysis (RR 1.79, 95% CI 1.04 to 3.07).

A randomized controlled trial demonstrated with very low-quality evidence that a plastic TLSO brace is more effective than an elastic brace [171]. Wong randomized 43 subjects to SpineCor or rigid orthosis group. Although it has been stated that the authors were not trained to fit the SpineCor brace [175], the authors concluded that 68% of the subjects in the SpineCor group and 95% of the subjects in the rigid orthosis group did not show curve progression, with a significant difference in favour or rigid braces. The two groups had similar responses to a patient acceptance questionnaire.

In a randomized controlled trials with a 2 years’ follow-up (116 participants from the randomized cohort), Weinstein found that the mean PedsQL did not differ significantly between bracing [87] and observation groups and found that the rate of success (curves remaining below 50°) was 38/51 in the brace group and 27/65 in the observation group (RR 1.79, 95% CI 1.29 to 2.50).

The Cochrane review concluded that bracing prevented curve progression. The presence of failure of RCTs due to parents rejecting randomization of their children demonstrates important difficulties in conducting RCTs in the field of conservative treatment for scoliosis. Future research should focus on participant outcomes, adverse effects, methods to increase compliance, and usefulness of physiotherapeutic scoliosis-specific exercises added to bracing.

RCTs and prospective cohort studies should be conducted according to pre-defined criteria such as the Scoliosis Research Society (SRS) and the international Society on Scoliosis Orthopedic and Rehabilitation Treatment (SOSORT) criteria.

In fact, beyond the previously reported studies, the SRS defined some methodological criteria to be followed during brace cohort studies [176]. The optimal inclusion criteria consist of: age 10 years or older when brace is prescribed, Risser 0–2, primary curve angles 25°–40°, no prior treatment, and, if female, either pre-menarche or less than 1 year post menarche. Assessment of brace effectiveness should include (1) the percentage of patients who have ≤ 5° curve progression and the percentage of patients who have ≥ 6° progression at maturity, (2) the percentage of patients with curves exceeding 45° at maturity and the percentage who have had surgery recommended/undertaken, and (3) 2-year follow-up beyond maturity to determine the percentage of patients who subsequently undergo surgery. All patients, regardless of subjective reports on compliance, should be included in the results (intent to treat). Every study should provide results stratified by curve type and size grouping. Cohort studies respecting the SRS criteria can be considered of high methodological quality. Until now, 12 papers have been published with these characteristics and 6 of them in the last 4 years [123]. Recently, a consensus statement aimed to establish a framework for research with clearly delineated inclusion criteria, methodologies, and outcome measures to allow better and easier, future meta-analysis or comparative studies was organized in conjunction with the SOSORT and SRS society [177].

Together with these criteria, SOSORT offered the “Standards of management of idiopathic scoliosis with corrective braces in everyday clinics and in clinical research” [135] that include 14 recommendations, grouped in 6 domains (Experience/competence, Behaviours, Prescription, Construction, Brace Check, Follow-up). Cohort studies using the SOSORT criteria can be considered of high quality in terms of patient and treatment management. Until now, six papers have been published with these characteristics [89, 90, 92, 178–185].

With regard to the studies that were conducted using the SRS and/or SOSORT criteria we found:

Janicki et al. [179], following the SRS criteria, retrospectively compared in an “intent-to-treat” analysis the effectiveness of the custom thoracolumbosacral (TLSO) worn 22 h/day and the Providence orthosis worn 8–10 h/night. There were 48 patients in the TLSO group and 35 in the Providence group. In the TLSO group, only 7 patients (15%) did not progress (≤ 5°), whereas 41 patients (85%) progressed by 6° or more, including the 30 patients whose curves exceeded 45°. Thirty-eight patients (79%) required surgery. In the Providence group, 11 patients (31%) did not progress, whereas 24 patients (69%) progressed by 6° or more, including 15 patients whose curves exceeded 45°. Twenty-one patients (60%) required surgery. Nevertheless, the two groups considered were not fully comparable at baseline.

Coillard et al. [178], following the SRS criteria, studied prospectively a cohort of 254 patients treated with the SpineCor brace. Successful treatment (correction > 5° or stabilization ± 5°) was achieved in 165 of the 254 patients (64.9%). Forty-six immature patients (18.1%) required surgical fusion while receiving treatment. Two patients out of 254 (0.7%) had curves exceeding 45° at maturity.

Negrini et al. [92], following both the SRS and SOSORT criteria, retrospectively studied a cohort of 42 females and four males treated according to individual needs, with Risser casts, Lyon or SPoRT braces (14 for 23 h per day, 23 for 21 h/d, and seven for 18 h/d at start). No patient progressed beyond 45°, nor was any patient fused, and this remained true at the 2-year follow-up for the 85% that reached it. Only two patients (4%) worsened, both with single thoracic curve, 25–30° Cobb and Risser 0 at the start.

Aulisa et al. [89], following both the SRS and SOSORT criteria, retrospectively reviewed a cohort of 50 adolescent females with thoraco-lumbar curves treated with the Progressive Action Short Brace (PASB). Curve correction was accomplished in 94% of patients, whereas a curve stabilization was obtained in 6% of patients. No patient required surgery, nor anyone progressed beyond 45°.

Aulisa et al. [184] following both the SRS and SOSORT criteria retrospectively reviewed a cohort of 40 adolescent females with lumbar curves treated with the Progressive Action Short Brace (PASB). Curve correction was accomplished in 82.5% of patients, whereas curve stabilization was obtained in 17.5% of patients. None of the patients experienced curve progression.

Gammon et al. [180], following the SRS criteria, compared treatment outcomes of two cohorts of patients treated via either a conventional rigid thoracolumbosacral orthoses (TLSO: 35 patients) or a SpineCor non-rigid orthosis (32 patients). No significant difference was found using the more strict outcome measure (≤5° curve progression) as the success rates were 60% for TLSO and 53% for SpineCor. Looking at patients who reached 45°, the success rates were 80% for TLSO and 72% for SpineCor with no significant difference. Guo et al. [186] following SRS criteria studied two groups: SpineCor (*n* = 20) or rigid brace (*n* = 18). Before skeletal maturity, 7 (35.0%) patients in the SpineCor Group and 1 (5.6%) patient in the Rigid brace group had curve progression > 5°.

Zaborowska-Sapeta et al. [187], including patients according to the SRS criteria, prospectively followed 79 patients treated with Cheneau brace. At 1 year after weaning the brace, they found improvement in 25.3%, stabilization in 22.8%, progression of the Cobb angle up to below 50° in 39.2% and progression beyond 50° in 12.7%; the latter was considered surgical indication.

Aulisa et al. [183] following both the SRS and SOSORT criteria, studied prospectively 163 patients treated with PASB, Lyon brace and Milwaukee affected by juvenile idiopathic scoliosis. Curve correction was accomplished in 88 patients (77.8%); stabilization was obtained in 18 patients (15.9%). Seven patients (6.19%) have a progression and 4 of these were recommended for surgery. Of 26 patients who abandoned the treatment, at the time of abandonment (12.5 age), 19 cases (70.0%) have achieved curve correction, 5 cases (19%) stabilized, and 3 cases (11%) progressed.

Negrini et al. [181], in a prospective cohort study of 73 patients, treated with the Sforzesco brace, following both the SRS and SOSORT criteria, reported 34 patients (52.3%) improved; seven (9.6%) worsened, of which 1 progressed beyond 45° and was fused and employing intent-to-treat analysis, there were failures in 11 patients (15.1%).

Finally, Aulisa et al. [182] following both the SRS and SOSORT criteria, studied a cohort of 102 patients treated with Lyon Brace, who were drawn from a prospective database and found the following: 69 patients had a definite outcome, 17 have abandoned treatment and 16 are still in treatment. Curve correction was accomplished in 85.5% of patients, curve stabilization was obtained in 13% of patients and curve progression was evident in only 1.5%. None of the patients were recommended surgery post-bracing. Of 17 patients who abandoned the treatment, at the time of abandonment (14.4 age), 13 cases (77%) have achieved curve correction, 53 cases (18%) stabilized, and 1 case (5%) progressed.

In summary, these studies show a high variability among the results of bracing [90, 92, 178–184, 187, 188]. This is particularly high for rigid bracing [90, 92, 178–184, 187, 188] despite the results of the treatment being better in the recent studies [90, 181–184]. The soft braces [122, 123, 178, 180] can have a high variability of results, from better to worst [179, 180], as compared to some types of rigid braces; the best results have been achieved with the rigid once, when applying the SOSORT criteria [92, 181–184, 187, 188]. It must also be noted that high variability can be found between different publications in the type of scoliosis treated, thus a different outcome in treatment.

Recently, Weinstein et al. [87] performed a randomized controlled study, but the trial was stopped early owing to the efficacy of bracing, the rate of treatment success was 72% after bracing, as compared with 48% after observation. The authors concluded that bracing significantly decreased the progression of high-risk curves to the threshold for surgery in patients with adolescent idiopathic scoliosis. This study is in contrast with results [189] of a systematic review published earlier by Dolan. The systematic review included only studies written in English, if observation or a TLSO was evaluated and if the sample closely matched the current indications for bracing (skeletal immaturity, age 15 years or less, Cobb angle between 20° and 45°). Eighteen studies were included (3 observation only, 15 bracing). Despite some uniformity in surgical indications, the surgical rates were extremely variable, ranging from 1 to 43% after bracing, and from 13 to 28% after observation. When pooled, the bracing surgical rate was 23% compared with 22% in the observation group. It was concluded that, based on the evidence presented, one could not recommend one approach over the other to prevent the need for surgery in AIS. The use of bracing relative to observation was said to be supported by “troublingly inconsistent or inconclusive studies of any level”. The article inclusion criteria used by Dolan resulted in the exclusion of some retrospective bracing studies, since they had used exercises together with bracing [190–192]. Studies having used exercises and bracing are summarized here.

Weiss [192] considered 343 scoliosis patients (females only) of various etiology, with a mean curvature of 33.4°. Forty-one patients (12%) had had surgery. In patients with adolescent idiopathic scoliosis, the incidence of surgery was 7.3%.

Rigo [190] considered 106 patients with curves on average of 30° at start, out of which 97 were followed up until the end of growth, and six cases (5.6%) ultimately underwent spinal fusion. A worst case analysis, which assumes that all nine cases that were lost to follow-up had operations, brings the uppermost number of cases that could have undergone spinal fusion to 15 (14.1%).

Maruyama [191] reviewed 328 females with an average 32.4° Cobb angle. Surgery was recommended when curvature progressed to > 50°. Twenty (6.1%) were treated with spinal fusion. The remaining showed no significant increase in magnitude of curvature.

In 2008, Negrini [112] reported on surgery rates in curves over 30° at first evaluation, treated with brace and exercises: they were a subgroup of 28 out of 112 patients with a mean 23.4 Cobb degrees at the start of treatment. The rate of surgery was 1.9% (efficacy analysis) and 9.1% (worst case) versus 0.9 and 4.5% respectively in the whole group observed.

Some years ago, Rowe [193] conducted a meta-analysis to compare the consistency of outcomes among several of the oldest studies. Of a total of 1910 patients, 1459 received brace treatment, 322 electrostimulation, and 129 only observation. The weighted mean success rate was 0.39 for electrostimulation, 0.49 for observation, 0.60 for braces worn 8 h daily, 0.62 for braces worn 16 h daily, and 0.93 for braces worn 23 h daily, the last of which was the statistically most efficacious treatment method. The most effective brace system was the Milwaukee brace vs. others, while the Charleston brace, which was worn only during night, was the least successful, but statistically better than observation alone.

#### Are there braces that are better than others?

In the literature, there are very few studies comparing different braces. Zaina et al. [194] conducting a Dephi consensus with SOSORT and SRS experts showed the state of art about the braces. SOSORT experts could not reach consensus as to how the best possible correction can be achieved through bracing [135]; while the importance of the three point system mechanism was emphasized, options about proper pad placement on the thoracic convexity were divided 50% for the pad reaching or involving the apical vertebra and 50% for the pad acting caudal to the apical vertebra. There was agreement about the direction of the vector force, 85% selecting a “dorso lateral to ventro medial” direction, but not about the shape of the pad to produce such a force. Principles related to three-dimensional correction achieved high consensus (80–85%) but suggested methods of correction were quite diverse. This situation is reflected in the different corrective systems used throughout the world.

Looking at studies comparing different braces, we found an RCT [171] pointing out a TLSO more effective than SpineCor; one meta-analysis [193] in favour of the Milwaukee brace with Charleston being the less efficacious; one systematic review [189] finding the following pooled surgery rates: Boston Brace 12–17%; various braces (Boston-Charleston-TLSOs) 27–41%; nighttime braces (Providence or Charleston braces) 17–25%; TLSO or Rosenberg brace 25–33%; and Wilmington 19–30%. Three retrospective studies also addressed this question: one [179] obtained the best results with the Providence night-time orthosis over a TLSO, the second [180] reported equal results with a rigid TLSO and SpineCor, and the third [123] reported better results with rigid brace than SpineCor. After reviewing the literature, we also found an old study by Bunnell [195] reporting similar results with a TLSO and Milwaukee brace in a preliminary retrospective study, while Montgomery’s study [151] reported that the Boston Brace was more successful than the Milwaukee brace irrespective of initial curve magnitude and skeletal maturity. Katz [196] compared the Boston Brace to the Charleston bending brace: the former was more effective than the latter, both in preventing curve progression and in avoiding the need for surgery. These findings were most notable for patients with curves of 36° to 45°, in whom 83% of those treated with a Charleston brace had curve progression of more than 5°, compared with 43% of those treated with the Boston Brace.

Howard et al. [197] presented a retrospective cohort study on 170 patients who completed brace treatment: proportion of patients with more than 10° of curve progression was 14% with TLSO, 28% with Charleston, and 43% with Milwaukee brace while those who underwent surgery were 18, 31, and 23% respectively. Weiss [192] compared the survival rates of the Cheneau versus SpineCor by considering as time point event the curve progression. The duration of treatment during pubertal growth spurt in the two included cohorts of patients was also investigated with a prospective follow-up. At 24 months of treatment, 73% of the patients with a Cheneau brace and 33% of the patients with the SpineCor were still under treatment with their original brace; at 42 months, the same percentages were 80 and 8%, respectively.

Yrjonen [198] studied retrospectively the Providence nighttime brace used by 36 consecutive female with lumbar and thoracolumbar scoliosis curves of less than 35°: progression of the curve > 5° occurred in 27%, versus 22% of 36 matched patients treated with the Boston full-time that progressed.

Negrini [199] compared the classical Lyon brace to the newly developed Sforzesco brace, based on the SPoRT concept (Symmetric, Patient-oriented, Rigid, Three-dimensional, active) in a prospective, matched pairs controlled study. All radiographic and clinical parameters decreased significantly with treatment in both groups, apart from thoracic Cobb degrees with the Lyon brace. The Sforzesco brace had better results than the Lyon brace radiographically, for sagittal profile, aesthetics, and patient recovery (12 improved and 3 unchanged vs 8 and 5).

Negrini [133] also studied a prospective cohort who had refused surgery treated with the Sforzesco brace compared to a Risser cast retrospective control group. Results were comparable between the two groups, with only minor differences in terms of scoliosis correction. On the contrary, straightening of the spine (decrease of the sagittal physiological curves) was much higher with the cast, while it was not clinically significant with the brace.

De Mauroy [200] compared the ART brace with Lyon brace. A prospective case series of 148 scoliosis with short time results after 1 year, treated with the ART brace, was compared with a historical retrospective case series of 100 patients with scoliosis treated with Lyon brace. This study demonstrated that the ART brace had better radiographic results than the Lyon brace and this trend was maintained further at 6 months and at 1 year.

Zaina et al. [185] compared the short-term radiographic results of two super-rigid braces, the ART and the Sforzesco brace, and showed similar results, despite the better in-brace correction for lumbar curves shown by the ART brace.

All these studies are not directly comparable because there are differences in the eligibility criteria and in the main endpoint used to define results. Moreover, in comparative studies, the specific competence in making a specific brace can play a major role [175]. In this respect, even if it is not considered a good standard, comparison with historical controls treated with braces used before by the same treating team can offer good insights [133, 179, 180, 198, 199]. Today, it is not possible to state with any certainty which brace is better than the other, and this is one of the reasons that drove the official publication of SOSORT to develop the Brace Thematic Series [119], where the different concepts are presented to allow a good comparison and a greater understanding of these treatment instruments [126, 201–204]. Nevertheless, it is already possible to see some trends: new alternative concepts have been developed trying to substitute the most invasive braces: this was true some years ago for TLSOs instead of Milwaukee, more recently for night time bending braces or SpineCor instead of TLSOs, and in the last years for the Sforzesco brace and ART brace instead of casting; not all these new concepts have been able to prove their efficacy. In the meantime, there are continued efforts to progressively refine and strengthen some old concepts, like the Cheneau, Boston or Lyon braces, but also newly developed ones, like PASB, Sforzesco, ART and SpineCor brace.

In summary, examining all these studies in adolescent patients, it is clearly evident that something beyond the instrument (brace) plays a role in final results. These factors can include dosage, quality of bracing, compliance to treatment [87, 106, 136, 205–208], family history, type of scoliosis and even geographical distribution, but also team approach [135] that we will briefly review below.

##### Dosage, compliance and quality of bracing

In a review on dose effect, Dolan and colleagues did not find differences among the groups wearing the brace 16–18 h (19–34% surgery rate), 18–23 h (21–26%), and night time (17–25%) [189]; these results were improved with the BrAIST randomized controlled study conducted by the same authors a few years later. Objective monitoring of hours of brace wear allowed showing a correlation between dosage and effects of brace intervention [87]. In 1984, a meta-analysis by Rowe [193] suggested that the 23-h regimens were significantly more successful than any other treatment, while the difference between the 8- and 16-h regimens was not significant: it must be noted that the limitations of this meta-analysis were recognized by the authors and were quite important. Allington and Bowen [209] reported no differences between full-time and part-time brace prescription both in curves below 30° and between 30° and 40°; Katz et al. [210] has been able to check the real use of the brace by the patient through a heat sensor. A logistic regression analysis showed a “dose-response” curve in which the greater number of hours of brace wear correlated with lack of curve progression. Curves did not progress in 82% of patients who wore the brace more than 12 h per day, compared with only 31% of those who wore the brace fewer than 7 h per day. As a result, dosage can be considered a possible major factor in explaining some of the results of bracing: in fact, it has been shown that the more hours of daily brace weaning, the more the deformity collapses (“concertina effect”) [211].

More recently, Aulisa et al. [106] prospectively evaluated the association between compliance of brace treatment and the progression of the scoliotic curve in 522 patients with idiopathic adolescent (AIS) or juvenile scoliosis (JIS). He showed that using the brace full time (22 to 18 h) may alter the natural history of AIS and JIS and curve progression and that referral to surgery are lower in patients with high brace compliance. The type of brace influences the compliance, such that adherence to treatment is higher with the PASB than the Lyon or Milwaukee brace. Interestingly, AIS patients show a better compliance to bracing than those with JIS. Wearing the brace only overnight and bracing discontinuation up to 2 months a year is associated with a high rate of curve progression.

Adherence to treatment is the second main issue to be considered. Many studies have underlined that reported compliance is correlated with final results [106, 207, 210, 212, 213]. Compliance to bracing has been correlated to quality of life and psychological issues [174, 214–217]. Since patients during clinical evaluations overstate their adherence to treatment [218], heat sensors have been developed to check real compliance: it has been confirmed that both reported and estimated hours of brace wearing are inaccurate [218–224] and that compliance is not correlated with the hours of bracing prescribed [223]. Nighttime wear is more accepted than daytime [225], and a “dose-response” to bracing seems to be confirmed [210, 226]. It has also been proposed that it is possible to develop a progression model in single patients with a formula including the risk of progression at the beginning of brace treatment, plus the use in terms of brace tightness and wear time [222]. It is clear that, since patients are not fully compliant, bracing appears not effective. SOSORT propose that compliance should be considered in terms of management of patients: in this perspective, adherence to treatment is a characteristic neither of the treatment only, nor of the patient alone, but of the good interaction between these two factors, based on the active approach by an expert treatment team able to reduce the burden of the brace and increase the coping abilities of the patient [135, 227]. A setting with great attention to the patient and his family and a team approach is able to enhance compliance, thus allowing very good adherence even with fulltime brace wear as demonstrated by research based on the use of compliance monitor [228]. Mainly for these reasons, SOSORT proposes its recommendations [120].

Finally, another important factor is the quality of bracing. There is good agreement to judge it according to the in-brace correction [136, 210, 212, 229–234], even though the currently reported percentages of correction, showed a significant variability from 20 to 25% to 40–50%. Furthermore, the in brace correction is considered as prognostic factors for final good results and it has become, on one hand, the starting point to develop new braces [85, 202, 203, 235–239] and, on the other, a biomechanical reference for various studies [232, 239–241]. Recently, a finite element model study confirmed the importance of immediate in-brace correction to predict long-term outcome of bracing treatment [242]. Other factors such as the absolute reduction of the Cobb angle (i.e. in rigid curves over 50°) or 3D correction might also be important and should be considered in the future [243]: in fact, it is still possible that a great in-brace reduction corresponds to a worsening of other parameters, e.g. in the sagittal plane, finally driving to a flat-back and worse functional results [133, 146] . In this respect, it is mandatory not to confuse the in-brace correction with the success of an orthotic treatment. While in-brace correction studies should be considered preliminary, only results at the end of treatment and/or at minimum of 1–2 years post treatment follow-up should be regarded as proof of efficacy. In any case, according to the actual knowledge, in-brace correction should be regarded as a way to individually judge the quality of the brace applied to single patients.

All the criteria for inclusion, exclusion, and outcome have some drawbacks; one main problem is the fact that even the noncompliant patients are to be included in the studies and it seems that this is one of the criteria that is most frequently “forgotten”. In this situation, it is extremely difficult to compare two different studies and often the professional trying to offer the best treatment for his patients has the difficult task of comparing “apples to oranges”. Apart from the inclusion and exclusion criteria as well as the assessment of brace effectiveness proposed by the SRS Committee, a few more guidelines for future studies should be proposed. All patients that accepted the treatment in a given time period should be included in the study regardless of their compliance. Patients that have withdrawn from the treatment (changed the type of treatment, had surgery recommendation, etc.), regardless of their outcome, should be considered as failure of that specific treatment. All the patients that accepted a specific treatment should be followed up for at least 1–2 years after the completion of treatment, and measurements should be taken at the beginning of the treatment, at the weaning point and at follow-up.

##### Efficacy in other populations

Adolescent idiopathic scoliosis with curves below 40–45° and still growing is the main population targeted by brace treatment [189], but it has been applied as well in other populations, that we will briefly review here.

In juvenile idiopathic scoliosis, historically, the percentages of surgery after treatment with braces ranged widely, with Tolo [244] reporting 27.2%, Figueiredo [245] 62%, Mannherz [246] 80%, McMaster [247] 86%, and Kahanovitz [248] 100%. This clearly demonstrates the difficulty in this specific population, where the expected progression rate could range between 70 and 95% [122]. Coillard [123] reported that, with the SpineCor brace, out of 67 patients with a definite outcome, 32.9% corrected their Cobb angle by at least 5° and 10.5% had a stabilization of their Cobb angle, while 37.3% of patients where recommended for surgery before the authorized end of treatment (before skeletal maturity). Results depended on the amplitude of the Cobb angle: 26.3% of the patients with curves under 25° eventually needed surgery while 51.8% of the second group (> 25°) had surgery recommended. Finally, Fusco et al. [249] found a percentage of 9% of juvenile patients treated conservatively who finished treatment over 45°.

More recently, Aulisa et al. [183] reported in a prospective study, out of 163 patients, with juvenile idiopathic scoliosis, treated with PASB, Lyon brace and Milwaukee, that the 113 patients with a definite outcome curve correction were accomplished in 77.8% and 6.19% have a progression and 3.3% were recommended for surgery. The treatment appears to be more effective with curves under 30° (incidence of surgery: 1.6%) than curves over 30° (incidence of surgery: 5.5%) but compared to the natural history of disease both are better.

Also in infantile idiopathic scoliosis reported, results are quite variable, as well as the treatment applied: serial casting is the most advocated [94, 129, 250–252], but bracing alone has also been used [237–239, 247], mainly the Milwaukee brace [247, 251, 252]. The few case series reported generally include small numbers of patients with variable results, from a 100% surgery rate [53], to around 50% [250] or much less [251, 253] (mainly if casts are used [250]). Mehta reported the largest case series of 136 children followed up for 9 years: 94 children, referred and treated in the early stages (mean age 19 months—6 to 48, Cobb angle 32°–11° to 65°), resolved the deformity by a mean age of 3 years and 6 months, with no need of further treatment; 42 children, referred late (mean age 30 months—11 to 48, Cobb angle 52°–23° to 92°), reduced but not did not reverse scoliosis; 15 children (35.7%) were fused. The hypothesis of the author was that scoliosis can be reversed by harnessing the vigorous growth of the infant to early treatment by serial corrective plaster jackets [129].

Like in the adolescent type, puberty is the worst period for infantile scoliosis, for scoliosis progression [251]. Single thoracic curves seem to have the worst outcomes when compared to double structural ones [247]. It has also been reported that best results are obtained in progressive types if treatment is started when the angulation is still under 30° [253], or 60° and younger age [252], again mainly with casting [250]. Scoliosis is considered resolved or stabilized non-operatively at an acceptable Cobb angle, within a normal range of cosmesis, and within a normal range of pulmonary function. This is not the case for those patients who have been treated surgically [245].

Finally, two papers recently focused on other groups. In those with scoliosis over 45° who refused to be operated, Negrini et al. [91] reported, out of 28 patients (curve range 45–58° Cobb) who reached the end of treatment (brace and exercises for 4.5 years), two patients (7%) remained above 50° but six patients (21%) finished between 30° and 35° and 12 patients (43%) finished between 36° and 40° Cobb. Improvements have been found in 71% of patients and a 5° Cobb progression in one patient. Lusini et al. [172] studied 39 patients (BG) with a full-time brace treatment, 18 patients (CG) that refused any treatment served as controls, failures were 23.5% in BG and 100% in CG and conclude that the conservative brace treatment is a suitable alternative for those patients who reject any surgical intervention for IS above 45.

Aulisa et al. [254] reported that the surgical rate of scoliosis was 15.4% but underlined that in subgroups with rotation < 20°, 98.1% showed a correction/stabilization and 1.8% received surgery referral, while in subgroups with rotation > 25°, a correction/stabilization was achieved in 69.4% but surgery referral in 60.8%.

In a case series of scoliosis subjects at Risser score 4–5, with up to 20 years of age [255] (the residual growth was 0.9 cm), of 23 patients requiring treatment both for esthetic reasons, or to try to reduce the deformity, curve improvements were found in 48% and an improvement in the Aesthetic Index was observed in 30% of the included patients.

##### Team role in bracing

SOSORT already produced a set of Recommendations in the paper “Standards of management of idiopathic scoliosis with corrective braces in everyday clinics and in clinical research” [135], grouped in 6 domains: Experience/competence, Behaviours, Prescription, Construction, Brace Check, and Follow-up. These recommendations, integrally reported below, constitute part of these guidelines.

Recommendation 1 (Experience-competence)

The MD responsible for the treatment has to be experienced and should fulfill all these requirements: training by a previous master (i.e. MD with at least 5 years of experience in bracing) for at least 2 years; at least 2 years of continuous practice in scoliosis bracing; prescription of at least 1 brace per working week (~ 45 per year) over the last 2 years; and evaluation of at least 4 scoliosis patients per working week (~ 150 per year) over the last 2 years.

Due to the situation of conservative treatment in many countries, this must be considered the ideal to be reached as soon as possible through education. Nevertheless, it must be recognized that experience and preparation is an important way to avoid problems to patients and reach adequate results in this field.

Recommendation 2 (Experience-competence)

The CPO constructing braces has to be experienced and should fulfill all these requirements: working continuously with a master MD (i.e. a MD fulfilling to recommendation 1 criteria) for at least 2 years; at least 2 years of continuous practice in scoliosis bracing; and construction of at least 2 braces per working week (~ 100 per year) in the last 2 years.

Due to the situation of conservative treatment in many countries, this must be considered the ideal to be reached as soon as possible through education. Nevertheless, it must be recognized that experience and preparation is an important way to avoid problems to patients and reach adequate results in this field.

Recommendation 3 (Behaviours)

To ensure optimum results, the MD, CPO, and physiotherapist (PT) must work together as an interdisciplinary team. This can be accomplished, even if they are not currently located in the same workplace, through continuous exchange of information, team meetings, and verification of braces face to face with patients.

Recommendation 4 (Behaviours)

Commitment, time, and counseling to increase compliance: MDs, CPOs and PTs must give thorough advice and counseling to each individual patient and family each time it is needed (at each contact for MDs and CPOs) provided they give, as a team, the same messages previously agreed upon.

Recommendation 5 (Behaviours)

All the phases of brace construction must be followed for each single brace prescription by a well-trained and experienced MD (fulfilling recommendation 1 criteria), construction by a well-trained and experienced CPO (fulfilling recommendation 2 criteria) checked by the MD in cooperation with the CPO, and possibly the PT correction by the CPO according to MD indications follow up by the CPO, MD, and PT.

Recommendation 6 (Prescription)

The use of brace is recommended in patients with evolutive idiopathic scoliosis above 25° during growth; in these cases, PSSE alone (without bracing) should not be performed unless prescribed by physicians expert in scoliosis.

Recommendation 7 (Prescription)

In each prescription of a brace (case by case), the MD must write the details of brace construction (where to push and where to leave space, how to act on the trunk to obtain results on the spine) when not already defined “a priori” with the CPO prescribing the exact number of hours of brace wearing be totally convinced of the brace proposed and committed to the treatment use any ethical means to increase patient compliance, including thorough explanation of the treatment, using aids such as photos, brochures, and video.

Recommendation 8 (Construction)

In each construction of a brace, case by case, the CPO has to check the prescription and its details and eventually discuss them with the prescribing MD, if needed, before construction fully execute the agreed prescription be totally convinced of the brace proposed and committed to the treatment use any ethical means to increase patient compliance, including thorough explanation of the treatment, using aids such as photos, brochures, and video.

Recommendation 9 (Brace Check)

In each check of a brace, case by case, the responsible MD in partnership with the CPO has to verify accurately if it fits properly and fulfills the needs of the individual patient, check the scoliosis correction in all three planes (frontal, sagittal and horizontal), check clinically the esthetic correction maximize brace tolerability (reduce visibility and allow movements and activity of daily life as much as possible for the chosen technique), apply all changes needed and, if necessary, even rebuild the brace without extra-charge for patients, check the corrections applied, check that the patient (and/or his/her parents) is able to apply or put on the brace properly, assess the patient’s mood, and counsel the family at brace delivery and at other follow-ups.

Recommendation 10 (Brace Check)

The check of each brace must be a clinical and/or radiographic check.

Recommendation 11 (Follow-up)

The MD, CPO, and PT must check the brace and patient compliance regularly (MDs and CPOs each time they see the patient) and reinforce the usefulness of brace treatment to the patient and his/her family.

Recommendation 12 (Follow-up)

The MD has to follow-up the braced patient regularly, at least every 3 to 6 months. Standard intervals have to be adjusted according to individual needs (first brace, growth spurt, progressive or atypical curve, poor compliance, request of other team members—CPO, PT, etc.). Using tools (written protocols, recalls, etc.) to keep patients informed of their follow-up is strongly suggested.

Recommendation 13 (Follow-up)

The brace has to be changed for a new one as soon as the child grows or the brace loses efficacy, and this need can be suggested by the CPO, but is the responsibility of the treating MD.

Recommendation 14 (Follow-up)

The CPO has to regularly check the brace. To avoid any problems, he/she has to refer to the treating MD.

Recommendation 15 (Follow-up)

The PT has to check the brace regularly. To avoid any problems, she/he has to refer to the treating MD. As a member of the treating team, he/she has to be trained to face the problems of compliance or the needs for more explanation by the patient or his/her family. In case she/he is not entirely a member of the treating team, the PT must not act autonomously and must refer to the treating MD.

##### Other issues

It is not possible in this review of the literature to fully consider all the complex and currently debated topics. For example, with regard to CAD-CAM versus plaster molding in brace construction, research is reaching the conclusion that the way in which the brace is constructed does not interfere with final results, nor with patients’ sensations [229, 236, 238, 256]. Models on finite element modeling of brace efficacy are showing the efficacy of bracing in reducing spinal load and applying corrective moments to the spine; moreover, they are helping in refining brace construction, but there is still a more research needed [232, 241, 243, 257, 258]. Some more years are needed to reach the first clinically useful applications of 3D classifications and understand their effect on brace construction and results’ evaluation [72, 74–76, 79, 80].

Table 10 summarizes the recommendation on bracing.

**Table 10:** Recommendation on bracing

Recommendation	Strength	Evidence	References
1. Bracing is recommended to treat adolescent idiopathic scoliosis	B	I	[87, 90, 91, 122, 123, 166–168, 178–180, 188]
2. Bracing is recommended to treat juvenile and infantile idiopathic scoliosis as the first step in an attempt to avoid or at least postpone surgery to a more appropriate age	B	III	[53, 94, 122, 244–252, 487, 488]
3. The use of brace is recommended in patients with evolutive idiopathic scoliosis above 25° during growth; in these cases PSSE alone (without bracing) should not be performed unless prescribed by a physicans expert in scoliosis.	B	I	[87, 90, 91, 122, 123, 166–168, 178–180, 188]
4. Casting (or rigid bracing) is recommended to treat infantile idiopathic scoliosis to try stabilizing the curve	B	IV	[129, 250, 252]
5. It is recommended not to apply bracing to treat patients with curves below 15° ± 5° Cobb, unless otherwise justified in the opinion of a clinician specialized in conservative treatment of spinal deformities	B	V	
6. Bracing is recommended to treat patients with curves above 20° ± 5° Cobb, still growing (Risser 0 to 3), and with demonstrated progression of deformity or elevated risk of worsening, unless otherwise justified in the opinion of a clinician specialized in conservative treatment of spinal deformities	B	I	[87, 92, 123, 166, 167, 178–180, 188, 189]
7. Very hard rigid bracing (casting) is recommended to treat patients with curve between 45° and 60° to try avoiding surgery.	C	IV	
8. It is recommended that each treating team provide the brace that they know best, which means the brace they are more experienced and with perceived outcomes. This is due to the actual knowledge; there is no brace that can be recommended over the others.	C	IV	[171, 179, 180, 189, 193]
9. It is recommended that braces are worn full time or no less than 18 h per day at the beginning of treatment, unless otherwise justified in the opinion of a clinician specialized in conservative treatment of spinal deformities	B	II	[87, 106, 193, 207, 210]
10. Since there is a “dose-response” to treatment, it is recommended that the hours of bracing per day are in proportion with the severity of deformity, the age of the patient, the stage, aim and overall results of treatment, and the achievable compliance	B	II	[87, 106, 193, 207, 210]
11. It is recommended that daily brace wear is proportionate to the deformity severity, age of patient, scoliosis stage, aim and overall results of treatment, and the expected compliance	B	II	[87]
12. It is recommended that braces are worn until the end of vertebral bone growth and then the wearing time is gradually reduced, unless otherwise justified in the opinion of a clinician specialized in conservative treatment of spinal deformities	B	V	
13. It is recommended that the wearing time of the brace is gradually reduced, while performing stabilizing exercises, to allow adaptation of the postural system and maintain results	B	IV	[112, 190, 191, 290, 489]
14. It is recommended that any mean is used to encourage compliance, including a careful adherence to the recommendations defined in the SOSORT Guidelines for Bracing Management	B	IV	[135, 219–224, 226, 228, 490, 491]
15. It is recommended that compliance to bracing is regularly checked through compliance monitor devices.	B	V	[259, 261, 262, 315, 491]
16. It is recommended that quality of the brace is checked through an in-brace X-ray	B	IV	[136, 205, 212, 229–233, 315]
17. It is recommended that the prescribing physician and the constructing orthotist are experts according to the criteria defined in the SOSORT Guidelines for Bracing Management	C	VI	[135]
18. It is recommended that bracing is applied by a well-trained therapeutic team, including a physician, an orthotist and a therapist, according to the criteria defined in the SOSORT Guidelines for Bracing Management	B	V	[135]
19. It is recommended that all the phases of brace construction (prescription, construction, check, correction, follow-up) are carefully followed for each single brace according to the criteria defined in the SOSORT Guidelines for Bracing Management	B	V	[135]
20. It is recommended that the brace is specifically designed for the type of the curve to be treated	B	V	
21. It is recommended that the brace proposed for treating a scoliotic deformity on the frontal and horizontal planes should take into account the sagittal plane as much as possible	A	V	
22. It is recommended to use the least invasive brace in relation to the clinical situation, provided the same effectiveness, to reduce the psychological impact and to ensure better patient compliance	A	V	
23. It is recommended that braces do not so restrict thorax excursion in a way that reduces respiratory function	B	V	
24. It is recommended that braces are prescribed, constructed and fitted in an out-patient setting	B	V	
25. It is recommended that braces are regularly changed according to growth and/or specific pathological needs as judged by a scoliosis expert physician	B	V	
26. It is recommended that out of brace X-rays are regularly performed to check the effectiveness of bracing treatment: the number of hours out of brace before x-ray taking should correspond to the daily weaning time	B	V	

## Conservative treatments other than bracing

### Physiotherapeutic scoliosis-specific exercises (PSSE) to prevent scoliosis progression during growth

#### Methods

In December 2015, we performed a search in MEDLINE In-Process & Other Non-Indexed Citations and Ovid Medline(R) from 1946, EMBASE 1974 to 2015 week 48, SPORTDiscus with full text from inception, CINAHL Plus with full text from 1937, CENTRAL, and PEDro with no language limitations. The search strategy for each database is presented in Additional file 1. We identified additional published, unpublished, and ongoing studies by hand-searching reference lists of relevant reviews, and abstracts from SOSORT Meetings (2003 to 2015), as well as by contacting topic specialists. Inclusion criteria were as follows: randomized controlled and prospective controlled cohort studies investigating the effect of exercises (any type); effect of exercises during brace and surgical therapy; other conservative treatments; or sport on any scoliosis outcome, presence of an abstract, and usable numerical data. The search yielded 1760 references. After screening the titles and abstracts, 128 references were considered of interest and retrieved in full text. Of those, 7 primary studies met the inclusion criteria and were used to inform this updated guideline on PSSE to prevent scoliosis progression during growth. Previous update of guidelines included an additional 41 references, but the inclusion criteria were slightly different. Here, we are focusing only on study designs considered to provide the most valid estimates (randomized and non-randomized clinical trials).

#### Results

Two previous consensus guidelines were published by SOSORT, titled “Physical Exercises in the Treatment of Idiopathic Scoliosis at Risk of brace treatment - SOSORT Consensus paper 2005” [259] and “2011 SOSORT guidelines: Orthopaedic and Rehabilitation treatment of idiopathic scoliosis during growth” [3], which can serve as reference for specific insights.

SOSORT experts agree that PSSE should consist of the following:

- Auto-correction in 3D
- Training in activities of daily living (ADL)
- Stabilizing the corrected posture
- Patient education

Several systematic reviews, including a Cochrane systematic review on the effects of exercises for scoliosis [260–264], report promising results, but highlight the need for stronger study designs. Those reviews suggest that PSSE slowed the progression (deterioration) of scoliosis and/or reduced curve severity measured by the Cobb angle [264–266]. Some studies also showed improved neuromotor control, [267, 268] respiratory function, [269] back muscle strength, and cosmetic appearance [269]. Lenssinck’s earlier review concluded that the exercises may have positive effect on the scoliosis outcome, but more evidence was needed. The 2012 review by Mordecai and Dabke was an independent review of 110 publications [270] and included nine prospective cohort studies, of which only three were controlled and only one used observer blinding. The authors indicated that selection criteria, recommendations, and contraindications to exercise were not clearly determined in any of these publications. Moreover, most exercise studies did not report on compliance, intention-to-treat analyses, or on recruitment strategies. The magnitude of changes in the Cobb angles was usually statistically significant, but often within the range of measurement error. Three systematic reviews published by the SOSORT members [262, 271, 272] evaluated studies of all designs in terms of the effect of specific exercise programmes in reducing the progression of idiopathic scoliosis. These reviews found that the methodology used in published studies was generally of poor quality, although all but one study (the oldest one) [273] showed positive effect of the exercises on the scoliosis parameters [192, 267, 269, 272, 274–283]. The authors of these systematic reviews concluded that PSSE may be proposed to patients.

In the Cochrane review on the effect of exercises on Cobb angles in patients with AIS, which used the same study selection criteria as in the review conducted for this guideline update (randomized controlled and prospective controlled cohort studies) [261], only two studies were included. The first study was a randomized controlled trial (RCT) by Wan et al. [272]. The authors reported improvements with scoliosis-specific exercises added to surface electrical stimulation on the Cobb angle in patients with scoliosis. All patients received electrical stimulation on the lateral surface of the body, traction, and postural training, while the experimental group also underwent scoliosis-specific asymmetric strengthening exercises once a day. Eighty Chinese patients (40/group), aged 15 ± 4 years, were treated over a 6-month period. Both groups improved, but a larger effect was observed in the exercise group. This study was considered to provide low-quality evidence in favour of exercises used together with other treatments [274]. The other study included in the Cochrane review was Negrini et al.’s cohort observational prospective trial with a concurrent control group [272]. The authors found that 1 year of the PSSE, consistent with the Scientific Exercises Approach to Scoliosis (SEAS) approach, improved the largest curve by 0.33°, and the sum of curves by 0.67°, while in the “usual” rehabilitation programme, the largest curve worsened by 1.12° and the sum of curves by 1.38°. This study also provided a low quality of evidence in favour of PSSE compared to general exercises in avoiding brace prescription [272].

Most recently, Anwer et al. conducted a systematic review to evaluate the effects of the exercises on spinal deformities and quality of life in patients with adolescent idiopathic scoliosis [284]. They included randomized and non-randomized clinical trials that compared the effect of exercises with other interventions or controls on Cobb angle, body surface measurements, and quality of life (QOL). Of nine studies that met the inclusion criteria, four were RCTs [88, 285–287], four prospective non-randomized controlled [250, 275, 276, 288] and one retrospective controlled trial [289]. The review concluded that moderate quality of evidence supports using the exercise treatment for reducing the Cobb angle, angle of trunk rotation, thoracic kyphosis angle and lumbar lordosis angle, as well as improving the quality of life in patients with AIS. Low quality of evidence supported using the exercises for reducing average lateral deviation.

Of the four RCTs included in this systematic review, two were investigating the effect of PSSE [88, 285]. One investigated forward head correction exercises in combination with standard exercises consisting of stretching of the muscles on the concave and strengthening of the muscles on the convex side of the body [286] and the other RCT tested the effect of the Schroth intensive inpatient PSSE combined with passive transverse forces treatment as compared to the Schroth intensive inpatient PSSE alone [287]. The latter one, in fact, does not fit the inclusion criteria, because passive transverse forces are not an exercise treatment.

Monticone et al.’s RCT presents the first strong evidence supporting the use of PSSE in adolescents with idiopathic scoliosis [88]. The sample included girls with mean age of 12.5 ± 1.1 years, Cobb angle of 19.3° ± 3.9°, and Risser of 0.55. The study found that scoliosis-specific active self-correction and task-oriented exercises, consistent with SEAS approach, improved Cobb angles by 5.3° at skeletal maturity and that traditional exercises were associated with stable curves [290]. One year after the end of the study, the patients’ curves remained stable.

Another, recent RCT conducted by Kuru et al. investigated the effect of supervised Schroth PSSE compared to home-based Schroth PSSE and no treatment, on the change in the Cobb angle, trunk rotation, height of the rib hump, waist asymmetry and SRS-23 domains in patients with AIS. Each group consisted of 15 patients (total of 45 patients) with and average age of 12.9 years, and Cobb of 31.3°. After the 6-month long treatment, the Cobb angle in the supervised Schroth PSSE group improved by 2.5° and deteriorated by 3.3° and 3.1° in the home exercise and control groups, respectively. The supervised Schroth intervention was also superior in improving all other measured outcomes [285].

Another RCT investigated the effect of 12-week long PSSE consistent with Global postural re-education (GPR) intervention on the Cobb angle in patients with thoracic functional scoliosis [291]. In a group of school children with a mean age of 10 years, and the curves ranging from 10° to 20°, the authors reported a significant decrease in the Cobb angle following the treatment (− 5.3°), while the controls, who were not treated, deteriorated by about 1.4°.

Diab et al. compared the effect of forward head corrective exercise treatment added to the traditional exercise treatment including stretching exercises for tight and strengthening exercises for weak muscles to the traditional treatment alone in 76 patients with AIS. The mean age was 13.9 years, and the curves ranged from 10° to 30°. The results demonstrated superiority of the forward head corrective exercises on forward head angle and three-dimensional postural parameters (trunk inclination, lateral deviation, trunk imbalance, thoracic kyphosis, surface rotation, and pelvic torsion and increase in craniovertebral angle and lumbar lordosis) after the 10-week long trial. The benefit of the experimental treatment was maintained after 3 months of follow-up [286].

Zapata et al. published an RCT comparing an 8-week long supervised spinal stabilization exercises programme delivered weekly with a home programme in 34 adolescents with idiopathic scoliosis presenting with pain [292]. After the treatment, the pain measured using Numeric Pain Rating Scale and function measured using the Patient-Specific Functional Scale improved significantly more in the supervised exercise group compared to the unsupervised group.

A prospective quasi-experimental study by Choi et al. examined 44 adolescents with idiopathic scoliosis to compare the effect of 6-week-long supervised (*n* = 28) and non-supervised (*n* = 16) posture management programme based on the Theory of Planned Behaviour in adolescents with mild idiopathic scoliosis [293]. The participants included girls with mean age of 13.2, and Cobb angle of 14.5°, and having had menarche on average for more than a year. The Theory of Planned Behaviour management plan included the practice of continuous posture control behaviours by reinforcing the attitudes and purposes of these behaviours. The posture management programme included exercises to increase the flexibility and strength of muscles around the spine, as well as teaching the correct activities of daily living. Despite the non-randomized study design, the baseline characteristics were similar between the groups even though the dropout rate was higher in the supervised (*n* = 8) than in the control group (*n* = 1). Immediately after the 6-week treatment, the intervention group improved posture management behavioural determinants, flexibility, and muscle strength compared to the controls. Two weeks following the treatment (8 weeks after baseline), it was found that the Cobb angles also improved by 1.67° ± 1.36° in the test group, while it deteriorated by 0.56° ± 0.78° in the control group, and this difference of 2.23° was statistically significant.

A recent methodologically very weak observational study with a control group by Farzaneh et al. compared the effect of 12-week Schroth programme with no treatment in patients with AIS [294]. The authors found that Schroth PSSE decreased the scoliometer measures and the inferior angle of the scapula, and concluded that the Schroth PSSE can “effectively improve the biomechanical and postural parameters.” However, the baseline characteristics in terms of age and Cobb angles were not mentioned, which makes it hard to draw any conclusions.

In the orthopaedic literature [266], a belief that exercises are not useful for scoliosis treatment continues to prevail. This opinion is widespread [57, 295, 296] and presumably comes from an observational study from 1979 (*N* = 99), which showed no difference between exercise and control groups after 1-year follow-up [273]. However, of 42 patients who underwent the exercise treatment, only four reported to have done exercises “daily or almost daily”. This trend of not accepting exercises as a treatment for scoliosis seems to be changing as a consequence of strong emerging evidence. A recent survey of the attitudes of members of the Scoliosis Research Society (SRS) towards PSSE showed that 88% support funding PSSE research and 22% prescribe PSSE [297]. Over the last years, the use of PSSE has increased, especially in North America, due to the interest of patients and families.

The exercises publications have been tentatively classified according to the auto-correction proposed [272]: extrinsic (maximal correction obtained also with the help of gravity, positioning devices and/or limbs placement) [88, 190, 192, 269, 277–280, 282, 283], intrinsic (maximal correction achievable without any external aids) [88, 272, 286, 291, 298–300], no auto-correction but asymmetric exercises [267, 274, 281], and no auto-correction and symmetric exercises [273, 276, 292, 301]. Physiotherapeutic scoliosis-specific exercise schools with some published evidence of efficacy (in alphabetical order) include FITS and DoboMed [277, 291], Global postural re-education [272], Lyon [295–297], MedX [255, 276], Schroth (either as Scoliosis Intensive Rehabilitation [192, 279, 282], or outpatient approach [190, 269, 285]), SEAS [272, 275], and side-shift [278, 280, 283]. However, the natural history of progression of scoliosis is still vastly unknown [48, 302]. It has been widely accepted that the probability of curve deterioration depends on patient age at diagnosis, type and severity of curve, sex and skeletal maturity [46, 55, 303]. However, not all scolioses do progress. Literature suggests that 25 to 75% of diagnosed scoliosis curves remain unchanged, whereas 3 to 12% of curves spontaneously improve [26, 48]. Treatment decisions should be individualized, considering the probability of curve progression, based on curve magnitude, skeletal maturity, patient age and sexual maturity [11, 56].

Finally, treatment acceptability should also be considered. A cross-sectional study recruited families of children who were not affected by scoliosis, but were at the age of risk of AIS onset and 25% of risk of progression. The study found that 87% of participating families supported therapeutic exercises, in comparison to waiting until the curve progresses to a range when bracing would be prescribed [304].

Since the last update of the guidelines, five new RCTs have been published: three new RCTs investigated the effect of PSSE, one symmetric and one asymmetric exercise treatment without auto-correction. The strong Level I evidence supporting the use of PSSE for adolescents with idiopathic scoliosis is rapidly emerging.

To the best of our knowledge, there are three more RCTs underway: (1) the UK trial Active Treatment for Idiopathic Adolescent Scoliosis (ACTIvATeS), trial registry identifier ISRCTN90480705, (2) the Swedish trial CONTRAIS: CONservative TReatment for Adolescent Idiopathic Scoliosis: a randomized controlled trial (NCT01761305), (3) the Canadian multicentre trial: Multicentre Schroth Exercise Trial for Scoliosis – MultiSETS (NCT01610908) and (4) the US multicentre trial Scoliosis-Specific Exercises for At-Risk AIS Curves (NCT02807545).

#### Recommendations on “physiotherapeutic scoliosis-specific exercises to prevent scoliosis progression during growth”

All the recommendation on physiotherapeutic scoliosis-specific exercises to prevent scoliosis progression during growth are summarized in Table 11.

**Table 11:** Recommendation on physiotherapeutic scoliosis-specific exercises to prevent scoliosis progression during growth

Recommendation	Strength	LoE	References
1. Physiotherapeutic scoliosis-specific exercises are recommended as the first step to treat idiopathic scoliosis to prevent/limit progression of the deformity and bracing	C	I	[88, 256, 257, 259, 260, 273, 286, 291, 487]
2. It is recommended that physiotherapeutic scoliosis-specific exercises follow SOSORT Consensus and are based on auto-correction in 3D, training in ADL, stabilizing the corrected posture, and patient education	B	II	[88]
3. It is recommended that physiotherapeutic scoliosis-specific exercises follow one of the Schools that have shown the effectiveness of their approach with scientific studies	C	III	[236–238, 241, 267, 269, 272, 275, 277–283, 489]
4. It is recommended that physiotherapeutic-scoliosis specific exercise programmes are designed by therapists specifically trained in the approach they use	B	V	
5. It is recommended that physiotherapeutic scoliosis-specific exercises are proposed by therapists included in scoliosis treatment teams, with close cooperation between all members	C	V	[88]
6. It is recommended that physiotherapeutic scoliosis-specific exercises are individualized according to patient needs, curve pattern, and treatment phase	B	V	[267, 269, 272, 275, 277–283, 489]
7. It is recommended that physiotherapeutic scoliosis-specific exercises are always individualized even if performed in small groups	B	VI	
8. It is recommended that physiotherapeutic scoliosis-specific exercises are performed regularly throughout treatment to achieve best results	B	V	
9. It is recommended that therapists implement a compliance system for exercise tracking	C	V	
10. It is recommended that therapists regularly assess patients’ quality of physiotherapeutic scoliosis-specific exercises performed by the patients.	B	V	
11. It is recommended that physiotherapeutic scoliosis-specific exercises difficulty is progressively increased according to patient ability.	B	V	
12. It is recommended that physiotherapeutic scoliosis-specific exercises are taught individually in a 1 to 1 relationship to assure individualized care, while regular performance could also be at home or in little groups	C	V	

### Physiotherapeutic specific exercises during brace treatment and surgical therapy

#### Methods

Using the same search strategy and selection criteria as described at the beginning of this chapter, in addition to 40 publications included in the previous search, for this update, we identified three new RCTs—one investigating the effect of PSSE combined with standard of care and two investigating the effect of aerobic physiotherapy for surgical candidates.

#### Results

Although, originally, PSSE were proposed to be performed as add-on to bracing for most brace designs including Milwaukee [305–307], Boston [308], Lyon [12, 309] and Chêneau braces [310–312], they seem to have been underutilized [313].

Specific PSSE have been associated with different brace designs. For example, side-shift as a complement to Milwaukee [191, 283, 314], Schroth to Chêneau brace [190, 192, 315–317], and SEAS to Sforzesco brace [91, 112, 263].

When compared to a systematic review of cohort studies on bracing that formally excluded all protocols with exercises [189], all studies combining exercises and braced showed positive results [135]: surgery rate dropped from the average of 22% (observed) or 23% (brace treated) [171] to 0–7% in the efficacy analysis [92, 112, 190–192], or 10–14% in the worst case analysis [112, 190]. This was true independently by the brace used: Milwaukee and side-shift [283], Chêneau and Schroth [190, 192], cast or Lyon or Sibilla and SEAS [92, 112, 318].

Most recently, a high quality RCT by Schreiber et al. investigated the effect of 6-month Schroth intervention in combination with standard of care including observation and braces in adolescents with idiopathic scoliosis and curves from 10° to 45°. Of 50 patients, 34 wore a brace (17 in each of the groups), mean age was 13.4 ± 1.6 years, and mean Cobb angle 28.5° ± 8.8°. The RCT showed that the Schroth intervention was superior compared to the standard of care alone in improving Cobb angles [319], back muscle endurance [320], SRS-22r pain [320] and self-image domains [320]. In the intention-to-treat analysis, on average, the largest Cobb angle decreased by 1.2° in the Schroth and increased by 2.3° in the control group over 6 months, and this difference was statistically significant. When only completers were considered (*n* = 44), this difference was even larger (4.1°) suggesting the importance of compliance with the treatment.

SOSORT also endorses usage of exercises in the post-surgical rehabilitation period [12, 321]. A survey of Scoliosis Research Society members from 2002 showed that formal physical therapy was unlikely to be recommended by members of the society regardless of procedure [322]. However, the new survey of SRS members, published last year, suggests that this trend has changed. Of 67 surveyed members of the society, 25 (37%) recommended physical therapy post-operatively [297].

It has been reported that patients who experience pain 10 or more years after scoliosis surgery can reduce the pain frequency through a multimodal treatment including stabilizing postural and respiratory exercises [323].

Recently, Dos Santos et al. investigated the effect of 4-month-long preoperative aerobic training on QOL measured by Short Form-36 questionnaire in surgical candidates with AIS [324]. The sample included 40 patients, with mean age of 14.1 ± 1.8 years and mean Cobb angle of 64.2° ± 16.6°. The QOL including function, physical health, pain, general health status, vitality, social and emotional aspects and mental health improved in the group undergoing the aerobic physiotherapy training, while in controls remained stable.

Dos Santos et al. also investigated the postsurgical outcomes in 50 patients with AIS using the same protocol in another RCT [319, 320]. They found that post-surgical recovery, evaluated by 6-Minute Walk Test, was significantly better in patients who underwent a 4-month preoperative physical rehabilitation protocol compared to the controls.

In conclusion, level II evidence supports the use of PSSE alone or in conjunction with braces in patients with AIS with curves of less than 45°. Moreover, aerobic physical therapy is indicated in the preoperative period.

#### Recommendations on “physiotherapeutic scoliosis-specific exercises during brace treatment and surgical therapy”

Table 12 shows all the recommendations on physiotherapeutic scoliosis specific exercises during brace treatment and surgical therapy.

**Table 12:** Recommendation on “physiotherapeutic scoliosis-specific exercises during brace treatment and surgical therapy”

Recommendation	Strength	Evidence	References
1. It is recommended that physiotherapeutic scoliosis-specific exercises are performed during brace treatment	B	II	[92, 112, 190, 191, 489, 492]
2. It is recommended that, while treating with physiotherapeutic scoliosis-specific exercises, therapists work to increase compliance of the patient to brace treatment	B	II	[135, 320]
3. It is recommended that spinal mobilization physiotherapeutic scoliosis specific exercises are used in preparation to bracing	C	V	[276, 347]
4. It is recommended that stabilization physiotherapeutic scoliosis-specific Exercises in autocorrection are used during brace weaning period	C	V	[290]
5. It is recommended that physiotherapeutic scoliosis-specific exercises in painful operated patients are used to reduce pain and increase function	C	V	[348]
6. It is recommended that aerobic physiotherapy training be used prior to surgery.	C	II	[493]

### Other conservative treatments

#### Methods

Using the same search strategy and selection criteria as described at the beginning of this chapter, in addition to 7 primary studies included in the previous search, for this update, we found one more RCT that tested the effect of traditional Chinese medicine in AIS.

#### Results

Short-term (several weeks) [325] and medium-term (several months) [326] of mobilization techniques applied as a stand-alone treatment have been shown to have some effect on the scoliosis outcomes. Mobilization, together with stabilization exercises over a medium- [327] and long-term (several years) interventions, have also shown positive influence on spinal curve [328] and chest expansion [329]; a short-term case series has been reported as well [330]. However, there is lack of high quality evidence of manual treatment [331]. To our knowledge, no studies have been published on the therapeutic efficacy of shoe inserts (excluding heel lifts), conventional and homeopathic medicines, or specific dietary regimens for the correction of idiopathic scoliosis in adolescence.

Since the last update of this guideline, an RCT investigating the effect of traditional Chinese combined medicine in comparison with Milwaukee brace therapy was published [332]. The sample included patients with AIS, mean age 9 years, thus including both Juvenile and adolescent forms and Cobb angle 31° (84.5% were girls). Patients were followed for 12 months for the Cobb angle assessment and for at least 24 months for the other outcome measures (muscles strength and respiratory function). The intervention consisted of spinal balance exercises, manual spinal manipulation and acupotomology, an innovative acupuncture technique of percutaneous minimally invasive soft tissue releasing. The controls wore a Milwaukee brace ≤ 22 h/day and breathing exercises to maintain the body’s flexibility. Following the treatment, the Cobb angle significantly decreased in both groups after 12 and 24 months, but more so in the experimental group (51.4 vs. 47.8% and 62.5 vs. 34.7%, respectively). Pulmonary function significantly improved after 12 months in the experimental, but significantly decreased in the control group. The convex/concave electromyogram ratio was significantly lower in the experimental, but increased in the control group. Considering that the inclusion criteria were not in complete agreement with the SRS criteria, and that the results are short term, the present evidence will not be taken into account as a recommendation.

Posadzki’s systematic review found one high-quality RCT showing no evidence to support osteopathic manual therapy as an effective treatment for mild AIS [333].

#### Recommendations on “other conservative treatments”

Recommendation on other conservative treatments are reported in Table 13.

**Table 13:** Recommendation on other conservative treatment

Recommendation	Strength	Evidence	References
1. It is recommended that manual therapy (gentle, short-term mobilization, or releasing soft tissues techniques) is proposed only if associated with stabilization physiotherapeutic scoliosis specific exercises, unless otherwise justified in the opinion of a clinician specialized in conservative treatment of spinal deformities	C	V	[331]
2. It is recommended that correction of real leg length discrepancy, if needed, is decided by a clinician specialized in conservative treatment of spinal deformities	C	V	

### Respiratory function and exercises

#### Methods

Using the same search strategy and selection criteria as described at the beginning of this chapter, in addition to 35 previously included studies, we did not find any new studies.

#### Results

A series of studies mainly in adolescents with scoliosis between 30° and 60° have demonstrated various types of respiratory impairments in patients: abnormal ventilation patterns, mainly restrictive [314, 316, 334]; impaired function of respiratory muscles [317, 335]; restriction [336, 337] and asymmetric motion of the chest wall, with localized alterations [338]; and abnormal patterns of ventilation during exercise [339], similar to that seen in patients with severe chronic obstructive pulmonary disease (COPD) [340]. Respiratory function is affected by spinal deformity characterized by abnormal lateral flexion, [317] vertebral rotation, [341, 342] spinal stiffness [284] and sagittal diameter of the thoracic cage [343].

Exercise capacity appears to be impaired as well [317, 344–346], but it is not correlated with ventilatory limitations or abnormality in lung volumes [317, 347, 348]. In patients with curves of > 40°, exercise capacity seems to be affected by general muscle dysfunction, even if severe pulmonary impairment is not present [334]. In the same study, it has been shown that the lower limb muscle function is the main contributor of exercise intolerance [334].

Weinstein et al. followed up a prospective natural history cohort (*n* = 117 untreated patients with AIS) for 50 years and compared it to 67 age- and gender-matched controls. They found that shortness of breath was only associated with curves of > 80° [33] and that patients with smaller curves were comparable to the controls. Pehrsson et al. [349, 350] showed that cardiorespiratory failure occurs only in cases of severe scoliosis that had its onset in pre-puberty and with a strong tendency of progression, wherein vital capacity was the strongest indicator for possible respiratory failure. A retrospective study that reviewed records of adult patients with infantile-onset scoliosis showed that those whose scoliosis resolved or was stabilized by non-operative means had normal pulmonary function; those who were managed by casting or bracing and underwent surgery after age 10 had acceptable pulmonary function, but those whose deformity necessitated early surgery had recurrence of deformity and diminished respiratory function [94].

All these studies point to the importance of performing general aerobic activities (including sport) and respiratory training to improve exercise capacity and respiratory muscles functioning.

Most PSSE schools use specific breathing technique as an integral part of the exercise treatment to facilitate de-rotation of the spine and correction of the collapsed areas of the trunk. PSSE have been shown to improve breathing function [269]. SOSORT experts recommend the use of respiratory exercises and education [259]. A large cohort study of patients with scoliosis (*N* = 813) showed that after a course of an in-patient Schroth PSSE intervention, vital capacity and chest wall expansion improved [351].

In an observational study that included 40 girls with scoliosis who were wearing a Boston-type brace, 20 girls underwent aerobic training on a cycle-ergometer 30 min/session 4 days/week for 2 months. The groups were comparable for age, curve magnitude and mean period of brace wear. The authors found that aerobic training sustained or improved significantly the parameters of pulmonary function, while they were reduced in the control group with no exercises who wore the same Boston-type brace [352]. In most of the studies, correction and surgical stabilization of the curve lead to only a slight improvement of pulmonary function, with some exceptions.

#### Recommendations on “respiratory function and exercises”

Recommendation on respiratory function and exercises are shown in Table 14.

**Table 14:** Recommendation on respiratory function and exercises

Recommendation	Strength	Evidence	References
1. It is recommended that, when needed, exercises to improve respiratory function are used	B	V	
2. It is recommended during brace treatment to use exercises to improve respiratory function	C	V	[352]
3. It is recommended to use physiotherapeutic scoliosis-specific exercises to train regional respiratory strategies in order to promote the expansion and ventilation of specific lung compartments	C	V	[351]

### Sports activities

#### Methods

The search has been updated using the methodology explained previously in the text, but we did not find any new studies pertaining to the sport activities in AIS. Eleven articles from the previous search informed the guideline on sport activities.

#### Results

It has been suggested that patients with scoliosis should actively take part in sport activities [353]. Psychological and social aspects are shown to be related to the patients’ self-image [354]. It has also been reported that persons with scoliosis who exercise regularly, show higher self-esteem and have better psychological outcomes [353]. Therefore, SOSORT also recommends patients with scoliosis to remain active in sports activities [2], especially since participation does not seem to affect the occurrence or degree of scoliosis [355].

Despite this, sport activities and PSSE have different aims. While PSSE were developed to specifically target scoliosis deformity, postural control and functional impairments [259, 356–358], sport activities have a more general aim targeted at improving overall fitness and wellness.

It seems as though patients with scoliosis are more likely to participate in sports like gymnastics [359, 360]. It is thought that this is because patients with scoliosis tend to have a higher prevalence of joint laxity than the general population making them more flexible [345]. There is a 10-fold higher incidence of scoliosis among rhythmic gymnasts [361], and a delayed menarche and generalized joint laxity are common in this population. Similarly, an increased incidence of scoliosis has been reported in ballet dancers (24%) [362], and a separate etiology for ballet and rhythmic gymnastics than in adolescent idiopathic scoliosis has been hypothesized [363]. However, in a pair of high-level 13.5-year-old female synchronized swimmers who were also monozygotic twins, only one presented with a 32° thoracolumbar curve. Therefore, it has been implied that factors other than genetics and participation in sport activities play an important role in development of scoliosis [364].

For example it was reported that swimming, which has traditionally been recommended as a good sport activity for scoliosis (and even prescribed by some physicians as a treatment), is associated with an increased risk of trunk asymmetries and hyperkyphosis [365]. In addition, in an old study conducted in 1983, Becker screened 336 competitive adolescent swimmers for scoliosis and found prevalence of scoliosis to be 6.9% [366]. This number seems high, but there is no evidence to suggest that swimming is a causative factor of scoliosis. There is a paucity of correlational research in the area of scoliosis and asymmetric sports, traditionally blamed for causing scoliosis. In addition, in a recent cross-sectional study by Zaina et al., tennis was found not to be correlated with spine deformities [367].

Meyer et al. [360] conducted a survey of matched patients with AIS (*n* = 169) and controls (*n* = 100) and found that adolescents with double major curves participated in more sports activities than those with a single major curve. Moreover, the authors found that adolescents with double major curves were more likely to participate in gymnastics as compared to the adolescents with single curves or controls. This discrepancy could be because patients with double major curves exhibit less scoliosis-related biomechanical repercussions, which lead to a better balance control [360]. In a recent survey of the Spinal Deformity Study Group, which included 23 spinal surgeons, it was reported that on average, modern posterior instrumentation is associated with earlier recommendations for return to sports after fusion for AIS. While the majority of surgeons allowed running by 3 months, noncontact and contact sports by 6 months, and collision sports by 12 months, approximately 20% never allowed return to collision sports, regardless of the surgical method used. However, all surveyed surgeons allowed eventual return to contact and noncontact sports regardless of construct type [368].

#### Recommendations on “sports activities”

Recommendation on sports activities are summarized in Table 15.

**Table 15:** Recommendation on sports activities

Recommendation	Strength	Evidence	References
1. It is recommended that sports is not prescribed as a treatment for idiopathic scoliosis	C	III	[355, 359–362, 364–366, 453]
2. It is recommended that general sports activities are performed because of the specific benefits they offer to patients in terms of psychological, neuromotor and general organic well-being	B	V	
3. It is recommended that, during all treatment phases, physical education at school is continued. Based on the severity of the curve and progression of the deformity and the opinion of a clinician specialized in conservative treatment of spinal deformities, restrictions may be placed on practicing certain types of sports activities	B	V	
4. It is recommended that sports activities are continued also during brace treatment because of the physical (aerobic capacity) and psychological benefits these activities provide	B	V	[352]
5. It is recommended that, during brace treatment, contact or highly dynamic sport activities are performed with caution	C	VI	
6. It is recommended that competitive activities that greatly mobilize the spine are avoided in patients with scoliosis at high risk of progression	C	III	[334–338, 355, 365, 367, 414, 453]

## Assessment

SOSORT has published a consensus paper titled “Methodology of evaluation of morphology of the spine and the trunk in idiopathic scoliosis and other spinal deformities - 6th SOSORT consensus paper” [369]: this can serve as reference for specific insights.

Since scoliosis is diagnosed as idiopathic only by exclusion, it is mandatory at the first evaluation to collect family and personal clinical history and perform a full medical and neurological exam [369].

The clinical assessment will guide further the need of radiological examination, to complete the diagnosis at first evaluation and the need of repeated radiographic exams during follow-up visit in patients already in treatment.

### Clinical assessment

The main evaluation test in the clinical examination of patients with scoliosis is the Adam’s forward bending test. A positive result to the test is pathognomic for scoliosis [370]. The test’s positive predictive value varies since it is proportional to the degree of curvature and depends on operator experience [371].

The Scoliometer [372, 373] measures the hump appearing as a consequence of the Adam’s test: it is an evaluation tool that has proven highly useful. The Scoliometer measures the angle of trunk inclination (ATI, or ATR—Angle of Trunk Rotation) and has a high inter-observer reproducibility, which permits the determination of cut-off points above which a radiographic study is indicated. It has a sensitivity of about 100% and a specificity of about 47% when an ATI angle of 5° is chosen. At an ATI angle of 7°, sensitivity drops to 83% but specificity rises to 86% [19, 374, 375].

Coehlo et al. showed that the correlation between the scoliometer measurements and radiograph analyses was good (*r* = 0.7, *p* < 0.05). The sensitivity, specificity, positive and negative predictive values of the ATR used for referral of scoliotic curvatures greater than 10° Cobb were as follows: 87%, 34%, 0.57, and 0.73 for 5° of ATR and 62%, 75%, 0.71, and 0.66 for 7° of ATR. For curvatures greater than 20°, the results were as follows: 100%, 35%, 0.6, and 1.0 and 66%, 66%, 0.66, and 0.66 for sensitivity, specificity, positive and negative predictive values evaluated for 5° and 7° of ATR, respectively.

The level of the intra- and interrater reliability of the angle of trunk rotation measurement by scoliometer was excellent and very good, respectively [376]. Carlson confirmed that angle of trunk inclination (ATI) is an accepted clinical measurement of trunk asymmetry and has good correlations with Cobb angles (in thoracic curves, *r* = 0.711, *P* < 0.004; RAsag (*r* = 0.730, *P* < 0.003; in thoracolumbar curves, *r* = 0.789, *P* < 0.005); RAsag (*r* = 0.771, *P* < 0.006)) [377]. Also Bonagamba et al. revealed that the assessment of ATR using the scoliometer has good to excellent intra-rater reliability. However, interrater reliability is relatively lower, even when the errors from palpation and positioning of the instrument were eliminated [378]. It is worth noting that some studies suggests that although the measurement of ATR made by the scoliometer is characterized by excellent and substantial intra-examiner agreement for the thoracic and lumbar spine, respectively, the interexaminer measurement error shows poor precision for scoliometer measurements limiting its use as an outcome instrument [379]. Currently, a 7° angle of trunk rotation measured by scoliometer can be considered a good cutoff in a surgical setting, whereas when prevention is desired through a good conservative approach, 5° is a better cutoff [3]. For school scoliosis, screening 5° and 7° angles of trunk rotation is a recommended criterion for referral. This is confirmed by one study which screened the prevalence of scoliosis in school children; the value of the angle of trunk rotation ≥ 5° was used to determine the prevalence of scoliosis in the Korean population of school children (584,554 boys and 550,336 girls, aged 10–14 years old). There were 77,910 (6.2%) children (26,824 boys and 51,086 girls) with ATR > 5° and 37,339 of them had positive results with Cobb angles ≥ 10° (positive predictive value, 46.4%) [380].

However, some authors indicate lower positive predictive values and over-referral at these levels [19]. Huang defined the referral rate for radiography at 5.2% for angle of trunk rotation of 5°. By selecting 6°, 7°, 8°, 9° or 10° angles of trunk rotation as criteria for referral, the referral rate became 2.4, 1.4, 0.7, 0.5, or 0.3%, respectively. The prevalence rate for scoliosis equal to or larger than 10°, 20°, 30° or 40° of the Cobb angle was 1.47, 0.21, 0.04 and 0.02%, respectively, by using a 5° angle of trunk rotation as the criterion for radiography. The positive predictive value was 28.3% for scoliosis of 10° or more, 4% for scoliosis of 20° or more, 0.8% for scoliosis of 30° or more, and 0.4% for scoliosis of 40° or more with a 5° angle of trunk rotation as the criterion for referral. Based on these results, the authors concluded that selecting angles of trunk rotation larger than 5° as criteria for referral for radiography, the positive predictive value increased, but positive cases with larger Cobb angles also decreased markedly [19]. Samuelsson suggests to differentiate the ATR level with respect to the analysed part of the spine and suggested a criterion of 7° or more of ATR for thoracic or right convex curves and one of 6 or more, of ATR for thoracolumbar and lumbar or left convex curves. This methodology provides results adequate for the identification of patients with Cobb angles of 25° or more and reduces the need for spinal radiography and follow-up outside the school screening programmes [381].

The most popular tool for ATR evaluation is a Bunnell scoliometer [19, 374, 375]. However, new tools are also currently proposed. Qiao et al. verified the evaluation of ATR by using the scoliogauge set (Smartphone-aided measurement). The study showed that the intra- and interobserver reliability of measurements of angle of trunk rotation performed both by scoliometer and scoliogauge set was excellent (reliability level ranged from 0.943 to 0.964). However, the intra- and interobserver reliability was better in severe curve (> 40°) [382]. Balg also confirmed that the intraobserver and interobserver reliability of the Scoligauge iPhone app, as well as its validity compared with the scoliometer, are excellent. The mean differences (0.4° ± 3.1°) between measurements are small and clinically not significant. Thus, the Scoligauge application may be valid for clinical evaluation even without special adapter [383]. Also, Franko indicated high correlation between measurements of ATR performed with using scoliometer and scoligauge app (from 0.9994 to 0.9996, *P* values < 0.001). Therefore, the scoligauge app may be a convenient novel tool that replicates the function of a standard clinical scoliometer but with a potentially decreased financial cost, thus confirming the potential to increase the distribution of cost-effective scoliosis screening tools to a broad population of medical providers [384].

Measurement of the hump is another instrument that can provide a further parameter of evaluation and differs from the Scoliometer as it measures the height of the difference between curve concavity and convexity [385–387]. A cutoff point of 5 mm has been defined as significant for measuring back hump [388, 389], and the reliability of this measurement has been reported [374, 385].

#### Screening

A key point to be considered in the assessment of idiopathic scoliosis is screening: through an initial general surface measurement, and a subsequent selected clinical expert evaluation to eventually reach a final radiographic exam, the deformity can be detected early and treated to avoid progression. Even if doubts have been raised, screening for idiopathic scoliosis in asymptomatic adolescents is to be recommended [29]. SOSORT has published a consensus paper titled “SOSORT consensus paper: school screening for scoliosis: Where are we today?” [29]: this can serve as reference for specific insights.

Elevated referral rates for repeat scoliosis examination following school scoliosis screenings have led to questions of efficacy. Further controversy exists regarding school nurses screening for scoliosis due to a lack of evidence indicating a decreased need for scoliosis surgery [390]. Recommendations addressing school screening for adolescents with idiopathic scoliosis are contradictory. As the existing recommendations supporting screening are based on moderate quality of the evidence while the recommendations against screening are based on low-quality evidence, the latter recommendations appear to be both unconvincing and methodologically invalid [391]. Critics indicate over-detection, qualification for therapy of insignificant curves, unjustified treatment, and risks of psychological side effects, whereas supporters underline the need for screening and suggest improvements. Screening programmes are legislated, recommended, or not recommended in different American states. British and Canadian screening recommendations do not mention scoliosis; Australian boards recommend against scoliosis screening programmes. Other publications underline the cost-effectiveness and clinical importance of the procedures [370, 392, 393]. It appears that critical opinions often result from implementing such analyses, whereas those supporting the programmes tend to value the importance of expert opinions [394]. Sabirin et al. based on the a review state that screening for scoliosis among school children is recommended only for high-risk group such as girls at 12 years of age [392].

It is important to highlight that the use of trunk forward bending test (Adams test) alone in school scoliosis screening is insufficient due to a high referral rate (odds ratio [OR] = 2.91) and low positive predictive values for curves ≥ 10° (OR = 0.49) and curves ≥ 20° (OR = 0.34) [395]. Therefore, obtaining objective measurements for this test by the use of scoliometer is important.

Various studies considered the critical opinions regarding cost-effectiveness of school screening programmes: Luk analysed the effectiveness of screening programme based on forward Adams bending test and angle of trunk rotation (ATR) measurement. The subjects with ATR between 5° and 14° or signs of adolescent idiopathic scoliosis were assessed by moiré topography regularly. Students with an ATR of 15° or more, 2 or more moiré lines, or significant clinical signs were referred for radiography and had their Cobb angle measured. Of the 115,190 screened students in the cohort, 3228 (2.8%, 95% confidence interval [CI] = 2.7–2.9%) were referred for radiography. At the final follow-up, the positive predictive values were 43.6% (41.8–45.3%) for a Cobb angle ≥ 20° and 9.4% (8.4–10.5%) for needing treatment, while the sensitivities were 88.1% (86.4–89.6%) and 80.0% (75.6–83.9%), respectively. According to authors, the obtained results indicate that screening should thus be continued in order to facilitate early administration of conservative treatments [396].

Lee et al. analysed the costs of the Hong Kong scoliosis screening programme. The total expenses in the screening centers increased steadily from USD 380,930 in 1995/1996 to USD 2,417,824 in 2005/2006. Based on the analysis of 115,190 students, the authors showed that the costs of screening and diagnosing for one student during adolescence were comprised between 17.94 and 2.08 USD. Of the 1311 referrals who attended the specialist hospitals for diagnosis, 264 and 39 had been braced and operated on, respectively. The medical care cost averaged USD 34.61 per student screened. The cost of finding 1 student with a curvature ≥ 20° and 1 treated case were USD 4475.67 and USD 20,768.29 respectively [397].

Ugras et al. after analysis of the screening conducted in Turkey in 4259 children (2057 females and 2022 males aged 10–14 years old) revealed a positive bending test in 39 children. The prevalence of scoliosis was 25 per 1000 in the screened population. A minor curve was detected in 72.7% of children with scoliosis (Cobb angle of 10°–20°), and a major curve was found in 27.3% (Cobb angle > 20°). The cost of screening was found to be 47 cents per child, but the cost per case of scoliosis was determined to be $236.81. Therefore, according to authors, school screening for scoliosis seems to be cost-effective in Turkey [398].

To improve the effectiveness of screening programmes, Leone et al. proposed to use a “two-step” school-based scoliosis screening procedure as it provides reasonable sensitivity and specificity while reducing costs and radiation exposure to children. The first clinical examination was performed by school physicians, and uncertain cases were referred to an orthopaedist (second step). A screening of 10,000 children directly performed by orthopaedists would result in 291 X-ray exams (2.91%). A screening of the same number of children using a two-step procedure would result in 150 X-ray exams (1.5%), with a savings of 4935 euros for the National Health Care System, a reduction of 0.283 Sv of collective radiation dose, and an estimated 50% reduction in the number of radiogenic malignant tumours procedure [399].

### Radiological assessment

Poor literature is published about how often radiographic assessment is necessary for scoliosis diagnosis, evaluation, and follow-up. There is a general agreement to avoid inappropriate use of X-rays in children to reduce the exposure. According to the SOSORT consensus on X-rays exposure published in 2012, children should be X-rayed at first evaluation in both projection, the postero-anterior and the lateral one. Scoliosis experts agreed that x-rays should be performed at the time of first evaluation and then every 6–12 months afterward in an effort to limit the total number of X-rays. Experts also agreed that an in-brace X-ray was appropriate at the time a brace was prescribed. Follow-up radiograph should be taken using the fewest possible projection, thus meaning to avoid the lateral view if not needed [400].

Radiographic examination remains the reference standard for scoliosis diagnosis; the lateral view at start is essential to have an overview of the sagittal profile, to check for sagittally unbalanced spine and pelvis, and to check for other frequently associated deformities like Scheuermann disease and spondylolisthesis [356]. The use of radiographic evaluations to assess brace effectiveness was also discussed during the SOSORT consensus in 2011: a highly variable protocol resulted regarding the timing and the modalities used to verify brace effectiveness. The only agreement reached regarded the recommendation to use X-rays in critical situations, by maintaining a particular attention in reducing exposure by only using postero-anterior projection and by minimizing its use as much as possible. Therefore, despite the known effect of brace on the sagittal parameters of the spine and pelvis [401], the lateral projection of the in brace correction is not considered essential for brace check.

In the lateral view of the spine obtained through lateral X-rays, it is possible to obtain the Cobb angle measurement for thoracic kyphosis and lumbar lordosis, the parameters defining the sagittal global balance of the spine, like the spino-sacral angle, the spino-pelvic angle and also the sagittal vertical axis [400]. In addition, it is possible to measure the parameters which define the pelvis morphology and position: the pelvic incidence angle, the sacral slope and the pelvic tilt [359, 360, 402].

In the lateral X-rays, the need to move the arm from the anatomical position to show the spine influences the magnitude of thoracic kyphosis and lumbar lordosis [388, 403–405], while surface evaluation of the sagittal profile of the trunk are not affected by the position of the upper limbs. This is the reason why surface topography, after having diagnosed the deformity of the spine through x-rays, is considered complementary to radiographic assessment and can substitute it for patients’ follow-up [369, 406, 407].

Recently, the evaluation of the spine in the sagittal plane and the pelvic sagittal parameters has gained an increasing importance, and some relations between the sagittal balance of the spine and pelvis and scoliosis progression were found [408–410]. The awareness of the impact of scoliosis progression on the sagittal balance of the spine and pelvis together with the spread of new technologies providing low dose radiographic examination is allowing the evaluation of scoliosis patients in both projection (AP and LL) even during follow-up visit. This, in the future, will lead to a better understanding of the correlations among scoliosis and sagittal balance, and the possible role of predictors of all sagittal and pelvic parameters.

Vidal et al. revealed that lateral full-spine low-dose EOS radiographs performed in subjects with idiopathic scoliosis showed excellent intra and interobserver reliability in measurements of sagittal curvatures, pelvic parameters and global sagittal balance [411]. Also, Somoskeöy et al. revealed that both conventional manual 2D and sterEOS 3D are comparable and characterized by excellent intraobserver reliability of measurements of sagittal curvatures of the spine in subjects with idiopathic scoliosis and with Scheuermann disease [412]. In addition, the segmental Cobb angle measurement of sagittal curvature exhibited a higher degree of reliability than the vertebral wedge ratio [413].

It is important to use one of the clinical cutoff points mentioned above (ATI or hump), before ordering a radiographic study, and during regular follow-up to reduce the burden of radiations [369]. Cobb angle measurements on the same radiographic image had an intra- and inter-observer variability of 3°–5° and 6°–7°, respectively [414]; this classically reported error increases when the postural and even diurnal changes in different exams are considered [357, 415]. When measured manually on the radiograph, the most commonly cited measurement error of Cobb angle is 5° [58–63]. However, new measurement computer-assisted methods have lesser measurement errors, ranging from 1.22° to 3.6° [64]. When making clinical decisions, the measurement error thresholds of a corresponding method used should be taken into account.

Radiographic measurement of the vertebral rotation using Perdriolle’s torsiometer has been shown to be reproducible [416]. Based on the same principle, use of Raimondi’s tables or ruler makes measurement easier and slightly more reproducible [417].

In infantile idiopathic scoliosis frontal plane radiographs, a very important measurement has been proposed by Mehta: the rib-vertebra angle that provide a prognostic factor allowing the examiner to distinguish between evolving and resolving scoliosis [129, 418, 419].

The Risser sign [420] constitutes a further parameter for radiographic evaluation and is useful in indicating the patient’s growth status, since Risser grading can be done using the same radiographic film as to evaluate the scoliosis [163, 375–377]. Other essential parameters to be considered are radiographic maturity of the ring apophyses (annular apophyses), appearance of menarche in girls, and Tanner staging [369]. Other diagnostic imaging procedures are in use in idiopathic scoliosis, like various radiographic technique beyond classical projections [421], MRI [421, 422], and neurophysiological exams [423]. Nevertheless, beyond their importance in the surgical setting, in the everyday use for conservative purposes, these techniques are not supported by the actual evidence, unless there are symptoms and signs of neurological compromise [424]. Magnetic resonance imaging does not serve for deformity evaluation; however, it should be ordered to rule out the diagnosis of non-idiopathic scoliosis (Chiari malformation, syringomyelia, diastematomyelia, tethered spinal cord). Computed tomography is not used in non-surgical management of idiopathic scoliosis because of high radiation dose [406].

### Surface assessment

#### Aesthetics

Because aesthetics is a major concern for AIS patients [36], a specific assessment of trunk asymmetries should be used. The evaluation of aesthetics can be done through trunk asymmetry scales (TRACE, POTSI and ATSI) [107, 425, 426] or by means of surface topography or photographic evaluations, thus providing objective measures of the aesthetic profile of the trunk of subjects affected by spinal deformities [15–18]. In addition, the possibility to also collect the patients’ self-perception of the aesthetical impact of the deformities should be considered, and validated scales like the Walter-Reed and the TAPS have been proposed [389, 427–429].

##### TRACE

Aesthetics is a main goal of both conservative and surgical treatments in adolescent idiopathic scoliosis (AIS) [34]. One of the tools for such an evaluation may be the TRACE [107]. The TRACE scale has been recently proposed and validated: it is a 12-point scale based on a visual assessment of shoulders, scapulae, waist and hemithorax asymmetries. Intra-rater reliability was fair, with the minimum significant change being three out of 12, while inter-rater reliability was poor with the minimum significant change being four [92]. However, some authors emphasized that the sensitivity of this tool may be not sufficient to verify the efficiency of brace treatment and an Aesthetic index with higher sensitivity may be a more useful tool [21].

TRACE is an inexpensive, accurate and reproducible tool for aesthetic evaluation and may be also applied in therapy settings to assess postural asymmetry. However, the limitation is that TRACE does not allow to asses 3D parameters which are characteristics of AIS [430].

##### POTSI

The POTSI (Posterior Trunk Symmetry Index) was introduced in 2003 to assess asymmetry of the trunk seen from the back [431]. The POTSI is a comprehensive indicator of the trunk asymmetry, characterized by small measurement error (intra- and inter-observer error 5.5 (range 2.7–9.3) and 6.4 (range 3.8–9.3), respectively). Therefore, it may be used for evaluation of aesthetics in scoliosis patients [411]. The index may also be used to evaluate the relationship between the trunk deformities in the coronal plane and self-esteem [432]. POTSI has, however, poorer standardized response mean than the Cobb angle. Therefore, may not be sensitive enough for scoliosis progression evaluation [108].

##### ATSI

It is important to note that scoliotic deformations may also affect the anterior surface of the trunk and this can be noticed more easily by the patient owing to the visual accessibility of the anterior surface using mirrors. A parameter which allows the analysis of the anterior trunk deformation is the ATSI (Anterior Trunk Symmetry Index). The average ATSI value for 50 healthy children was 25.3 ± 10.6. The threshold value norm defined as mean + 2SD for children aged 6–7 years is 46.5. The intra- and interobserver error for ATSI are small at 1.23 and 3.08, respectively [425].

##### Photography

Another possibility to evaluate the aesthetics is 2D photography [105, 433, 434]. Some studies tried to find correlations between the aesthetic profile of the trunk assessed with photography performed with surface markers and the full-length radiographs. Aroeira suggested a mathematical correlation between X-rays curve measurements and the parameters obtained with computerized photogrammetry. The average agreement found in the determination of the apical vertebra, in the comparisons between radiographic evaluation and the dorsal digital photography with surface markers over the spinous process, was 0.92 and 0.82 at the thoracic and lumbar level, respectively [435]. Further validation studies are required to firmly assess the potential of this method as a complementary assessment tool in the follow-up of scoliosis treatment.

The photographic measurements (shoulder height angle, axilla height angle, left right trapezium angle) revealed an excellent intra- and interobserver reliability (ICC > 0.80). Therefore, digital clinical photography may be a reliable method for objective clinical measurement of shoulder balance in patients with idiopathic scoliosis. However, the assessment of the front and back are not equivalent. Additionally, the correlation between clinical and radiological balance is moderate to weak. Therefore, the measurement of shoulder height angle is not an appropriate method to evaluate the effect of treatment on spinal deformity. Consequently, both examinations (photographic and radiological) should be used for shoulder balance evaluation [416].

Fortin et al. [105] also suggests the use of 2D photography to facilitate clinical practice by monitoring trunk posture among persons with IS. A fair-to-good correlation between 2D and radiograph spinal indices (− 0.33 to − 0.80 with Cobb angles, *P* < 0.05) was found [94]. Therefore, the use of 2D photography may contribute in reducing the use of radiographs to monitor scoliosis progression.

Other research aimed to analyse the reliability of the assessment of posture by using photography is available. A good reliability of marker placement was found and photography represents a mean to improve physiotherapy practice by facilitating the analysis of posture abnormalities. It may also serve to monitor treatment effectiveness or change in posture over the time [105, 425, 440].

Fortin et al. [105] also revealed good to excellent correlation between 2D photography and 3D surface topography for shoulder, pelvis, trunk list, and thoracic scoliosis (0.81 > *r* < 0.97; *P* < 0.01). However, it should be noted that the correlation between 2D and 3D was fair to moderate for thoracic kyphosis, lumbar lordosis, and thoracolumbar or lumbar scoliosis (0.30 > *r* < 0.56; *P* < 0.05). Therefore, these methods should not be considered interchangeable for all parameters [105].

#### Surface topography

##### Static surface topography

Apart of the objective aesthetics evaluation, surface topography also aims to decrease the cumulative exposure to X-ray radiation of patients with scoliosis. De Korvin revealed that surface topography enabled the detection of a five° increase in Cobb angle with a sensitivity of 86% and a specificity of 50%. Therefore, the surface topography may reduce the number of X-ray examinations, as it can help in detecting progression of Cobb angle [109]. These finding are confirmed by Komeili et al. who found that 43% of non-progressive cases analysed between two visits by surface topography would not need an X-ray examination. Additionally, the proposed classification model allowed to detect 85.7% of the progression and 71.6% of the non-progression cases. For thoracic and thoraco-lumbar scoliosis, the false-negative rate was 4%. For lumbar scoliosis, 100% of progression cases were detected. However, due to the small number of lumbar scoliosis analysed, the authors suggest the need to conduct further research to confirm this finding [110].

According to Parent et al., the best surface topography parameters allowing to detect idiopathic scoliosis progression are the following: decompensation, trunk rotation, and lordosis angle, respectively [108].

Despite the clinical usefulness of surface topography, it is worth noting that it is unlikely that surface topography will supplant radiography for the ascertainment of Cobb angles, because the error margins of both methods are wide, and the two are not measuring the same aspect of the deformity. However, there is a significant correlation between Cobb angle and Quantec spinal angle. Additionally, a significant change in Cobb angle could be identified by associated change in topographic parameters. Therefore, the surface topography is useful in patient monitoring as an alternative to radiography, without diminishing the standard of care [436].

One of the controversial issues related to surface topography relates to determining the best position to use for patients’ evaluation. De Seze proposed to conduct the surface topography in scoliosis patient in the “folding” positions, that means standing positions with bended shoulders, elbows and wrists so that the dorsal surface of the wrist would be in contact with the chin, while the ulnar sides of the forearms would be in contact with each other up to the elbows. The quality of inter-observer reproducibility for this position is similar to validated radiological positions: “clavicle” position—position with bended elbows, wrists and fingers so as to place the dorsal surface of the 2nd phalanges of the last two fingers in contact with the collarbones, and “straight out” position—patient bring arms forward to place his/her hands on a support in such a way that the forearms are horizontal. However, the “folding” position provides higher thoracic hump values. Therefore, the proposed posture is relevant to explore scoliosis with back surface topography [437].

##### Dynamic surface topography

The development of gait analysis enabled observing trunk motion in gait. However, imaging of the trunk surface was insufficient for the purpose of analysis of spine deformity. In contrast to classical gait analysis laboratory, equipped with a system of video or infrared cameras registering the position of markers placed on the trunk over time, the imaging of dynamic surface topography (DST) is based on optical acquisition of the whole torso surface.

The dynamic surface topography is a rasterstereography based on imaging system designed to evaluate spinal deformity, providing radiation-free imaging of the position, rotation, and shape of the trunk during the gait cycle [438]. The surface topography system calculates reproducible measurements with error ranges comparable to the current standard in dynamic spinal motion analysis (the average standard deviations of same-day repeat measurements were within ± 3° with a range of 0.51° to 2.3°) [418].

One study focused on scoliotic subjects and revealed good correlations between rasterstereographic evaluation and vertebral rotation using the X-ray-based method (Raimondi method) (*r* = 0.52; *P* < 0.0001 for the whole group of scoliotic subjects, and *r* = 0.47;*P* = 0.0001 for subjects with Cobb angle < 30° and *r* = 0.42; *P* < 0.0001 for Cobb angle ≥30°). According to this study, the possibility to use this non-invasive method for deformity assessment in AIS patients is confirmed [439].

Frerich also stated that dynamic surface topography has a test-retest reproducibility comparable to radiography analysis of Cobb angle (ICC = 0.996). Additionally, the correlation between the two measurements was strong, 0.758 for lumbar and 0.872 for thoracic, respectively. Therefore, although this device does not predict curve magnitude exactly (an average difference between dynamic surface topography and radiographic measurements is 9.42° for lumbar and 6.98° for thoracic), the predictions showed a strong correlation. In light of these results, dynamic surface topography can be considered a reliable tool to monitor patients with adolescent idiopathic scoliosis, and it offers the possibility to reduce the need of radiographical examination [440].

From a practical point of view, all the surface topography devices offer the possibility to evaluate the patient in a more physiological position (no need to move upper limbs); this is a clear advantage, as already demonstrated by Zaina and colleagues [441]. An optimal position comparable with normal standing does not exist, and it is not possible to reconstruct in individual patients what the real standing angles would be without moving the arms. According to this study, the arm position is really able to influence the spinal shape, as shown by the absolute differences of angles from the standing position ranged from 4.8° to 13.3° (kyphosis) and from 4.6° to 10.4° (lordosis) [442]. Furthermore, we can also recommend to apply the same position during examination of scoliotic patients with surface topography tools.

#### Other evaluation

##### Sagittal plane evaluation

Sagittal spine balance of the spine and pelvis, in adolescent idiopathic scoliosis (AIS), has become during recent years a very important issue. Global sagittal balance aims to obtain a horizontal gaze and gravity line at the top of the hips when a subject is in a static position, involving proper adjustment of each spine curvature in the sagittal plane [411].

The sagittal profile of the spine is frequently modified in scoliosis patients, and sagittal profile monitoring is advisable when considering all the correlations found between sagittal unbalance and disability or pain in adults with spinal deformities. To monitor the sagittal profile, in clinical practice, many different tools exist, like the plumbline distances, the Inclinometer (s) and the Arcometer [443–445]. The plumb line imbalance and the distance from the apical spinous process of the primary curve to the plumb line may be used for clinical evaluation, for clinical follow-up and also for the physiotherapeutic specific exercises effectiveness evaluation [446], as it is easy, quick, and reliable.

Surface topography measurements that have been widely used for research purposes, and that only recently are becoming used clinically [369, 406, 407], offer the advantages of a sagittal evaluation not influenced by arm positioning. Topalidou et al. [447] proposed to assess the sagittal spinal curvatures with the Spinal Mouse. The authors showed excellent test-retest reliability of measurements performed in the sagittal plane [447]. The sagittal curvatures of the spine may also be evaluated through the digital Saunders inclinometer: the assessment of sagittal spinal curvatures by one investigator provided good repeatability and reliability of measurements (ICC was 0.9 > *α* ≥ 0.8). Measurements performed by more than one investigator displayed lower repeatability. Moreover, the value of the measurement error (ranged 2.8°-3.8°) should be taken into account in the interpretation of results of measurements performed with the Saunders inclinometer [426].

##### Joint laxity/hypermobility (JL)

The clinical evaluation of children with idiopathic scoliosis should be completed with the assessment of JL. The first reason for that is the fact that joint laxity/hypermobility appears more often in girls with idiopathic scoliosis than in healthy controls (23.2% of IS girls and in 13.4% of controls; *P* = 0.02 [448, 449]; and 51.4% of IS girls and 19% of controls; *P* = 0.00015 [422], depending of the adopted cut-off points). However, no relation between JL prevalence and curve size, curve pattern, or scoliosis length exists [421, 422].

Additionally, the children with IS often undergo intensive physiotherapy which use techniques aimed to increase joint mobility and soft tissue flexibility. These techniques may be contraindicated in children with joint laxity [426]. Therefore, before planning exercises, the evaluation of joint laxity by using specific and standardized tests should be performed to guide a more correct choice of exercises [421, 422, 450]. This is important because the evaluation of lumbo-pelvic-hip muscles flexibility is not sufficient, by itself to confirm the joint laxity [451].

Erkula found a relationship between the Beighton scale score and the angle of trunk rotation and suggested to include the evaluation of joint laxity during scoliosis screening. The authors analysed 598 females and 675 males with an average age of 10.4 years and found trunk rotation of 7° or higher in 30 children, who were more lax than the rest of the group and were invited for radiography, with a detection of curves between 11° and 18° in 10 of them [452].

It also worth noticing that some authors suggest a relation between some of types of sport (e.g. dancing, gymnastics) and the risk of scoliosis development [361, 453]. The reason may be the higher rate of joint hypermobility in the dancers [422] or gymnastic groups [359, 361]. It remains if there are some sports which favour joint laxity, or if athletes with a joint laxity are more prone to such sports. The evaluation of joint laxity in routine clinical practice may also be endorsed by the etiologic role in scoliosis the development of a “dangerous triad”, suggested by Tanchev, which include generalized joint laxity, delayed maturity, and asymmetric spinal loading [361].

In clinical practice, many methods for joint laxity evaluation (with various cutoff thresholds) are used, but the most widely applied method is the Beighton score [448, 453, 454] with a cutoff point ≥ 4 out of 9 points for boys and ≥ 5 for girls [455].

##### Physical capacity evaluation

Idiopathic scoliosis affects the musculoskeletal system, but it may also impair the cardiovascular and respiratory systems function [456]. There are some studies which show that children suffering from scoliosis have respiratory dysfunction, including a decreased maximal voluntary ventilation [457]. Czaprowski showed that maximal oxygen intake (l/min) and PWC170 (W; W/kg) values are considerably lower in girls with scoliosis of 25°–40° than in the healthy controls, while no significant differences were observed between girls with mild scoliosis (10°–24°) and the control group [456]. Huh revealed that vital capacity (FVC) and forced expiratory volume in 1 s (FEV1) are significantly inversely correlated with Cobb angle in patients with thoracic-dominant scoliosis [458], thus confirming previous studies [459–461]. The sagittal Cobb angle of the thoracic curve is one of the main factors which influence pulmonary function and physical capacity [456, 458, 460]. Also, the sagittal diameter of the thoracic cage can reduce vital capacity, total lung area and vertebral rotation at the T8 and T9 levels [341]. Moreover, it is also important to consider that brace treatment can also reduce FVC% and FEV1% in thoracic AIS [460].

##### Quality of life assessment (HRQoL)

The impact of spinal deformities on health-related quality of life is very well known, and it was investigated by various authors [101, 462–464].

Scoliosis therapy using bracing can affect the quality of life of patients with scoliosis. Bracing can be a stressful experience and impact patient’s well-being [214, 465]. Surgery was first investigated for its impact on quality of life, and this is why a specific questionnaire was developed to assess the impact of surgical treatment of scoliosis patients [463, 466]. The most frequently used questionnaire to evaluate HRQoL in scoliosis patients is the Scoliosis Research Society-22 (SRS-22) questionnaire [467, 468]. It is a five-domain questionnaire developed according to traditional test theory (CTT) and, in this framework, showed satisfactory properties such as concurrent validity and reliability [469]. One study tested the SRS-22 using item response theory through Rasch analysis and found that the SRS-22 suffers poor metric properties, which eventually prevent properly measuring patients’ HRQoL [470]. As a provisional solution, a Rasch-consistent 7-item questionnaire (SRS-7) was prepared by rearranging single items from the original SRS-22 [470]. Then, Jain et al. showed that SRS-7 is a valid and responsive functional outcome to measure patients with AIS [471] and recently the same version of the questionnaire was tested in a population of adults with spinal deformities and showed to be reliable, responsive and one-dimensional; it was suggested that the SRS-7 can be used as a short alternative to SRS-22 for assessing global changes in patient-reported outcomes over time. The effect of bracing in body image and HRQoL is still controversial; therefore, a comparison between a group of braced patients with a group only under observation was not able to show any negative effect of the treatment on the body image nor on quality of life [472]. In this study, the SAQ questionnaire was used to evaluate body image and the PEDsQoL questionnaire to assess quality of life [473, 474].

On the other hand, specific exercise programmes can improve HRQoL of patients with spinal deformities, as demonstrated by the RCT by Schreiber and colleagues [320].

The impact of spinal deformities in adult and elderly people is completely different compared with growing patients and is more notable for curves exceeding the 30° [50, 51, 102]. The correlation between disability, pain and HRQoL was demonstrated with the classification suggested by Schwab and colleagues [102–104, 475].

The effect of the aesthetic profile of the trunk, while typical of the most severe curves, can influence the HRQoL of all scoliotic patients. However, recently a group of researchers found that the curvature deformation of young women with idiopathic scoliosis, who were treated by means of conservative methods in their development period, did not have an impact on their self-esteem and sexual functioning [432].

In summation, HRQoL issues and disability are other important aspects to be considered in the treatment of patients with scoliosis [34]. A series of instruments (questionnaires) have been proposed to evaluate QoL including the most widely used one the Scoliosis Research Society Questionnaire (SRS29-30 and the SRS-22) [467, 476–478]. Nevertheless, for clinical conservative use, the SRS-22 shows some limits, and other questionnaires have been developed like the Brace Questionnaire (BrQ) [468, 479–481] and the BSSQ (Bad Sobernheim Stress Questionnaire) [462, 468, 482–484]. The current literature points out the increasing need for a questionnaire specifically developed to measure HRQoL in patients treated conservatively, and respecting the following main characteristics: presenting adequate measurement properties and allowing to make comparisons of HRQoL between patients treated differently (with or without brace, exercises, observation).

##### Genetic evaluation

Nevertheless, prudence is advised in using these tools to decide if to treat or not patients: in fact, moving from research, even if performed in wide samples of some hundreds of patients, to the general population requires caution [154–156, 160].

#### Recommendations on “assessment”

Recommendations on assessment are summarized in Table 16.

**Table 16:** Recommendation on assessment

Recommendation	Strength	Evidence	Reference
1. School screening programmes are recommended for the early diagnosis of idiopathic scoliosis	B	IV	[376, 378–380]
2. The schools screening should be performed using the Scoliometer during trunk forward bend (Adam’s test)	B	IV	[376, 378–380]
3. It is recommended that for scoliosis screening programmes 5° and 7° of angle of trunk rotation should be used as criteria for referral	B	V	[376, 378–380]
4. It is recommended that, every time they evaluate children aged from 8 to 15 years, pediatricians, general practitioners and sports physicians perform the Adam’s test for scoliosis screening purposes, using the Scoliometer	B	VI	
5. It is recommended for clinical follow-up to use validated assessment methods and standard clinical data collection forms	B	IV	[376, 378–380]
6. It is recommended to take into account the measurement error for each method applied for the assessment of scoliosis patients	A	IV	[56–62, 369, 371–377, 414]
7. It is recommended to clinically assess in scoliosis patients at least: angle of trunk rotation, aesthetics, and sagittal alignment of the spine. Other possible common evaluations include: pain, respiratory function, =spine and joint flexibility and strength, leg length discrepancy, balance and coordination, quality of life.	B	IV	[396, 397, 462, 468, 482, 484]
8. The sagittal spine balance should be assessed with X-ray	E	III	[164, 400, 408–410]
9. It is recommended that clinical follow-up examinations are performed at least twice a year, a part periods of rapid growth (pubertal spurt, first 3 years of life)	D	IV	[400, 494]
10. It is recommended that frontal radiographic studies are made postero-anteriorly, using digital films with a ratio X-rays, including visualization of the femoral heads and protection of the gonads, in any standing position without the use of support aids or indication of correct posture, unless otherwise justified in the opinion of a clinician specialized in spinal deformities	C	IV	[385, 495]
11. It is recommended that curve magnitude is measured using the Cobb method	C	IV	[62]
12. On radiographic lateral view, the patient’s upper extremities should be placed in a position to uncover the upper thoracic spine. The recommended positions comprise: (1) 45° angle flexion of the arms, elbows extended and hands resting on a support to preserve the sagittal curvature of the spine, (2) the arms crossed over the breasts, (3) the hand resting on the ipsilateral shoulder without pressing it	E	IV	[404, 405]
13. To reduce the invasiveness of follow-up, it is recommended that the least number of projections is made on radiographic studies	C	VI	
14. It is recommended that all idiopathic scoliosis patients, even if not treated, are regularly followed-up	C	VI	

## Conclusions

This is the third edition of the SOSORT guidelines; they represent a further improvement when compared to the previous experiences produced either internationally by SOSORT or nationally by other groups [1–4, 485].

The 2016 SOSORT Guidelines were developed based on the current evidence on CTIS. Over the last 5 years, high-quality evidence has started to emerge, particularly in the areas of efficacy of bracing (one large multicentre trial) and PSSE (three single-centre randomized controlled trials). Several grade A recommendations were presented. Despite the growing high-quality evidence, the heterogeneity of the study protocols limits generalizability of the recommendations.

These updated guidelines have been a big effort of the Committee and the Society to paint the actual situation in this field, starting from the current evidence, and trying to fill at best all the gray areas not covered by the literature, through the well experimented SOSORT Consensus methodology [4, 32, 36, 104, 120, 158, 248, 355, 356].

As always, guidelines offer an overview of the evidence in a specific field and consequently give insights to researchers on which area should be studied more. Looking at tables that resume the final grading of the recommendations in terms of Level of Evidence and Strength of Recommendations respectively, it is possible to understand the already underlined lack of research in general in this specific area [119, 120, 486]: where there was no evidence of strength level I, very few of level II. There is a need for standardization of research methods of conservative treatment effectiveness, as recognized by SOSORT and Scoliosis Research Society (SRS) non-operative management Committee (Additional files 2, 3, 4, 5 and 6).

## Additional files

Additional file 1: Methods and results leading to the final guidelines. (PDF 68 kb)

Additional file 2: Questionnaires used for Dephi procedure, from Round 1 to round 3. (PDF 404 kb)

Additional file 3: Questionnaires used for Dephi procedure, from Round 1 to round 3. (PDF 1130 kb)

Additional file 4: Questionnaires used for Dephi procedure, from Round 1 to round 3. (PDF 179 kb)

Additional file 5: Questionnaires used for Dephi procedure, from Round 1 to round 3. (PDF 2150 kb)

Additional file 6: Questionnaires used for Dephi procedure, from Round 1 to round 3. (PDF 1270 kb)

## Abbreviations

AIS: Adolescent idiopathic scoliosis
FTRB: Full-time rigid brace
HTRB: Half time rigid brace
LoE: Level of evidence
NTRB: Nighttime rigid brace
Obs 12: Observation every 12 months
Obs 3: Observation every 3 months
Obs 36: Observation every 36 months
Obs 6: Observation every 6 months
Obs 8: Observation every 8 months
PAS: Practical approach scheme
PSSE: Physiotherapeutic scoliosis-specific exercises
PTRB: Part-time rigid brace
SIR: Special inpatient rehabilitation
SoE: Strength of evidence
SSB: Scoliosis soft braces
Su: Surgery
TTRB: Total time rigid brace
