# Supplementary material for: 2016 SOSORT guidelines: orthopaedic and rehabilitation treatment of idiopathic scoliosis during growth
Source: Scoliosis Spinal Disord. 2018 Jan 10;13:3. doi: 10.1186/s13013-017-0145-8 (PMC5795289; doi:10.1186/s13013-017-0145-8)
Supplement: Supplementary file 1 — Methods and results leading to the final guidelines. (PDF 68 kb) [file 13013_2017_145_MOESM1_ESM.pdf]

Appendix. Methods and results leading to the final Guidelines

Methodology of Guidelines development

These Guidelines are a revision of the previous 2011 document and followed the same methodology. The review was performed by the members of the Guidelines Committee of SOSORT including Negrini S (chair), Donzelli S (secretary) , Aulisa Angelo Gabriele (vice-chair), Czaprowski D and Schreiber S (members). In a first meeting the methodology was defined by the Committee and approved by SOSORT Board during Katowice SOSORT Meeting.

The previous sections were maintained:

1. Introduction
2. Assessment
3. Specific exercises, respiratory exercises, specific exercises during brace and surgical treatment, sport activities
4. Brace treatment

Each section was completely reviewed by one member of the Committee and approved by all the other members. The following tasks were performed inside the Guidelines Committee:

1. Adjourning of the literature search
2. Proposal for the revision of the literature review
3. Proposal of changes to recommendations and development of the new recommendations
4. Comments on changed proposed

The work inside the Committee concluded with Delphi Round 1 on the Guidelines.

The Delphi Rounds on the Guidelines were 3 in total, and related to:

1. Classifications
2. Aims of treatment
3. Strength of treatments
4. Clinical practice approach table

In a second stage, the SOSORT Board of Directors and Advisory Board have been involved. The members of the Boards were:

- Board of Directors: President: Fabio Zaina (Italy), Past President: Patrick Knott (USA), Elect President: Jacek Durmala (Poland), Secretary: Luke Stikeleather (USA), Treasurer: Helmut Diers (Germany), Editor-in-Chief of "Scoliosis": Theodoros B Grivas (Greece), Members: Nigel Price (USA), Angelo Aulisa (Italy), Cindy Marti (USA)
- Advisory Board: Manuel Rigo, Tomasz Kotwicki, Theodoros B Grivas, Toru Maruyama, Stefano Negrini, Joe O'Brien, Jean Claude de Mauroy

The Boards were required to:

1. correct the new text and proposals
2. review the recommendations
3. propose new recommendations

The Boards were reuqired to perform Delphi Round 2 on the Guidelines (Additional File 2) and Delphi Rounds 1 and 2 on the Recommendations (Additional File 3 and 4). The Delphi Rounds on the Recommendations were 3 in total, and aimed to define for each single recommendation:

1. the level of agreement to define the strength of recommendation and/or reject it
2. the level of evidence if it was not possible to define it according to the literature

The Agreement was set as follows:

Answers	Rating
100%	A - Complete
95-99.9%	B - High
90-94.9%	C - Good
80-89.9%	D - Weak
70-79.9%	E – Very Weak
Below 70%	Absent

The Level of Evidence wa defined as follows:

- I-II-III-IV: according to literature
- V: above 90% Consensus
- VI above 70% Consensus

For the Strength of Recommendation it was defined a cut off of 80%, under which the recommendation was rejected.
In a third stage the SOSORT Members have been involved with Delphi Rounds 3 for Guidelines (Additional File 5) and Recommendations (Additional File 6).
Finally during the Consensus all results have been discussed. Another recommendations was proposed and it was decided to vote on it in a final stage of Consensus and was included in the final Guidelines.
The list of attendees to the Consensus Meeting included: Aristegui Gari G., Belabbassi NA., Berdishevsky H., Berkowitz R., Betts T., Cohen LI., De Mauroy M., Diers H., Dolan LA., Donzelli S, Doucet C., Durmala J., Faught A., Flanagan P., Fortin C., Freedman M., Gage BW., Geldart K., Grimes KE., Halsey M., He X., Hennig S., Hill D., Hinrichs A., Hurst R., Janssen E., Karavidas N., Knott P., Labelle H., Ladell D., Lebel A., Lecante L., Leung A., Lou E., Marcotte P., Marti C., Maruyama T., Matthews M., Maude E., McAviney J., Mendes J., Mendez M., Mendoza A., Monroe MP., Moreau M., Morrison PR., Negrini A., Orthwein PA., Pancholi CV., Parent E., Pearen S., Polly D., Price N., Raso J., Rivett LA., Rodriguez B., Romero D., Romano M., Sbihli A., Schreiber S., Sherratt NA., Silvestre C., Stikeleather L., Sub LJ., Torres B., Van Dijk M., Wang TL., Wood G., Wynne JH., Yamazaki K., Yoon N., Zaina F.

Results on Guidelines

In the following table the charachteristics of responders of the three Delphi Rounds for the Guidelines are presented.

**Delphi Round**	**Population**	**Total**	**Respondents**	**Rate of answer**	**Male**	**Ortho Surg**	**PRM**	**PT**	**PO**	**PhD**	**Others**	**Countries**
**1**	Consensus Commitee	5	5	100%	60%	20%	40%	40%	0	0	0	3
**2**	SOSORT Boards	15	15	100%	93%	33%	33%	6%	6%	6%	13%	9
**3**	SOSORT members	?	91	?	55%	16%	18%	45%	13%	15%	18%	23

At Round 3:

- the Chronological classification was accepted by 100% (SoE: V)
- the Ponseti classification was accepted by 98% (SoE: V)

- of all the other classifications was accepted only Rigo classification, that reached 72% agreement (SoE: VI)

For what the Cobb degrees magnitude classification, this is the overall table of results:

Cobb degrees	<15	16-20	21-25	26-30	31-35	36-40	41-45	46-50	51-55	56-60	>60
Low degree	98%	82%	42%	5%	1%						
Moderate degree	3%	36%	78%	91%	74%	53%	31%	13%	1%		
Severe degree	1%	1%	7%	19%	34%	61%	72%	65%	55%	46%	14%
Very severe degree			1%	2%	4%	9%	24%	43%	55%	71%	95%

The final results could then be resumed as follow (SoE VI)

**Degree of curve**	**Cobb degrees**
**Low**	>20
**Moderate**	21-35
**Moderate to severe**	36-40
**Severe**	41-50
**Severe to very severe**	51-55
**Very severe**	56>

For all the other tables (aims of treatment, strength of treatments and clinical practice appraoch) was reached the minimum of 70% of agreement giving a final SoE VI grade.

Results on Recommendations

In the following table the charachteristics of responders of the three Delphi Rounds for the Recommendations are presented

**Delphi Round**	**Population**	**Total**	**Respondents**	**Rate of answer**	**Male**	**Ortho Surg**	**PRM**	**PT**	**PO**	**PhD**	**Others**	**Countries**
**1**	SOSORT Boards	15	13	87%	92%	31%	38%	7%	7%	7%	7%	9
**2**	SOSORT Boards	15	10	67%	90%	30%	40%	10%	10%	10%	0	8
**3**	SOSORT members	?	61	?	57%	8%	18%	38%	10%	2%	22%	19

The results on Bracing have been:

**Recommendation**	**Agreement**	**LoE**	**SoR**
**1**	A	I	B
**2**	B	V	B
**3**	C	V	B
**4**	C	V	B
**5**	A	I	B
**6**	E	IV	C
**7**	C	V	B
**8**	A	II	B
**9**	C	II	B
**10**	A	V	B
**11**	B	V	B
**12**	A	V	B
**13**	D	VI	C
**14**	C	V	B

**15**	B	V	B
**16**	B	V	B
**17**	A	V	A
**18**	A	V	A
**19**	C	V	B
**20**	B	V	B
**21**	B	V	C
**22**	B	V	A
**23**	D	VI	C
**24**	B	V	B
**25**	A	V	B

The results on PSSE have been:

Recommendation	Agreement	LoE	SoR
**26**	C	I	C
**27**	B	II	B
**28**	C	III	C
**29**	A	V	B
**30**	C	V	C
**31**	B	V	B
**32**	D	VI	B
**33**	A	V	B
**34**	C	V	C
**35**	A	V	B
**36**	B	V	B
**37**	B	V	C

The results on PSSE in bracing and surgery have been:

Recommendation	Agreement	LoE	SoR
**38**	A	II	B
**39**	A	II	B
**40**	C	V	C
**41**	B	V	C
**42**	A	V	C
**43**	C	II	C

The results on Other conservative treatments have been:

Recommendation	Agreement	LoE	SoR
**44**	C	V	C
**45**	C	V	C

The results on Respiratory function and exercises have been:

Recommendation	Agreement	LoE	SoR
**46**	A	V	B
**47**	B	V	C
**48**	C	V	C

The results on Sport activities have been:

Recommendation	Agreement	LoE	SoR
**49**	D	III	C
**50**	A	V	B
**51**	B	V	B
**52**	A	V	B
**53**	D	VI	C
**54**	D	III	C

The results on Assessment have been:

Recommendation	Agreement	LoE	SoR
**55**	A	V	B
**56**	A	V	B
**57**	B	V	B
**58**	B	V	C
**59**	B	V	B
**60**	C	V	B
**61**	B	V	C
**62**	A	V	C
**63**	A	V	A
**64**	B	V	B
**65**	B	V	B
**66**	C	V	B
**67**	C	V	B
**68**	B	V	B
**69**	C	V	B
**70**	C	III	B
**71**	B	V	B
**72**	D	VI	C
**73**	B	V	B
**74**	A	V	A
**75**	C	V	C
**76**	C	V	C
**77**	C	V	B
**78**	C	V	C
**79**	A	V	B
**80**	A	V	A

The overall Strength of Evidence of the approved Recommendations has been:

	I	II	III	IV	V	VI	Total
Bracing	2	2	0	1	18	2	25
Specific exercises to prevent scoliosis progression during growth	1	1	1	0	8	1	12
Specific exercises during brace treatment and surgical therapy	0	3	0	0	3	0	6
Other conservative treatments	0	0	0	0	2	0	2
Respiratory function and exercises	0	0	0	0	3	0	3
Sports activities	0	0	2	0	3	1	6
Assessment	0	0	1	0	24	1	26
Total	3	6	4	1	61	5	80

The overall Strength of the approved Recommendations has been:

	A	B	C	D	Total
Bracing	3	18	4	0	25
Specific exercises to prevent scoliosis progression during growth	0	7	5	0	12
Specific exercises during brace treatment and surgical therapy	0	2	4	0	6
Other conservative treatments	0	0	2	0	2
Respiratory function and exercises	0	1	2	0	3
Sports activities	0	3	3	0	6
Assessment	3	16	7	0	26
Total	6	47	27	0	80
