# Supplementary material for: 2016 SOSORT guidelines: orthopaedic and rehabilitation treatment of idiopathic scoliosis during growth
Source: Scoliosis Spinal Disord. 2018 Jan 10;13:3. doi: 10.1186/s13013-017-0145-8 (PMC5795289; doi:10.1186/s13013-017-0145-8)
Supplement: Supplementary file 2 — Questionnaires used for Dephi procedure, from Round 1 to round 3. (PDF 404 kb) [file 13013_2017_145_MOESM2_ESM.pdf]

Welcome to the Consensus Process for the 2016 SOSORT Guidelines

Dear colleagues

this Consensus process is part of the development of the 2016 SOSORT Guidelines that will adjourne and substitute the 2011 Guidelines (http://scoliosisjournal.biomedcentral.com/articles/10.1186/1748-7161-7-3) and will be published in our official journal Scoliosis and Spinal Deformity.

You will also receive in a second moment the recommendations to be approved.

Please fill in accurately this questionnaire so to make our Guideliens stronger and really representative of our community of expert in conservative orthopedic and rehabilitation scoliosis treatment.

Thank you for participating in our Consensus. Your feedback is important.

Introduction

1. What is your family name ?

2. What is your first name?

3. What is your gender?

- ◯ Female
- ◯ Male

4. What is your age?

- ◯ 18 to 24
- ◯ 25 to 34
- ◯ 35 to 44
- ◯ 45 to 54
- ◯ 55 to 64
- ◯ 65 to 74
- ◯ 75 or older

5. What is profession ?

- ☐ MD, orthopedic surgeon
- ☐ MD, physical and rehabilitation medicine
- ☐ MD, others
- ☐ Physiotherapist
- ☐ Orthotist
- ☐ PhD
- ☐ Other (please specify)

Age classification

6. Can you accept the following classification as clinically relevant for conservative treatment of idiopathic scoliosis during growth (ie: you change your clinical decisions according to this classification) ?

Chronological (age at diagnosis - years.months)
- Infantile 0-2.11
- Juvenile 3-9.11
- Adolescent 10-17.11
- Adult 18-

○ Yes

○ No

○ Other (please specify)

Angular classification

7. With the aim to produce an angular classification of idiopathic scoliosis during growth, how would you define a curve below 15° ?

- [ ] Low degree
- [ ] Moderate degree
- [ ] Severe degree
- [ ] Very severe degree

8. With the aim to produce an angular classification of idiopathic scoliosis during growth, how would you define a curve between 16° and 20° ?

- ○ Low degree
- ○ Moderate degree
- ○ Severe degree
- ○ Very severe degree

9. With the aim to produce an angular classification of idiopathic scoliosis during growth, how would you define a curve between 21° and 25° ?

- ○ Low degree
- ○ Moderate degree
- ○ Severe degree
- ○ Very severe degree

10. With the aim to produce an angular classification of idiopathic scoliosis during growth, how would you define a curve between 26° and 30° ?

- [ ] Low degree
- [ ] Moderate degree
- [ ] Severe degree
- [ ] Very severe degree

11. With the aim to produce an angular classification of idiopathic scoliosis during growth, how would you define a curve between 31° and 35° ?

- ◯ Low degree
- ◯ Moderate degree
- ◯ Severe degree
- ◯ Very severe degree

12. With the aim to produce an angular classification of idiopathic scoliosis during growth, how would you define a curve between 36° and 40° ?

- ☐ Low degree
- ☐ Moderate degree
- ☐ Severe degree
- ☐ Very severe degree

13. With the aim to produce an angular classification of idiopathic scoliosis during growth, how would you define a curve between 41° and 45° ?

- ◯ Low degree
- ◯ Moderate degree
- ◯ Severe degree
- ◯ Very severe degree

14. With the aim to produce an angular classification of idiopathic scoliosis during growth, how would you define a curve between 46° and 50° ?

- ☐ Low degree
- ☐ Moderate degree
- ☐ Severe degree
- ☐ Very severe degree

15. With the aim to produce an angular classification of idiopathic scoliosis during growth, how would you define a curve above 50° ?

- ☐ Low degree
- ☐ Moderate degree
- ☐ Severe degree
- ☐ Very severe degree

Other classifications

16. Can you accept the following classification as clinically relevant for conservative treatment of idiopathic scoliosis during growth (ie: you change your clinical decisions according to this classification) ?

Topographic (Apex)
- Cervical (to disc C6-7)
- Cervico-thoracic (C7 to T1)
- Thoracic (disc T1-2 to disc T11-12)
- Thoraco-lumbar (T12 to L1)
- Lumbar (from disc L1-2)

○ Yes

○ No

○ Other (please specify)

[ ]

17. Which one of the following classifications do you think is clinically relevant for conservative treatment of idiopathic scoliosis during growth ?

(tick the classifications that you think are clinically relevant)

☐ Rigo Classification (clinical + radiological)

☐ Schroth Classification

☐ Lenke classification

☐ King classification

☐ Schwab classification

☐ Aebi classification

☐ Other (please specify)

[ ]

Aims of treatment

18. Tick the main aims of conservative (orthopedic and rehabilitation) scoliosis treatment - ie the aims that you would like to reach independently of the radiograpihc degree of the curve

- [ ] Avoid surgery
- [ ] Improve aesthetics
- [ ] Improve quality of life in adulthood
- [ ] Reduce disability in adulthood
- [ ] Reduce pain in adulthood
- [ ] Avoid cardio-pulmonary complications in adulthood
- [ ] Others (please specify)

19. Tick the primary radiographic aims of conservative (orthopedic and rehabilitation) scoliosis treatment in curves of LOW degree - ie what realistically you would like to reach with your patients

- ○ Straighten the spine (below 10°)
- ○ Remain below 20°
- ○ Remain below 30°
- ○ Remain below 45°
- ○ Others (please specify)

20. Tick the secondary radiographic aims of conservative (orthopedic and rehabilitation) scoliosis treatment in curves of LOW degree - ie what you would like to reach as a second option if the primary aim is not possible due to progression

- ○ Straighten the spine (below 10°)
- ○ Remain below 20°
- ○ Remain below 30°
- ○ Remain below 45°
- ○ Others (please specify)

21. Tick the primary radiographic aims of conservative (orthopedic and rehabilitation) scoliosis treatment in curves of MODERATE degree - ie what realistically you would like to reach with your patients

- Straighten the spine (below 10°)
- Remain below 20°
- Remain below 30°
- Remain below 45°
- Yes with the following changes

22. Tick the secondary radiographic aims of conservative (orthopedic and rehabilitation) scoliosis treatment in curves of MODERATE degree - ie what you would like to reach as a second option if the primary aim is not possible due to progression

- Straighten the spine (below 10°)
- Remain below 20°
- Remain below 30°
- Remain below 45°
- Yes with the following changes

23. Tick the primary radiographic aims of conservative (orthopedic and rehabilitation) scoliosis treatment in curves of SEVERE degree - ie what realistically you would like to reach with your patients

- Straighten the spine (below 10°)
- Remain below 20°
- Remain below 30°
- Remain below 45°
- Yes with the following changes

24. Tick the secondary radiographic aims of conservative (orthopedic and rehabilitation) scoliosis treatment in curves of SEVERE degree - ie what you would like to reach as a second option if the primary aim is not possible due to progression

- ◯ Straighten the spine (below 10°)
- ◯ Remain below 20°
- ◯ Remain below 30°
- ◯ Remain below 45°
- ◯ Others (please specify)

Strength of treatments

We decided to make more simple the table of possible treatments for Idiopathic scoliosis of the 2011 Guidelines. For this puropose we propose to use the same Angular classification of Question 2 to reduce the cathegories. In the meantime we will maintain the minimum and maximum range of possible treatments as previously agreed.

We use here the same hypothetical classification of progression of strength of treatment as proposed in the previous Guidelines according to the Step-by-step Sibilla theory. We introduced the classification of Rigid Bracing timing approved in the SRS-SOSORT Consensus (Night Time, Home Time, Part Time, Full Time, Total Time). This progression of forces can be wrong, and finding an agreement on proved to be impossible in the past. Consequently, we try again here, and we will then eventually adapt the list of treatments. If i twill not be possible to find an agreement, we will maintain this classification as a simple communication tool.

25. Classify in the table below the progression of forces you would consider for the treatments listed above. The actual proposal is reported from 1 (less intensive treatment) to 14 (more intensive treatment). Simply write the numbers from 1 (less intensive treatment) to 14 (more intensive treatment) according to your preference

- [ ] Obs36 - Observation every 36 months
- [ ] Obs12 - Observation every 12 months
- [ ] Obs8 - Observation every 8 months
- [ ] Obs6 - Observation every 6 months
- [ ] Obs3 - Observation every 3 months
- [ ] PSSE - Physiotherapeutic Scoliosis-Specific Exercises
- [ ] SIR - Scoliosis Inpatient Rehabilitation
- [ ] NTRB - Night-Time Rigid Bracing (in bed only)
- [ ] HTRB - Home-Time Rigid Bracing (up to 14 hours/day)
- [ ] SSB - Specific Soft Bracing
- [ ] PTRB - Part-Time Rigid Bracing (15-18 hours/day)
- [ ] FTRB - Full-Time Rigid Bracing (19-22 hours/day)
- [ ] TTRB - Total-Time Rigid Bracing (23-25 hours/day)- Casting
- [ ] Su - Surgery

INFANTILE IDIOPATHIC SCOLIOSIS

26. Tick treatments that you could use in INFANTILE SCOLIOSIS - Low degree

- [ ] Nothing
- [ ] Observation every 36 months
- [ ] Observation every 12 months
- [ ] Observation every 8 months
- [ ] Observation every 6 months
- [ ] Observation every 3 months
- [ ] Physiotherapeutic Scoliosis-Specific Exercises
- [ ] Scoliosis Inpatient Rehabilitation
- [ ] Night-Time Rigid Bracing (in bed only)
- [ ] Home-Time Rigid Bracing (up to 14 hours/day)
- [ ] Specific Soft Bracing
- [ ] Part-Time Rigid Bracing (15-18 hours/day)
- [ ] Full-Time Rigid Bracing (19-22 hours/day)
- [ ] Total-Time Rigid Bracing (23-25 hours/day)- Casting
- [ ] Surgery

27. Tick treatments that you could use in INFANTILE SCOLIOSIS - Moderate degree

- [ ] Nothing
- [ ] Observation every 36 months
- [ ] Observation every 12 months
- [ ] Observation every 8 months
- [ ] Observation every 6 months
- [ ] Observation every 3 months
- [ ] Physiotherapeutic Scoliosis-Specific Exercises
- [ ] Scoliosis Inpatient Rehabilitation
- [ ] Night-Time Rigid Bracing (in bed only)
- [ ] Home-Time Rigid Bracing (up to 14 hours/day)
- [ ] Specific Soft Bracing
- [ ] Part-Time Rigid Bracing (15-18 hours/day)
- [ ] Full-Time Rigid Bracing (19-22 hours/day)
- [ ] Total-Time Rigid Bracing (23-25 hours/day)- Casting
- [ ] Surgery

28. Tick treatments that you could use in INFANTILE SCOLIOSIS - Severe degree

- [ ] Nothing
- [ ] Observation every 36 months
- [ ] Observation every 12 months
- [ ] Observation every 8 months
- [ ] Observation every 6 months
- [ ] Observation every 3 months
- [ ] Physiotherapeutic Scoliosis-Specific Exercises
- [ ] Scoliosis Inpatient Rehabilitation
- [ ] Night-Time Rigid Bracing (in bed only)
- [ ] Home-Time Rigid Bracing (up to 14 hours/day)
- [ ] Specific Soft Bracing
- [ ] Part-Time Rigid Bracing (15-18 hours/day)
- [ ] Full-Time Rigid Bracing (19-22 hours/day)
- [ ] Total-Time Rigid Bracing (23-25 hours/day)- Casting
- [ ] Surgery

JUVENILE IDIOPATHIC SCOLIOSIS

29. Tick treatments that you could use in JUVENILE SCOLIOSIS - Low degree

- [ ] Nothing
- [ ] Observation every 36 months
- [ ] Observation every 12 months
- [ ] Observation every 8 months
- [ ] Observation every 6 months
- [ ] Observation every 3 months
- [ ] Physiotherapeutic Scoliosis-Specific Exercises
- [ ] Scoliosis Inpatient Rehabilitation
- [ ] Night-Time Rigid Bracing (in bed only)
- [ ] Home-Time Rigid Bracing (up to 14 hours/day)
- [ ] Specific Soft Bracing
- [ ] Part-Time Rigid Bracing (15-18 hours/day)
- [ ] Full-Time Rigid Bracing (19-22 hours/day)
- [ ] Total-Time Rigid Bracing (23-25 hours/day)- Casting
- [ ] Surgery

30. Tick treatments that you could use in JUVENILE SCOLIOSIS - Moderate degree

- [ ] Nothing
- [ ] Observation every 36 months
- [ ] Observation every 12 months
- [ ] Observation every 8 months
- [ ] Observation every 6 months
- [ ] Observation every 3 months
- [ ] Physiotherapeutic Scoliosis-Specific Exercises
- [ ] Scoliosis Inpatient Rehabilitation
- [ ] Night-Time Rigid Bracing (in bed only)
- [ ] Home-Time Rigid Bracing (up to 14 hours/day)
- [ ] Specific Soft Bracing
- [ ] Part-Time Rigid Bracing (15-18 hours/day)
- [ ] Full-Time Rigid Bracing (19-22 hours/day)
- [ ] Total-Time Rigid Bracing (23-25 hours/day)- Casting
- [ ] Surgery

31. Tick treatments that you could use in JUVENILE SCOLIOSIS - Severe degree

- [ ] Nothing
- [ ] Observation every 36 months
- [ ] Observation every 12 months
- [ ] Observation every 8 months
- [ ] Observation every 6 months
- [ ] Observation every 3 months
- [ ] Physiotherapeutic Scoliosis-Specific Exercises
- [ ] Scoliosis Inpatient Rehabilitation
- [ ] Night-Time Rigid Bracing (in bed only)
- [ ] Home-Time Rigid Bracing (up to 14 hours/day)
- [ ] Specific Soft Bracing
- [ ] Part-Time Rigid Bracing (15-18 hours/day)
- [ ] Full-Time Rigid Bracing (19-22 hours/day)
- [ ] Total-Time Rigid Bracing (23-25 hours/day)- Casting
- [ ] Surgery

ADOLESCENT IDIOPATHIC SCOLIOSIS - Risser 0

32. Tick treatments that you could use in ADOLESCENT SCOLIOSIS Risser 0 - Low degree

- [ ] Nothing
- [ ] Observation every 36 months
- [ ] Observation every 12 months
- [ ] Observation every 8 months
- [ ] Observation every 6 months
- [ ] Observation every 3 months
- [ ] Physiotherapeutic Scoliosis-Specific Exercises
- [ ] Scoliosis Inpatient Rehabilitation
- [ ] Night-Time Rigid Bracing (in bed only)
- [ ] Home-Time Rigid Bracing (up to 14 hours/day)
- [ ] Specific Soft Bracing
- [ ] Part-Time Rigid Bracing (15-18 hours/day)
- [ ] Full-Time Rigid Bracing (19-22 hours/day)
- [ ] Total-Time Rigid Bracing (23-25 hours/day)- Casting
- [ ] Surgery

33. Tick treatments that you could use in ADOLESCENT SCOLIOSIS Risser 0 - Moderate degree

- [ ] Nothing
- [ ] Observation every 36 months
- [ ] Observation every 12 months
- [ ] Observation every 8 months
- [ ] Observation every 6 months
- [ ] Observation every 3 months
- [ ] Physiotherapeutic Scoliosis-Specific Exercises
- [ ] Scoliosis Inpatient Rehabilitation
- [ ] Night-Time Rigid Bracing (in bed only)
- [ ] Home-Time Rigid Bracing (up to 14 hours/day)
- [ ] Specific Soft Bracing
- [ ] Part-Time Rigid Bracing (15-18 hours/day)
- [ ] Full-Time Rigid Bracing (19-22 hours/day)
- [ ] Total-Time Rigid Bracing (23-25 hours/day)- Casting
- [ ] Surgery

34. Tick treatments that you could use in ADOLESCENT SCOLIOSIS Risser 0 - Severe degree

- [ ] Nothing
- [ ] Observation every 36 months
- [ ] Observation every 12 months
- [ ] Observation every 8 months
- [ ] Observation every 6 months
- [ ] Observation every 3 months
- [ ] Physiotherapeutic Scoliosis-Specific Exercises
- [ ] Scoliosis Inpatient Rehabilitation
- [ ] Night-Time Rigid Bracing (in bed only)
- [ ] Home-Time Rigid Bracing (up to 14 hours/day)
- [ ] Specific Soft Bracing
- [ ] Part-Time Rigid Bracing (15-18 hours/day)
- [ ] Full-Time Rigid Bracing (19-22 hours/day)
- [ ] Total-Time Rigid Bracing (23-25 hours/day)- Casting
- [ ] Surgery

ADOLESCENT IDIOPATHIC SCOLIOSIS - Risser 1

35. Tick treatments that you could use in ADOLESCENT SCOLIOSIS Risser 1 - Low degree

- [ ] Nothing
- [ ] Observation every 36 months
- [ ] Observation every 12 months
- [ ] Observation every 8 months
- [ ] Observation every 6 months
- [ ] Observation every 3 months
- [ ] Physiotherapeutic Scoliosis-Specific Exercises
- [ ] Scoliosis Inpatient Rehabilitation
- [ ] Night-Time Rigid Bracing (in bed only)
- [ ] Home-Time Rigid Bracing (up to 14 hours/day)
- [ ] Specific Soft Bracing
- [ ] Part-Time Rigid Bracing (15-18 hours/day)
- [ ] Full-Time Rigid Bracing (19-22 hours/day)
- [ ] Total-Time Rigid Bracing (23-25 hours/day)- Casting
- [ ] Surgery

36. Tick treatments that you could use in ADOLESCENT SCOLIOSIS Risser 1 - Moderate degree

- [ ] Nothing
- [ ] Observation every 36 months
- [ ] Observation every 12 months
- [ ] Observation every 8 months
- [ ] Observation every 6 months
- [ ] Observation every 3 months
- [ ] Physiotherapeutic Scoliosis-Specific Exercises
- [ ] Scoliosis Inpatient Rehabilitation
- [ ] Night-Time Rigid Bracing (in bed only)
- [ ] Home-Time Rigid Bracing (up to 14 hours/day)
- [ ] Specific Soft Bracing
- [ ] Part-Time Rigid Bracing (15-18 hours/day)
- [ ] Full-Time Rigid Bracing (19-22 hours/day)
- [ ] Total-Time Rigid Bracing (23-25 hours/day)- Casting
- [ ] Surgery

37. Tick treatments that you could use in ADOLESCENT SCOLIOSIS Risser 1 - Severe degree

- [ ] Nothing
- [ ] Observation every 36 months
- [ ] Observation every 12 months
- [ ] Observation every 8 months
- [ ] Observation every 6 months
- [ ] Observation every 3 months
- [ ] Physiotherapeutic Scoliosis-Specific Exercises
- [ ] Scoliosis Inpatient Rehabilitation
- [ ] Night-Time Rigid Bracing (in bed only)
- [ ] Home-Time Rigid Bracing (up to 14 hours/day)
- [ ] Specific Soft Bracing
- [ ] Part-Time Rigid Bracing (15-18 hours/day)
- [ ] Full-Time Rigid Bracing (19-22 hours/day)
- [ ] Total-Time Rigid Bracing (23-25 hours/day)- Casting
- [ ] Surgery

ADOLESCENT IDIOPATHIC SCOLIOSIS - Risser 2

38. Tick treatments that you could use in ADOLESCENT SCOLIOSIS Risser 2 - Low degree

- [ ] Nothing
- [ ] Observation every 36 months
- [ ] Observation every 12 months
- [ ] Observation every 8 months
- [ ] Observation every 6 months
- [ ] Observation every 3 months
- [ ] Physiotherapeutic Scoliosis-Specific Exercises
- [ ] Scoliosis Inpatient Rehabilitation
- [ ] Night-Time Rigid Bracing (in bed only)
- [ ] Home-Time Rigid Bracing (up to 14 hours/day)
- [ ] Specific Soft Bracing
- [ ] Part-Time Rigid Bracing (15-18 hours/day)
- [ ] Full-Time Rigid Bracing (19-22 hours/day)
- [ ] Total-Time Rigid Bracing (23-25 hours/day)- Casting
- [ ] Surgery

39. Tick treatments that you could use in ADOLESCENT SCOLIOSIS Risser 2 - Moderate degree

- [ ] Nothing
- [ ] Observation every 36 months
- [ ] Observation every 12 months
- [ ] Observation every 8 months
- [ ] Observation every 6 months
- [ ] Observation every 3 months
- [ ] Physiotherapeutic Scoliosis-Specific Exercises
- [ ] Scoliosis Inpatient Rehabilitation
- [ ] Night-Time Rigid Bracing (in bed only)
- [ ] Home-Time Rigid Bracing (up to 14 hours/day)
- [ ] Specific Soft Bracing
- [ ] Part-Time Rigid Bracing (15-18 hours/day)
- [ ] Full-Time Rigid Bracing (19-22 hours/day)
- [ ] Total-Time Rigid Bracing (23-25 hours/day)- Casting
- [ ] Surgery

40. Tick treatments that you could use in ADOLESCENT SCOLIOSIS Risser 2 - Severe degree

- [ ] Nothing
- [ ] Observation every 36 months
- [ ] Observation every 12 months
- [ ] Observation every 8 months
- [ ] Observation every 6 months
- [ ] Observation every 3 months
- [ ] Physiotherapeutic Scoliosis-Specific Exercises
- [ ] Scoliosis Inpatient Rehabilitation
- [ ] Night-Time Rigid Bracing (in bed only)
- [ ] Home-Time Rigid Bracing (up to 14 hours/day)
- [ ] Specific Soft Bracing
- [ ] Part-Time Rigid Bracing (15-18 hours/day)
- [ ] Full-Time Rigid Bracing (19-22 hours/day)
- [ ] Total-Time Rigid Bracing (23-25 hours/day)- Casting
- [ ] Surgery

41. Tick treatments that you could use in ADOLESCENT SCOLIOSIS Risser 3 - Low degree

- [ ] Nothing
- [ ] Observation every 36 months
- [ ] Observation every 12 months
- [ ] Observation every 8 months
- [ ] Observation every 6 months
- [ ] Observation every 3 months
- [ ] Physiotherapeutic Scoliosis-Specific Exercises
- [ ] Scoliosis Inpatient Rehabilitation
- [ ] Night-Time Rigid Bracing (in bed only)
- [ ] Home-Time Rigid Bracing (up to 14 hours/day)
- [ ] Specific Soft Bracing
- [ ] Part-Time Rigid Bracing (15-18 hours/day)
- [ ] Full-Time Rigid Bracing (19-22 hours/day)
- [ ] Total-Time Rigid Bracing (23-25 hours/day)- Casting
- [ ] Surgery

42. Tick treatments that you could use in ADOLESCENT SCOLIOSIS Risser 3 - Moderate degree

- [ ] Nothing
- [ ] Observation every 36 months
- [ ] Observation every 12 months
- [ ] Observation every 8 months
- [ ] Observation every 6 months
- [ ] Observation every 3 months
- [ ] Physiotherapeutic Scoliosis-Specific Exercises
- [ ] Scoliosis Inpatient Rehabilitation
- [ ] Night-Time Rigid Bracing (in bed only)
- [ ] Home-Time Rigid Bracing (up to 14 hours/day)
- [ ] Specific Soft Bracing
- [ ] Part-Time Rigid Bracing (15-18 hours/day)
- [ ] Full-Time Rigid Bracing (19-22 hours/day)
- [ ] Total-Time Rigid Bracing (23-25 hours/day)- Casting
- [ ] Surgery

43. Tick treatments that you could use in ADOLESCENT SCOLIOSIS Risser 3 - Severe degree

- [ ] Nothing
- [ ] Observation every 36 months
- [ ] Observation every 12 months
- [ ] Observation every 8 months
- [ ] Observation every 6 months
- [ ] Observation every 3 months
- [ ] Physiotherapeutic Scoliosis-Specific Exercises
- [ ] Scoliosis Inpatient Rehabilitation
- [ ] Night-Time Rigid Bracing (in bed only)
- [ ] Home-Time Rigid Bracing (up to 14 hours/day)
- [ ] Specific Soft Bracing
- [ ] Part-Time Rigid Bracing (15-18 hours/day)
- [ ] Full-Time Rigid Bracing (19-22 hours/day)
- [ ] Total-Time Rigid Bracing (23-25 hours/day)- Casting
- [ ] Surgery

ADOLESCENT IDIOPATHIC SCOLIOSIS - Risser 4

44. Tick treatments that you could use in ADOLESCENT SCOLIOSIS Risser 4 - Low degree

- [ ] Nothing
- [ ] Observation every 36 months
- [ ] Observation every 12 months
- [ ] Observation every 8 months
- [ ] Observation every 6 months
- [ ] Observation every 3 months
- [ ] Physiotherapeutic Scoliosis-Specific Exercises
- [ ] Scoliosis Inpatient Rehabilitation
- [ ] Night-Time Rigid Bracing (in bed only)
- [ ] Home-Time Rigid Bracing (up to 14 hours/day)
- [ ] Specific Soft Bracing
- [ ] Part-Time Rigid Bracing (15-18 hours/day)
- [ ] Full-Time Rigid Bracing (19-22 hours/day)
- [ ] Total-Time Rigid Bracing (23-25 hours/day)- Casting
- [ ] Surgery

45. Tick treatments that you could use in ADOLESCENT SCOLIOSIS Risser 4 - Moderate degree

- [ ] Nothing
- [ ] Observation every 36 months
- [ ] Observation every 12 months
- [ ] Observation every 8 months
- [ ] Observation every 6 months
- [ ] Observation every 3 months
- [ ] Physiotherapeutic Scoliosis-Specific Exercises
- [ ] Scoliosis Inpatient Rehabilitation
- [ ] Night-Time Rigid Bracing (in bed only)
- [ ] Home-Time Rigid Bracing (up to 14 hours/day)
- [ ] Specific Soft Bracing
- [ ] Part-Time Rigid Bracing (15-18 hours/day)
- [ ] Full-Time Rigid Bracing (19-22 hours/day)
- [ ] Total-Time Rigid Bracing (23-25 hours/day)- Casting
- [ ] Surgery

46. Tick treatments that you could use in ADOLESCENT SCOLIOSIS Risser 4 - Severe degree

- [ ] Nothing
- [ ] Observation every 36 months
- [ ] Observation every 12 months
- [ ] Observation every 8 months
- [ ] Observation every 6 months
- [ ] Observation every 3 months
- [ ] Physiotherapeutic Scoliosis-Specific Exercises
- [ ] Scoliosis Inpatient Rehabilitation
- [ ] Night-Time Rigid Bracing (in bed only)
- [ ] Home-Time Rigid Bracing (up to 14 hours/day)
- [ ] Specific Soft Bracing
- [ ] Part-Time Rigid Bracing (15-18 hours/day)
- [ ] Full-Time Rigid Bracing (19-22 hours/day)
- [ ] Total-Time Rigid Bracing (23-25 hours/day)- Casting
- [ ] Surgery

ADULT IDIOPATHIC SCOLIOSIS - up to 25 years of age

47. Tick treatments that you could use in ADULT SCOLIOSIS up to 25 years of age - Low degree

- ☐ Nothing
- ☐ Observation every 36 months
- ☐ Observation every 12 months
- ☐ Observation every 8 months
- ☐ Observation every 6 months
- ☐ Observation every 3 months
- ☐ Physiotherapeutic Scoliosis-Specific Exercises
- ☐ Scoliosis Inpatient Rehabilitation
- ☐ Night-Time Rigid Bracing (in bed only)
- ☐ Home-Time Rigid Bracing (up to 14 hours/day)
- ☐ Specific Soft Bracing
- ☐ Part-Time Rigid Bracing (15-18 hours/day)
- ☐ Full-Time Rigid Bracing (19-22 hours/day)
- ☐ Total-Time Rigid Bracing (23-25 hours/day)- Casting
- ☐ Surgery

48. Tick treatments that you could use in ADULT SCOLIOSIS up to 25 years of age - Moderate degree

- [ ] Nothing
- [ ] Observation every 36 months
- [ ] Observation every 12 months
- [ ] Observation every 8 months
- [ ] Observation every 6 months
- [ ] Observation every 3 months
- [ ] Physiotherapeutic Scoliosis-Specific Exercises
- [ ] Scoliosis Inpatient Rehabilitation
- [ ] Night-Time Rigid Bracing (in bed only)
- [ ] Home-Time Rigid Bracing (up to 14 hours/day)
- [ ] Specific Soft Bracing
- [ ] Part-Time Rigid Bracing (15-18 hours/day)
- [ ] Full-Time Rigid Bracing (19-22 hours/day)
- [ ] Total-Time Rigid Bracing (23-25 hours/day)- Casting
- [ ] Surgery

49. Tick treatments that you could use in ADULT SCOLIOSIS up to 25 years of age - Severe degree

- [ ] Nothing
- [ ] Observation every 36 months
- [ ] Observation every 12 months
- [ ] Observation every 8 months
- [ ] Observation every 6 months
- [ ] Observation every 3 months
- [ ] Physiotherapeutic Scoliosis-Specific Exercises
- [ ] Scoliosis Inpatient Rehabilitation
- [ ] Night-Time Rigid Bracing (in bed only)
- [ ] Home-Time Rigid Bracing (up to 14 hours/day)
- [ ] Specific Soft Bracing
- [ ] Part-Time Rigid Bracing (15-18 hours/day)
- [ ] Full-Time Rigid Bracing (19-22 hours/day)
- [ ] Total-Time Rigid Bracing (23-25 hours/day)- Casting
- [ ] Surgery

ADULT IDIOPATHIC SCOLIOSIS - no pain

50. Tick treatments that you could use in ADULT SCOLIOSIS no pain - Low degree

- [ ] Nothing
- [ ] Observation every 36 months
- [ ] Observation every 12 months
- [ ] Observation every 8 months
- [ ] Observation every 6 months
- [ ] Observation every 3 months
- [ ] Physiotherapeutic Scoliosis-Specific Exercises
- [ ] Scoliosis Inpatient Rehabilitation
- [ ] Night-Time Rigid Bracing (in bed only)
- [ ] Home-Time Rigid Bracing (up to 14 hours/day)
- [ ] Specific Soft Bracing
- [ ] Part-Time Rigid Bracing (15-18 hours/day)
- [ ] Full-Time Rigid Bracing (19-22 hours/day)
- [ ] Total-Time Rigid Bracing (23-25 hours/day)- Casting
- [ ] Surgery

51. Tick treatments that you could use in ADULT SCOLIOSIS no pain - Moderate degree

- [ ] Nothing
- [ ] Observation every 36 months
- [ ] Observation every 12 months
- [ ] Observation every 8 months
- [ ] Observation every 6 months
- [ ] Observation every 3 months
- [ ] Physiotherapeutic Scoliosis-Specific Exercises
- [ ] Scoliosis Inpatient Rehabilitation
- [ ] Night-Time Rigid Bracing (in bed only)
- [ ] Home-Time Rigid Bracing (up to 14 hours/day)
- [ ] Specific Soft Bracing
- [ ] Part-Time Rigid Bracing (15-18 hours/day)
- [ ] Full-Time Rigid Bracing (19-22 hours/day)
- [ ] Total-Time Rigid Bracing (23-25 hours/day)- Casting
- [ ] Surgery

52. Tick treatments that you could use in ADULT SCOLIOSIS no pain - Severe degree

- [ ] Nothing
- [ ] Observation every 36 months
- [ ] Observation every 12 months
- [ ] Observation every 8 months
- [ ] Observation every 6 months
- [ ] Observation every 3 months
- [ ] Physiotherapeutic Scoliosis-Specific Exercises
- [ ] Scoliosis Inpatient Rehabilitation
- [ ] Night-Time Rigid Bracing (in bed only)
- [ ] Home-Time Rigid Bracing (up to 14 hours/day)
- [ ] Specific Soft Bracing
- [ ] Part-Time Rigid Bracing (15-18 hours/day)
- [ ] Full-Time Rigid Bracing (19-22 hours/day)
- [ ] Total-Time Rigid Bracing (23-25 hours/day)- Casting
- [ ] Surgery

ADULT IDIOPATHIC SCOLIOSIS - chronic pain

53. Tick treatments that you could use in ADULT SCOLIOSIS chronic pain - Low degree curvature

- [ ] Nothing
- [ ] Observation every 36 months
- [ ] Observation every 12 months
- [ ] Observation every 8 months
- [ ] Observation every 6 months
- [ ] Observation every 3 months
- [ ] Physiotherapeutic Scoliosis-Specific Exercises
- [ ] Scoliosis Inpatient Rehabilitation
- [ ] Night-Time Rigid Bracing (in bed only)
- [ ] Home-Time Rigid Bracing (up to 14 hours/day)
- [ ] Specific Soft Bracing
- [ ] Part-Time Rigid Bracing (15-18 hours/day)
- [ ] Full-Time Rigid Bracing (19-22 hours/day)
- [ ] Total-Time Rigid Bracing (23-25 hours/day)- Casting
- [ ] Surgery

54. Tick treatments that you could use in ADULT SCOLIOSIS chronic pain - Moderate degree curvature

- [ ] Nothing
- [ ] Observation every 36 months
- [ ] Observation every 12 months
- [ ] Observation every 8 months
- [ ] Observation every 6 months
- [ ] Observation every 3 months
- [ ] Physiotherapeutic Scoliosis-Specific Exercises
- [ ] Scoliosis Inpatient Rehabilitation
- [ ] Night-Time Rigid Bracing (in bed only)
- [ ] Home-Time Rigid Bracing (up to 14 hours/day)
- [ ] Specific Soft Bracing
- [ ] Part-Time Rigid Bracing (15-18 hours/day)
- [ ] Full-Time Rigid Bracing (19-22 hours/day)
- [ ] Total-Time Rigid Bracing (23-25 hours/day)- Casting
- [ ] Surgery

55. Tick treatments that you could use in ADULT SCOLIOSIS chronic pain - Severe degree curvature

- [ ] Nothing
- [ ] Observation every 36 months
- [ ] Observation every 12 months
- [ ] Observation every 8 months
- [ ] Observation every 6 months
- [ ] Observation every 3 months
- [ ] Physiotherapeutic Scoliosis-Specific Exercises
- [ ] Scoliosis Inpatient Rehabilitation
- [ ] Night-Time Rigid Bracing (in bed only)
- [ ] Home-Time Rigid Bracing (up to 14 hours/day)
- [ ] Specific Soft Bracing
- [ ] Part-Time Rigid Bracing (15-18 hours/day)
- [ ] Full-Time Rigid Bracing (19-22 hours/day)
- [ ] Total-Time Rigid Bracing (23-25 hours/day)- Casting
- [ ] Surgery

ELDERLY IDIOPATHIC SCOLIOSIS - no pain

56. Tick treatments that you could use in ELDERLY SCOLIOSIS no pain - Low degree

- [ ] Nothing
- [ ] Observation every 36 months
- [ ] Observation every 12 months
- [ ] Observation every 8 months
- [ ] Observation every 6 months
- [ ] Observation every 3 months
- [ ] Physiotherapeutic Scoliosis-Specific Exercises
- [ ] Scoliosis Inpatient Rehabilitation
- [ ] Night-Time Rigid Bracing (in bed only)
- [ ] Home-Time Rigid Bracing (up to 14 hours/day)
- [ ] Specific Soft Bracing
- [ ] Part-Time Rigid Bracing (15-18 hours/day)
- [ ] Full-Time Rigid Bracing (19-22 hours/day)
- [ ] Total-Time Rigid Bracing (23-25 hours/day)- Casting
- [ ] Surgery

57. Tick treatments that you could use in ELDERLY SCOLIOSIS no pain - Moderate degree

- [ ] Nothing
- [ ] Observation every 36 months
- [ ] Observation every 12 months
- [ ] Observation every 8 months
- [ ] Observation every 6 months
- [ ] Observation every 3 months
- [ ] Physiotherapeutic Scoliosis-Specific Exercises
- [ ] Scoliosis Inpatient Rehabilitation
- [ ] Night-Time Rigid Bracing (in bed only)
- [ ] Home-Time Rigid Bracing (up to 14 hours/day)
- [ ] Specific Soft Bracing
- [ ] Part-Time Rigid Bracing (15-18 hours/day)
- [ ] Full-Time Rigid Bracing (19-22 hours/day)
- [ ] Total-Time Rigid Bracing (23-25 hours/day)- Casting
- [ ] Surgery

58. Tick treatments that you could use in ELDERLY SCOLIOSIS no pain - Severe degree

- [ ] Nothing
- [ ] Observation every 36 months
- [ ] Observation every 12 months
- [ ] Observation every 8 months
- [ ] Observation every 6 months
- [ ] Observation every 3 months
- [ ] Physiotherapeutic Scoliosis-Specific Exercises
- [ ] Scoliosis Inpatient Rehabilitation
- [ ] Night-Time Rigid Bracing (in bed only)
- [ ] Home-Time Rigid Bracing (up to 14 hours/day)
- [ ] Specific Soft Bracing
- [ ] Part-Time Rigid Bracing (15-18 hours/day)
- [ ] Full-Time Rigid Bracing (19-22 hours/day)
- [ ] Total-Time Rigid Bracing (23-25 hours/day)- Casting
- [ ] Surgery

ELDERLY IDIOPATHIC SCOLIOSIS - chronic pain

59. Tick treatments that you could use in ELDERLY SCOLIOSIS chronic pain - Low degree curvature

- [ ] Nothing
- [ ] Observation every 36 months
- [ ] Observation every 12 months
- [ ] Observation every 8 months
- [ ] Observation every 6 months
- [ ] Observation every 3 months
- [ ] Physiotherapeutic Scoliosis-Specific Exercises
- [ ] Scoliosis Inpatient Rehabilitation
- [ ] Night-Time Rigid Bracing (in bed only)
- [ ] Home-Time Rigid Bracing (up to 14 hours/day)
- [ ] Specific Soft Bracing
- [ ] Part-Time Rigid Bracing (15-18 hours/day)
- [ ] Full-Time Rigid Bracing (19-22 hours/day)
- [ ] Total-Time Rigid Bracing (23-25 hours/day)- Casting
- [ ] Surgery

60. Tick treatments that you could use in ELDERLY SCOLIOSIS chronic pain - Moderate degree curvature

- [ ] Nothing
- [ ] Observation every 36 months
- [ ] Observation every 12 months
- [ ] Observation every 8 months
- [ ] Observation every 6 months
- [ ] Observation every 3 months
- [ ] Physiotherapeutic Scoliosis-Specific Exercises
- [ ] Scoliosis Inpatient Rehabilitation
- [ ] Night-Time Rigid Bracing (in bed only)
- [ ] Home-Time Rigid Bracing (up to 14 hours/day)
- [ ] Specific Soft Bracing
- [ ] Part-Time Rigid Bracing (15-18 hours/day)
- [ ] Full-Time Rigid Bracing (19-22 hours/day)
- [ ] Total-Time Rigid Bracing (23-25 hours/day)- Casting
- [ ] Surgery

61. Tick treatments that you could use in ELDERLY SCOLIOSIS chronic pain - Severe degree curvature

- [ ] Nothing
- [ ] Observation every 36 months
- [ ] Observation every 12 months
- [ ] Observation every 8 months
- [ ] Observation every 6 months
- [ ] Observation every 3 months
- [ ] Physiotherapeutic Scoliosis-Specific Exercises
- [ ] Scoliosis Inpatient Rehabilitation
- [ ] Night-Time Rigid Bracing (in bed only)
- [ ] Home-Time Rigid Bracing (up to 14 hours/day)
- [ ] Specific Soft Bracing
- [ ] Part-Time Rigid Bracing (15-18 hours/day)
- [ ] Full-Time Rigid Bracing (19-22 hours/day)
- [ ] Total-Time Rigid Bracing (23-25 hours/day)- Casting
- [ ] Surgery

ELDERLY IDIOPATHIC SCOLIOSIS - trunk decompensation

62. Tick treatments that you could use in ELDERLY SCOLIOSIS trunk decompensation - Low degree curvature

- [ ] Nothing
- [ ] Observation every 36 months
- [ ] Observation every 12 months
- [ ] Observation every 8 months
- [ ] Observation every 6 months
- [ ] Observation every 3 months
- [ ] Physiotherapeutic Scoliosis-Specific Exercises
- [ ] Scoliosis Inpatient Rehabilitation
- [ ] Night-Time Rigid Bracing (in bed only)
- [ ] Home-Time Rigid Bracing (up to 14 hours/day)
- [ ] Specific Soft Bracing
- [ ] Part-Time Rigid Bracing (15-18 hours/day)
- [ ] Full-Time Rigid Bracing (19-22 hours/day)
- [ ] Total-Time Rigid Bracing (23-25 hours/day)- Casting
- [ ] Surgery

63. Tick treatments that you could use in ELDERLY SCOLIOSIS trunk decompensation - Moderate degree curvature

- [ ] Nothing
- [ ] Observation every 36 months
- [ ] Observation every 12 months
- [ ] Observation every 8 months
- [ ] Observation every 6 months
- [ ] Observation every 3 months
- [ ] Physiotherapeutic Scoliosis-Specific Exercises
- [ ] Scoliosis Inpatient Rehabilitation
- [ ] Night-Time Rigid Bracing (in bed only)
- [ ] Home-Time Rigid Bracing (up to 14 hours/day)
- [ ] Specific Soft Bracing
- [ ] Part-Time Rigid Bracing (15-18 hours/day)
- [ ] Full-Time Rigid Bracing (19-22 hours/day)
- [ ] Total-Time Rigid Bracing (23-25 hours/day)- Casting
- [ ] Surgery

64. Tick treatments that you could use in ELDERLY SCOLIOSIS trunk decompensation - Severe degree curvature

- [ ] Nothing
- [ ] Observation every 36 months
- [ ] Observation every 12 months
- [ ] Observation every 8 months
- [ ] Observation every 6 months
- [ ] Observation every 3 months
- [ ] Physiotherapeutic Scoliosis-Specific Exercises
- [ ] Scoliosis Inpatient Rehabilitation
- [ ] Night-Time Rigid Bracing (in bed only)
- [ ] Home-Time Rigid Bracing (up to 14 hours/day)
- [ ] Specific Soft Bracing
- [ ] Part-Time Rigid Bracing (15-18 hours/day)
- [ ] Full-Time Rigid Bracing (19-22 hours/day)
- [ ] Total-Time Rigid Bracing (23-25 hours/day)- Casting
- [ ] Surgery
