# Supplementary material for: 2016 SOSORT guidelines: orthopaedic and rehabilitation treatment of idiopathic scoliosis during growth
Source: Scoliosis Spinal Disord. 2018 Jan 10;13:3. doi: 10.1186/s13013-017-0145-8 (PMC5795289; doi:10.1186/s13013-017-0145-8)
Supplement: Supplementary file 3 — Questionnaires used for Dephi procedure, from Round 1 to round 3. (PDF 1130 kb) [file 13013_2017_145_MOESM3_ESM.pdf]

Welcome to the Consensus on Recommendations for the 2016 SOSORT Guidelines

Dear colleagues

this Consensus process is part of the development of the 2016 SOSORT Guidelines that will adjourne and substitute the 2011 Guidelines (http://scoliosisjournal.biomedcentral.com/articles/10.1186/1748-7161-7-3) and will be published in our official journal Scoliosis and Spinal Deformity.

After the previous Consensus on classifications and treatments, it is now the time to reach a Consensus on the recommendations. You will have to approve or reject the recommendations, and rate their importance. Moreover you can still give suggestions for other recommendations, and or to change and improve the actual version of the recommendations.

Please fill in accurately this questionnaire so to make our Guideliens stronger and really representative of our community of expert in conservative orthopedic and rehabilitation scoliosis treatment.

IMPORTANT NOTE. When there is some evidence on the recommendation, that has been specified using LoE (Level of Evidence) with a number as follow:
- I : Multiple Randomized Controlled Trials or Systematic Reviews of such studies
- II: One Randomized Controlled Trial
- III: Multiple Controlled nonrandomized Studies or Systematic Reviews of such studies
- IV: Other studies
Other two Levels will be defined according to this Consensus results
- V: SOSORT Consensus with more than 90% of agreement
- VI: SOSORT Consensus with 70 to 89% of agreement

When you do not find LoE number, that means that it will be defined after this Consensus.

When there is a literature LoE on a specific recommendation, by definition what can be done is only a rephrasing the recommendation but it cannot be simply rejeted.

Thank you for participating in our Consensus. Your feedback is important.

Bracing

Bracing is recommended to treat adolescent idiopathic scoliosis
LoE: I

1. Do you think this recommendation should be rephrased?

- ◯ No
- ◯ Yes, with the following correction

2. What is the strength of this recommendation ?

- ◯ it must be applied widely and to all patients with this specific need
- ◯ it is important, but can be applied not to all patients with this specific need
- ◯ less important, it can be applied on a voluntary basis
- ◯ very low importance

Bracing

Bracing is recommended to treat juvenile and infantile idiopathic scoliosis as the first step in an attempt to avoid or at least postpone surgery to a more adequate age

3. Do you agree with this recommendation ?

- ◯ No
- ◯ Yes
- ◯ Yes, with the following correction

4. What is the strength of this recommendation ?

- ◯ it must be applied widely and to all patients with this specific need
- ◯ it is important, but can be applied not to all patients with this specific need
- ◯ less important, it can be applied on a voluntary basis
- ◯ very low importance

Bracing

Casting (or very rigid bracing) is recommended to treat infantile idiopathic scoliosis to try stabilizing the curve

5. Do you agree with this recommendation ?

- No
- Yes
- Yes, with the following correction

6. What is the strength of this recommendation ?

- it must be applied widely and to all patients with this specific need
- it is important, but can be applied not to all patients with this specific need
- less important, it can be applied on a voluntary basis
- very low importance

Bracing

It is recommended not to apply bracing to treat patients with curves below 15±5° Cobb, unless otherwise justified in the opinion of a clinician specialized in conservative treatment of spinal deformities

7. Do you agree with this recommendation ?

- No
- Yes
- Yes, with the following correction

8. What is the strength of this recommendation ?

- it must be applied widely and to all patients with this specific need
- it is important, but can be applied not to all patients with this specific need
- less important, it can be applied on a voluntary basis
- very low importance

Bracing

Bracing is recommended to treat patients with curves above 20±5° Cobb, still growing, and demonstrated progression of deformity or elevated risk of worsening, unless otherwise justified in the opinion of a clinician specialized in conservative treatment of spinal deformities
LoE I

9. Do you think this recommendation should be rephrased?

- ◯ No
- ◯ Yes, with the following correction

10. What is the strength of this recommendation ?

- ◯ it must be applied widely and to all patients with this specific need
- ◯ it is important, but can be applied not to all patients with this specific need
- ◯ less important, it can be applied on a voluntary basis
- ◯ very low importance

Bracing

It is recommended that each treating team provide the brace that they know best and are most prepared to manage: due to the actual knowledge, there is no brace that can be recommended over the others

11. Do you agree with this recommendation ?

- ◯ No
- ◯ Yes
- ◯ Yes, with the following correction

12. What is the strength of this recommendation ?

- ◯ it must be applied widely and to all patients with this specific need
- ◯ it is important, but can be applied not to all patients with this specific need
- ◯ less important, it can be applied on a voluntary basis
- ◯ very low importance

Bracing

It is recommended that braces are worn full time or no less than 18 hours per day at the beginning of treatment, unless otherwise justified in the opinion of a clinician specialized in conservative treatment of spinal deformities
LoE: II

13. Do you think this recommendation should be rephrased?

- ◯ No
- ◯ Yes, with the following correction

14. What is the strength of this recommendation ?

- ◯ it must be applied widely and to all patients with this specific need
- ◯ it is important, but can be applied not to all patients with this specific need
- ◯ less important, it can be applied on a voluntary basis
- ◯ very low importance

Bracing

Since there is a "dose-response" to treatment, it is recommended that the hours of bracing per day are in proportion with the severity of deformity, the age of the patient, the stage, aim and overall results of treatment, and the achievable compliance
LoE: II

15. Do you think this recommendation should be rephrased?

- ◯ No
- ◯ Yes, with the following correction

16. What is the strength of this recommendation ?

- ◯ it must be applied widely and to all patients with this specific need
- ◯ it is important, but can be applied not to all patients with this specific need
- ◯ less important, it can be applied on a voluntary basis
- ◯ very low importance

Bracing

It is recommended that braces are worn until the end of vertebral bone growth and then the wearing time is gradually reduced, unless otherwise justified in the opinion of a clinician specialized in conservative treatment of spinal deformities

17. Do you agree with this recommendation ?

- ◯ No
- ◯ Yes
- ◯ Yes, with the following correction

18. What is the strength of this recommendation ?

- ◯ it must be applied widely and to all patients with this specific need
- ◯ it is important, but can be applied not to all patients with this specific need
- ◯ less important, it can be applied on a voluntary basis
- ◯ very low importance

Bracing

It is recommended that the wearing time of the brace is gradually reduced, while performing stabilizing exercises, to allow adaptation of the postural system and maintain results

19. Do you agree with this recommendation ?

- No
- Yes
- Yes, with the following correction

20. What is the strength of this recommendation ?

- it must be applied widely and to all patients with this specific need
- it is important, but can be applied not to all patients with this specific need
- less important, it can be applied on a voluntary basis
- very low importance

Bracing

It is recommended that any mean is used to increase and monitor compliance, including heat sensors, and a careful adherence to the recommendations defined in the SOSORT Guidelines for Bracing Management

21. Do you agree with this recommendation ?

- ◯ No
- ◯ Yes
- ◯ Yes, with the following correction

22. What is the strength of this recommendation ?

- ◯ it must be applied widely and to all patients with this specific need
- ◯ it is important, but can be applied not to all patients with this specific need
- ◯ less important, it can be applied on a voluntary basis
- ◯ very low importance

Bracing

It is recommended that quality of the brace is checked through an in-brace x-ray

23. Do you agree with this recommendation ?

- ◯ No
- ◯ Yes
- ◯ Yes, with the following correction

24. What is the strength of this recommendation ?

- ◯ it must be applied widely and to all patients with this specific need
- ◯ it is important, but can be applied not to all patients with this specific need
- ◯ less important, it can be applied on a voluntary basis
- ◯ very low importance

Bracing

It is recommended that the prescribing physician and the constructing orthotist are experts according to the criteria defined in the SOSORT Guidelines for Bracing Management

25. Do you agree with this recommendation ?

- ◯ No
- ◯ Yes
- ◯ Yes, with the following correction

26. What is the strength of this recommendation ?

- ◯ it must be applied widely and to all patients with this specific need
- ◯ it is important, but can be applied not to all patients with this specific need
- ◯ less important, it can be applied on a voluntary basis
- ◯ very low importance

Bracing

It is recommended that bracing is applied by a well trained therapeutic team, including a physician, an orthotist and a therapist, according to the criteria defined in the SOSORT Guidelines for Bracing Management

27. Do you agree with this recommendation ?

- No
- Yes
- Yes, with the following correction

28. What is the strength of this recommendation ?

- it must be applied widely and to all patients with this specific need
- it is important, but can be applied not to all patients with this specific need
- less important, it can be applied on a voluntary basis
- very low importance

Bracing

It is recommended that all the phases of brace construction (prescription, construction, check, correction, follow-up) are carefully followed for each single brace according to the criteria defined in the SOSORT Guidelines for Bracing Management

29. Do you agree with this recommendation ?

- ◯ No
- ◯ Yes
- ◯ Yes, with the following correction

30. What is the strength of this recommendation ?

- ◯ it must be applied widely and to all patients with this specific need
- ◯ it is important, but can be applied not to all patients with this specific need
- ◯ less important, it can be applied on a voluntary basis
- ◯ very low importance

Bracing

It is recommended that the brace is specifically designed for the type of the curve to be treated

31. Do you agree with this recommendation ?

- No
- Yes
- Yes, with the following correction

32. What is the strength of this recommendation ?

- it must be applied widely and to all patients with this specific need
- it is important, but can be applied not to all patients with this specific need
- less important, it can be applied on a voluntary basis
- very low importance

Bracing

It is recommended that the brace proposed for treating a scoliotic deformity takes into account all the three planes: frontal, horizontal and sagittal

33. Do you agree with this recommendation ?

- ◯ No
- ◯ Yes
- ◯ Yes, with the following correction

34. What is the strength of this recommendation ?

- ◯ it must be applied widely and to all patients with this specific need
- ◯ it is important, but can be applied not to all patients with this specific need
- ◯ less important, it can be applied on a voluntary basis
- ◯ very low importance

Bracing

It is recommended to use the least invasive brace in relation to the clinical situation, provided the same effectiveness, to reduce the psychological impact and to ensure better patient compliance

35. Do you agree with this recommendation ?

- ◯ No
- ◯ Yes
- ◯ Yes, with the following correction

36. What is the strength of this recommendation ?

- ◯ it must be applied widely and to all patients with this specific need
- ◯ it is important, but can be applied not to all patients with this specific need
- ◯ less important, it can be applied on a voluntary basis
- ◯ very low importance

Bracing

It is recommended that braces do not restrict thorax excursion in a way that reduces respiratory function

37. Do you agree with this recommendation ?

- No
- Yes
- Yes, with the following correction

38. What is the strength of this recommendation ?

- it must be applied widely and to all patients with this specific need
- it is important, but can be applied not to all patients with this specific need
- less important, it can be applied on a voluntary basis
- very low importance

Bracing

It is recommended that braces are prescribed, constructed and fitted in an out-patient setting

39. Do you agree with this recommendation ?

- ◯ No
- ◯ Yes
- ◯ Yes, with the following correction

40. What is the strength of this recommendation ?

- ◯ it must be applied widely and to all patients with this specific need
- ◯ it is important, but can be applied not to all patients with this specific need
- ◯ less important, it can be applied on a voluntary basis
- ◯ very low importance

Bracing

41. Do you suggest any other relevant recommendation on bracing ?

42. Do you suggest any other relevant recommendation on bracing ?

Physiotherapeutic Scoliosis Specific Exercises

Physiotherapeutic Scoliosis Specific Exercises are recommended as the first step to treat idiopathic scoliosis to prevent/limit progression of the deformity and bracing
LoE: I

43. Do you think this recommendation should be rephrased?

- No
- Yes, with the following correction

44. What is the strength of this recommendation ?

- it must be applied widely and to all patients with this specific need
- it is important, but can be applied not to all patients with this specific need
- less important, it can be applied on a voluntary basis
- very low importance

Physiotherapeutic Scoliosis Specific Exercises

It is recommended that Physiotherapeutic Scoliosis Specific Exercises follow SOSORT Consensus and are based on auto-correction in 3D, training in ADL, stabilizing the corrected posture, and patient education
LoE: II

45. Do you think this recommendation should be rephrased?

- ◯ No
- ◯ Yes, with the following correction

46. What is the strength of this recommendation ?

- ◯ it must be applied widely and to all patients with this specific need
- ◯ it is important, but can be applied not to all patients with this specific need
- ◯ less important, it can be applied on a voluntary basis
- ◯ very low importance

Physiotherapeutic Scoliosis Specific Exercises

It is recommended that Physiotherapeutic Scoliosis Specific Exercises follow one of the Schools that have shown the effectiveness of their approach with scientific studies
LoE: III

47. Do you think this recommendation should be rephrased?

- ◯ No
- ◯ Yes, with the following correction

48. What is the strength of this recommendation ?

- ◯ it must be applied widely and to all patients with this specific need
- ◯ it is important, but can be applied not to all patients with this specific need
- ◯ less important, it can be applied on a voluntary basis
- ◯ very low importance

Physiotherapeutic Scoliosis Specific Exercises

It is recommended that Physiotherapeutic Scoliosis Specific Exercise programs are designed by therapists specifically trained in the School they use

49. Do you agree with this recommendation ?

- No
- Yes
- Yes, with the following correction

50. What is the strength of this recommendation ?

- it must be applied widely and to all patients with this specific need
- it is important, but can be applied not to all patients with this specific need
- less important, it can be applied on a voluntary basis
- very low importance

Physiotherapeutic Scoliosis Specific Exercises

It is recommended that Physiotherapeutic Scoliosis Specific Exercises are proposed by therapists included in scoliosis treatment teams, with close cooperation between all members

51. Do you agree with this recommendation ?

- No
- Yes
- Yes, with the following correction

52. What is the strength of this recommendation ?

- it must be applied widely and to all patients with this specific need
- it is important, but can be applied not to all patients with this specific need
- less important, it can be applied on a voluntary basis
- very low importance

Physiotherapeutic Scoliosis Specific Exercises

It is recommended that Physiotherapeutic Scoliosis Specific Exercises are individualized according to patients needs, curve pattern, and treatment phase

53. Do you think this recommendation should be rephrased?

- ◯ No
- ◯ Yes, with the following correction

54. What is the strength of this recommendation ?

- ◯ it must be applied widely and to all patients with this specific need
- ◯ it is important, but can be applied not to all patients with this specific need
- ◯ less important, it can be applied on a voluntary basis
- ◯ very low importance

Physiotherapeutic Scoliosis Specific Exercises

It is recommended that Physiotherapeutic Scoliosis Specific Exercises are always individualized even if performed in small groups

55. Do you agree with this recommendation ?

- ◯ No
- ◯ Yes
- ◯ Yes, with the following correction

56. What is the strength of this recommendation ?

- ◯ it must be applied widely and to all patients with this specific need
- ◯ it is important, but can be applied not to all patients with this specific need
- ◯ less important, it can be applied on a voluntary basis
- ◯ very low importance

Physiotherapeutic Scoliosis Specific Exercises

It is recommended that Physiotherapeutic Scoliosis Specific Exercises are performed regularly throughout treatment to achieve best results

57. Do you agree with this recommendation ?

- ◯ No
- ◯ Yes
- ◯ Yes, with the following correction

58. What is the strength of this recommendation ?

- ◯ it must be applied widely and to all patients with this specific need
- ◯ it is important, but can be applied not to all patients with this specific need
- ◯ less important, it can be applied on a voluntary basis
- ◯ very low importance

59. Do you suggest any other relevant recommendation on Physiotherapeutic Scoliosis Specific Exercises ?

60. Do you suggest any other relevant recommendation on Physiotherapeutic Scoliosis Specific Exercises ?

Physiotherapeutic Scoliosis Specific Exercises during brace treatment and surgical therapy

It is recommended that Physiotherapeutic Scoliosis Specific Exercises are performed during brace treatment
LoE: II

61. Do you think this recommendation should be rephrased?

- ◯ No
- ◯ Yes, with the following correction

62. What is the strength of this recommendation ?

- ◯ it must be applied widely and to all patients with this specific need
- ◯ it is important, but can be applied not to all patients with this specific need
- ◯ less important, it can be applied on a voluntary basis
- ◯ very low importance

Physiotherapeutic Scoliosis Specific Exercises during brace treatment and surgical therapy

It is recommended that, while treating with Physiotherapeutic Scoliosis Specific Exercises, therapists work to increase compliance of the patient to brace treatment
LoE: II

63. Do you think this recommendation should be rephrased?

- ◯ No
- ◯ Yes, with the following correction

64. What is the strength of this recommendation ?

- ◯ it must be applied widely and to all patients with this specific need
- ◯ it is important, but can be applied not to all patients with this specific need
- ◯ less important, it can be applied on a voluntary basis
- ◯ very low importance

Physiotherapeutic Scoliosis Specific Exercises during brace treatment and surgical therapy

It is recommended that spinal mobilization Physiotherapeutic Scoliosis Specific Exercises are used in preparation to bracing

65. Do you agree with this recommendation ?

- No
- Yes
- Yes, with the following correction

66. What is the strength of this recommendation ?

- it must be applied widely and to all patients with this specific need
- it is important, but can be applied not to all patients with this specific need
- less important, it can be applied on a voluntary basis
- very low importance

Physiotherapeutic Scoliosis Specific Exercises during brace treatment and surgical therapy

It is recommended that stabilization Physiotherapeutic Scoliosis Specific Exercises in autocorrection are used during brace weaning period

67. Do you agree with this recommendation ?

- ◯ No
- ◯ Yes
- ◯ Yes, with the following correction

68. What is the strength of this recommendation ?

- ◯ it must be applied widely and to all patients with this specific need
- ◯ it is important, but can be applied not to all patients with this specific need
- ◯ less important, it can be applied on a voluntary basis
- ◯ very low importance

Physiotherapeutic Scoliosis Specific Exercises during brace treatment and surgical therapy

It is recommended that Physiotherapeutic Scoliosis Specific Exercises in painful operated patients are used to reduce pain and increase function

69. Do you agree with this recommendation ?

- ◯ No
- ◯ Yes
- ◯ Yes, with the following correction

70. What is the strength of this recommendation ?

- ◯ it must be applied widely and to all patients with this specific need
- ◯ it is important, but can be applied not to all patients with this specific need
- ◯ less important, it can be applied on a voluntary basis
- ◯ very low importance

Physiotherapeutic Scoliosis Specific Exercises during brace treatment and surgical therapy

It is recommended that aerobic physiotherapy training be used prior to surgery.
LoE: II

71. Do you think this recommendation should be rephrased?

- ◯ No
- ◯ Yes, with the following correction

72. What is the strength of this recommendation ?

- ◯ it must be applied widely and to all patients with this specific need
- ◯ it is important, but can be applied not to all patients with this specific need
- ◯ less important, it can be applied on a voluntary basis
- ◯ very low importance

73. Do you suggest any other relevant recommendation on Physiotherapeutic Scoliosis Specific Exercises during brace treatment and surgical therapy ?

74. Do you suggest any other relevant recommendation on Physiotherapeutic Scoliosis Specific Exercises during brace treatment and surgical therapy ?

Other conservative treatments

It is recommended that manual therapy (gentle, short-term mobilization, or releasing soft tissues techniques) is proposed only if associated with stabilization Physiotherapeutic Specific Exercises

75. Do you agree with this recommendation ?

- No
- Yes
- Yes, with the following correction

76. What is the strength of this recommendation ?

- it must be applied widely and to all patients with this specific need
- it is important, but can be applied not to all patients with this specific need
- less important, it can be applied on a voluntary basis
- very low importance

Other conservative treatments

It is recommended that correction of real leg length discrepancy, if needed, is decided by a clinician specialized in conservative treatment of spinal deformities

77. Do you agree with this recommendation ?

- ◯ No
- ◯ Yes
- ◯ Yes, with the following correction

78. What is the strength of this recommendation ?

- ◯ it must be applied widely and to all patients with this specific need
- ◯ it is important, but can be applied not to all patients with this specific need
- ◯ less important, it can be applied on a voluntary basis
- ◯ very low importance

Other conservative treatments

It is recommended that correction of real leg length discrepancy, if needed, is decided by a clinician specialized in conservative treatment of spinal deformities

79. Do you agree with this recommendation ?

- ◯ No
- ◯ Yes
- ◯ Yes, with the following correction

80. What is the strength of this recommendation ?

- ◯ it must be applied widely and to all patients with this specific need
- ◯ it is important, but can be applied not to all patients with this specific need
- ◯ less important, it can be applied on a voluntary basis
- ◯ very low importance

Other conservative treatments

It is recommended that traditional Chinese medicine consisting of spinal balance exercises (Daoyin), manual spinal manipulation (Tuina), and acupotomology be used until the end of growth
LoE: II

81. Do you think this recommendation should be rephrased?

- ◯ No
- ◯ Yes, with the following correction

82. What is the strength of this recommendation ?

- ◯ it must be applied widely and to all patients with this specific need
- ◯ it is important, but can be applied not to all patients with this specific need
- ◯ less important, it can be applied on a voluntary basis
- ◯ very low importance

Other conservative treatments

83. Do you suggest any other relevant recommendation on Other conservative treatments ?

84. Do you suggest any other relevant recommendation on Other conservative treatments ?

Respiratory function and exercises

It is recommended that, when needed, exercises to improve respiratory function are used

85. Do you agree with this recommendation ?

- ◯ No
- ◯ Yes
- ◯ Yes, with the following correction

86. What is the strength of this recommendation ?

- ◯ it must be applied widely and to all patients with this specific need
- ◯ it is important, but can be applied not to all patients with this specific need
- ◯ less important, it can be applied on a voluntary basis
- ◯ very low importance

Respiratory function and exercises

It is recommended during brace treatment to use exercises to improve respiratory function

87. Do you agree with this recommendation ?

- ◯ No
- ◯ Yes
- ◯ Yes, with the following correction

88. What is the strength of this recommendation ?

- ◯ it must be applied widely and to all patients with this specific need
- ◯ it is important, but can be applied not to all patients with this specific need
- ◯ less important, it can be applied on a voluntary basis
- ◯ very low importance

Respiratory function and exercises

It is recommended the use of Physiotherapeutic Scoliosis Specific Exercises to train regional respiratory strategies to promote the expansion and ventilation of specific lung compartments

89. Do you agree with this recommendation ?

- No
- Yes
- Yes, with the following correction

90. What is the strength of this recommendation ?

- it must be applied widely and to all patients with this specific need
- it is important, but can be applied not to all patients with this specific need
- less important, it can be applied on a voluntary basis
- very low importance

Respiratory function and exercises

91. Do you suggest any other relevant recommendation on Respiratory function and exercises ?

92. Do you suggest any other relevant recommendation on Respiratory function and exercises ?

Sport activities

It is recommended that sports is not prescribed as a treatment for idiopathic scoliosis
LoE: III

93. Do you think this recommendation should be rephrased?

- ◯ No
- ◯ Yes, with the following correction

94. What is the strength of this recommendation ?

- ◯ it must be applied widely and to all patients with this specific need
- ◯ it is important, but can be applied not to all patients with this specific need
- ◯ less important, it can be applied on a voluntary basis
- ◯ very low importance

Sport activities

It is recommended that general sports activities are performed because of the specific benefits they offer to patients in terms of psychological, neuromotor and general organic well-being

95. Do you agree with this recommendation ?

- ◯ No
- ◯ Yes
- ◯ Yes, with the following correction

96. What is the strength of this recommendation ?

- ◯ it must be applied widely and to all patients with this specific need
- ◯ it is important, but can be applied not to all patients with this specific need
- ◯ less important, it can be applied on a voluntary basis
- ◯ very low importance

Sport activities

It is recommended that, during all treatment phases, physical education at school is continued. Based on the severity of the curve and progression of the deformity and the opinion of a clinician specialized in conservative treatment of spinal deformities, restrictions may be placed on practicing certain types of sports activities

97. Do you agree with this recommendation ?

- No
- Yes
- Yes, with the following correction

98. What is the strength of this recommendation ?

- it must be applied widely and to all patients with this specific need
- it is important, but can be applied not to all patients with this specific need
- less important, it can be applied on a voluntary basis
- very low importance

Sport activities

It is recommended that sports activities are continued also during brace treatment because of the physical (aerobic capacity) and psychological benefits these activities provide

99. Do you agree with this recommendation ?

- No
- Yes
- Yes, with the following correction

100. What is the strength of this recommendation ?

- it must be applied widely and to all patients with this specific need
- it is important, but can be applied not to all patients with this specific need
- less important, it can be applied on a voluntary basis
- very low importance

Sport activities

It is recommended that, during brace treatment, contact or highly dynamic sport activities are performed with caution

101. Do you agree with this recommendation ?

- ◯ No
- ◯ Yes
- ◯ Yes, with the following correction

102. What is the strength of this recommendation ?

- ◯ it must be applied widely and to all patients with this specific need
- ◯ it is important, but can be applied not to all patients with this specific need
- ◯ less important, it can be applied on a voluntary basis
- ◯ very low importance

Sport activities

It is recommended that competitive activities that greatly mobilize the spine are avoided in patients with scoliosis at high risk of progression
LoE: III

103. Do you think this recommendation should be rephrased?

- No
- Yes, with the following correction

104. What is the strength of this recommendation ?

- it must be applied widely and to all patients with this specific need
- it is important, but can be applied not to all patients with this specific need
- less important, it can be applied on a voluntary basis
- very low importance

Sport activities

105. Do you suggest any other relevant recommendation on sport activities?

106. Do you suggest any other relevant recommendation on sport activities ?

Assessment

School screening programs are recommended for the early diagnosis of idiopathic scoliosis

107. Do you agree with this recommendation ?

- ◯ No
- ◯ Yes
- ◯ Yes, with the following correction

108. What is the strength of this recommendation ?

- ◯ it must be applied widely and to all patients with this specific need
- ◯ it is important, but can be applied not to all patients with this specific need
- ◯ less important, it can be applied on a voluntary basis
- ◯ very low importance

Assessment

The schools screening should be perform with using the Scoliometer during trunk forward bend (Adam`s test)

109. Do you agree with this recommendation ?

- No
- Yes
- Yes, with the following correction

110. What is the strength of this recommendation ?

- it must be applied widely and to all patients with this specific need
- it is important, but can be applied not to all patients with this specific need
- less important, it can be applied on a voluntary basis
- very low importance

Assessment

It is recommended that for scoliosis screening programs 5° and 7° of angle of trunk rotation should be used as criteria for referral

111. Do you agree with this recommendation ?

- ◯ No
- ◯ Yes
- ◯ Yes, with the following correction

112. What is the strength of this recommendation ?

- ◯ it must be applied widely and to all patients with this specific need
- ◯ it is important, but can be applied not to all patients with this specific need
- ◯ less important, it can be applied on a voluntary basis
- ◯ very low importance

Assessment

After the evaluation of the reliability, the evaluation of the angle of trunk rotation may be also performed with using smarthpone aided appropriate application

113. Do you agree with this recommendation ?

- ◯ No
- ◯ Yes
- ◯ Yes, with the following correction

114. What is the strength of this recommendation ?

- ◯ it must be applied widely and to all patients with this specific need
- ◯ it is important, but can be applied not to all patients with this specific need
- ◯ less important, it can be applied on a voluntary basis
- ◯ very low importance

Assessment

It is recommended that the scoliosis school screening should be completed by the joint hypermobility/laxity assessment

115. Do you agree with this recommendation ?

- No
- Yes
- Yes, with the following correction

116. What is the strength of this recommendation ?

- it must be applied widely and to all patients with this specific need
- it is important, but can be applied not to all patients with this specific need
- less important, it can be applied on a voluntary basis
- very low importance

Assessment

It is recommended that the angle of trunk rotation with using of scoliometer or smartphone aided application is spread in the school community and among all people that are engaged in the health of children (parents included)

117. Do you agree with this recommendation ?

- ◯ No
- ◯ Yes
- ◯ Yes, with the following correction

118. What is the strength of this recommendation ?

- ◯ it must be applied widely and to all patients with this specific need
- ◯ it is important, but can be applied not to all patients with this specific need
- ◯ less important, it can be applied on a voluntary basis
- ◯ very low importance

Assessment

It is recommended that the evaluation of the angle of trunk rotation is used to assess the effectiveness of conservative treatment

119. Do you agree with this recommendation ?

- No
- Yes
- Yes, with the following correction

120. What is the strength of this recommendation ?

- it must be applied widely and to all patients with this specific need
- it is important, but can be applied not to all patients with this specific need
- less important, it can be applied on a voluntary basis
- very low importance

Assessment

It is recommended that evaluation of angle of trunk rotation is carried out by clinicians specialized in spinal deformities (physicians, physiotherapists)

121. Do you agree with this recommendation ?

- No
- Yes
- Yes, with the following correction

122. What is the strength of this recommendation ?

- it must be applied widely and to all patients with this specific need
- it is important, but can be applied not to all patients with this specific need
- less important, it can be applied on a voluntary basis
- very low importance

Assessment

It is recommended that patients are always examined by the same clinicians specialized in spinal deformities. In settings in which this is not possible, it is recommended regular standardization and validation processes of the methods used

123. Do you agree with this recommendation ?

- No
- Yes
- Yes, with the following correction

124. What is the strength of this recommendation ?

- it must be applied widely and to all patients with this specific need
- it is important, but can be applied not to all patients with this specific need
- less important, it can be applied on a voluntary basis
- very low importance

Assessment

It is recommended not to perform x-rays if the Adam's test is negative and the Scoliometer value is below 5°, unless otherwise justified in the opinion of a clinician specialized in conservative treatment of spinal deformities

125. Do you agree with this recommendation ?

- ◯ No
- ◯ Yes
- ◯ Yes, with the following correction

126. What is the strength of this recommendation ?

- ◯ it must be applied widely and to all patients with this specific need
- ◯ it is important, but can be applied not to all patients with this specific need
- ◯ less important, it can be applied on a voluntary basis
- ◯ very low importance

Assessment

It is recommended that, every time they evaluate children aged from 8 to 15 years, pediatricians, general practitioners and sports physicians perform the Adam's test for scoliosis screening purposes, using the Scoliometer

127. Do you agree with this recommendation ?

- ◯ No
- ◯ Yes
- ◯ Yes, with the following correction

128. What is the strength of this recommendation ?

- ◯ it must be applied widely and to all patients with this specific need
- ◯ it is important, but can be applied not to all patients with this specific need
- ◯ less important, it can be applied on a voluntary basis
- ◯ very low importance

Assessment

It is recommended for clinical follow-up the use of validated methods of assessment and standard clinical data collection forms

129. Do you agree with this recommendation ?

- No
- Yes
- Yes, with the following correction

130. What is the strength of this recommendation ?

- it must be applied widely and to all patients with this specific need
- it is important, but can be applied not to all patients with this specific need
- less important, it can be applied on a voluntary basis
- very low importance

Assessment

It is recommended to take into account the measurement error appointed for each method applied for the assessment of scoliosis patients

131. Do you agree with this recommendation ?

- No
- Yes
- Yes, with the following correction

132. What is the strength of this recommendation ?

- it must be applied widely and to all patients with this specific need
- it is important, but can be applied not to all patients with this specific need
- less important, it can be applied on a voluntary basis
- very low importance

Assessment

It is recommended to assess in scoliosis patients aesthetics, sagittal alignment of the spine, joint hypermobility/laxity and cardiorespiratory function

133. Do you agree with this recommendation ?

- No
- Yes
- Yes, with the following correction

134. What is the strength of this recommendation ?

- it must be applied widely and to all patients with this specific need
- it is important, but can be applied not to all patients with this specific need
- less important, it can be applied on a voluntary basis
- very low importance

Assessment

It is recommended that for assessment of aesthetics validated methods should be used

135. Do you agree with this recommendation ?

- ◯ No
- ◯ Yes
- ◯ Yes, with the following correction

136. What is the strength of this recommendation ?

- ◯ it must be applied widely and to all patients with this specific need
- ◯ it is important, but can be applied not to all patients with this specific need
- ◯ less important, it can be applied on a voluntary basis
- ◯ very low importance

Assessment

It is recommended that aesthetics is assessed with using surface topography, photography or appropriate questionnaires

137. Do you agree with this recommendation ?

- No
- Yes
- Yes, with the following correction

138. What is the strength of this recommendation ?

- it must be applied widely and to all patients with this specific need
- it is important, but can be applied not to all patients with this specific need
- less important, it can be applied on a voluntary basis
- very low importance

Assessment

The sagittal spine balance should be assessed with X-ray

139. Do you agree with this recommendation ?

- No
- Yes
- Yes, with the following correction

140. What is the strength of this recommendation ?

- it must be applied widely and to all patients with this specific need
- it is important, but can be applied not to all patients with this specific need
- less important, it can be applied on a voluntary basis
- very low importance

Assessment

For clinical evaluation of sagittal balance the digital inclinometer and Spinal Mouse may be used

141. Do you agree with this recommendation ?

- No
- Yes
- Yes, with the following correction

142. What is the strength of this recommendation ?

- it must be applied widely and to all patients with this specific need
- it is important, but can be applied not to all patients with this specific need
- less important, it can be applied on a voluntary basis
- very low importance

Assessment

It is recommended to assess joint hypermobility/laxity with using Beighton score in children qualified to conservative treatment (especially scoliosis specific exercises)

143. Do you agree with this recommendation ?

- ◯ No
- ◯ Yes
- ◯ Yes, with the following correction

144. What is the strength of this recommendation ?

- ◯ it must be applied widely and to all patients with this specific need
- ◯ it is important, but can be applied not to all patients with this specific need
- ◯ less important, it can be applied on a voluntary basis
- ◯ very low importance

Assessment

It is recommended to assess function of cardiorespiratory system and physical capacity in scoliosis patients
LoE: III

145. Do you think this recommendation should be rephrased?

- ◯ No
- ◯ Yes, with the following correction

146. What is the strength of this recommendation ?

- ◯ it must be applied widely and to all patients with this specific need
- ◯ it is important, but can be applied not to all patients with this specific need
- ◯ less important, it can be applied on a voluntary basis
- ◯ very low importance

Assessment

It is recommended during growth that clinical follow-up examinations are performed at least twice a year, a part periods of rapid growth (pubertal spurt, first three years of life)

147. Do you agree with this recommendation ?

- No
- Yes
- Yes, with the following correction

148. What is the strength of this recommendation ?

- it must be applied widely and to all patients with this specific need
- it is important, but can be applied not to all patients with this specific need
- less important, it can be applied on a voluntary basis
- very low importance

Assessment

It is recommended that the decision whether to perform a radiographic study should be made by a physician specialized in spinal deformities

149. Do you agree with this recommendation ?

- No
- Yes
- Yes, with the following correction

150. What is the strength of this recommendation ?

- it must be applied widely and to all patients with this specific need
- it is important, but can be applied not to all patients with this specific need
- less important, it can be applied on a voluntary basis
- very low importance

Assessment

It is recommended that frontal radiographic studies are made postero-anteriorly, using digital films with a ratio x-rays, including visualization of the femoral heads and protection of the gonads, in any standing position without the use of support aids or indication of correct posture, unless otherwise justified in the opinion of a clinician specialized in spinal deformities

151. Do you agree with this recommendation ?

- ◯ No
- ◯ Yes
- ◯ Yes, with the following correction

152. What is the strength of this recommendation ?

- ◯ it must be applied widely and to all patients with this specific need
- ◯ it is important, but can be applied not to all patients with this specific need
- ◯ less important, it can be applied on a voluntary basis
- ◯ very low importance

Assessment

It is recommended that curve magnitude is measured using the Cobb method

153. Do you agree with this recommendation ?

- ◯ No
- ◯ Yes
- ◯ Yes, with the following correction

154. What is the strength of this recommendation ?

- ◯ it must be applied widely and to all patients with this specific need
- ◯ it is important, but can be applied not to all patients with this specific need
- ◯ less important, it can be applied on a voluntary basis
- ◯ very low importance

Assessment

It is recommended that vertebral rotation is measured on the apical vertebra using either the Perdriolle torsiometer or the Raimondi tables/ruler

155. Do you agree with this recommendation ?

- No
- Yes
- Yes, with the following correction

156. What is the strength of this recommendation ?

- it must be applied widely and to all patients with this specific need
- it is important, but can be applied not to all patients with this specific need
- less important, it can be applied on a voluntary basis
- very low importance

Assessment

It is recommended that the first and last radiographic evaluation include also a standing lateral view

157. Do you agree with this recommendation ?

- ◯ No
- ◯ Yes
- ◯ Yes, with the following correction

158. What is the strength of this recommendation ?

- ◯ it must be applied widely and to all patients with this specific need
- ◯ it is important, but can be applied not to all patients with this specific need
- ◯ less important, it can be applied on a voluntary basis
- ◯ very low importance

Assessment

On radiographic lateral view, the patient’s upper extremities should be placed in a position to uncover the upper thoracic spine. The recommended positions comprise: (1) 45° angle flexion of the arms, elbows extended and hands resting on a support to preserve the sagittal curvature of the spine, (2) the arms crossed over the breasts , (3) the hand resting on the ipsilateral shoulder without pressing it

159. Do you agree with this recommendation ?

- ○ No
- ○ Yes
- ○ Yes, with the following correction

160. What is the strength of this recommendation ?

- ○ it must be applied widely and to all patients with this specific need
- ○ it is important, but can be applied not to all patients with this specific need
- ○ less important, it can be applied on a voluntary basis
- ○ very low importance

Assessment

To reduce the invasiveness of follow-up, it is recommended that no more than 1 radiographic study per year should be performed, unless it is truly necessary and is decided by a clinician specialized in spinal diseases

161. Do you agree with this recommendation ?

- No
- Yes
- Yes, with the following correction

162. What is the strength of this recommendation ?

- it must be applied widely and to all patients with this specific need
- it is important, but can be applied not to all patients with this specific need
- less important, it can be applied on a voluntary basis
- very low importance

Assessment

To reduce the invasiveness of follow-up, it is recommended that the least number of projections is made on radiographic studies

163. Do you agree with this recommendation ?

- ◯ No
- ◯ Yes
- ◯ Yes, with the following correction

164. What is the strength of this recommendation ?

- ◯ it must be applied widely and to all patients with this specific need
- ◯ it is important, but can be applied not to all patients with this specific need
- ◯ less important, it can be applied on a voluntary basis
- ◯ very low importance

Assessment

It is recommended that all idiopathic scoliosis patients, even if not treated, are regularly followed-up

165. Do you agree with this recommendation ?

- ◯ No
- ◯ Yes
- ◯ Yes, with the following correction

166. What is the strength of this recommendation ?

- ◯ it must be applied widely and to all patients with this specific need
- ◯ it is important, but can be applied not to all patients with this specific need
- ◯ less important, it can be applied on a voluntary basis
- ◯ very low importance

Assessment

167. Do you suggest any other relevant recommendation on Assessment?

168. Do you suggest any other relevant recommendation on Assessment ?

169. Do you suggest any other relevant recommendation on Assessment ?

170. Do you suggest any other relevant recommendation on Assessment ?

171. Do you suggest any other relevant recommendation on Assessment ?
