# Supplementary material for: 2016 SOSORT guidelines: orthopaedic and rehabilitation treatment of idiopathic scoliosis during growth
Source: Scoliosis Spinal Disord. 2018 Jan 10;13:3. doi: 10.1186/s13013-017-0145-8 (PMC5795289; doi:10.1186/s13013-017-0145-8)
Supplement: Supplementary file 4 — Questionnaires used for Dephi procedure, from Round 1 to round 3. (PDF 179 kb) [file 13013_2017_145_MOESM4_ESM.pdf]

Bracing

It is recommended that scoliosis specific exercises are prescribed to patients wearing braces

1. Do you agree with this recommendation ?

- ◯ No
- ◯ Yes
- ◯ Yes, with suggestions

2. What is the strength of this recommendation ?

- ◯ it must be applied widely and to all patients with this specific need
- ◯ it is important, but can be applied not to all patients with this specific need
- ◯ less important, it can be applied on a voluntary basis
- ◯ very low importance

Bracing

It is recommended that braces are regularly changed according to growth (maximum acceptable changes ... cm., ... kg, ... months) and/or specific pathological needs as judged by a scoliosis expert physician. A brace change should be free of charge if occurring before ... months.

3. What is the number of months that makes mandatory for you to change a brace ?

- It is inappropriate to define such a value - individual expert choice
- I don't know
- Number of months

4. What is the number of cm of height growth that makes mandatory for you to change a brace ?

- It is inappropriate to define such a value - individual expert choice
- I don't know

Number of centimeters

5. What is the number of kg of weight growth that makes mandatory for you to change a brace ?

- It is inappropriate to define such a value - individual expert choice
- I don't know
- Number of chilograms

6. What is the minimum number of months that should be defined before paying a change of brace ? Eplanation: if a brace is totally inappropriate should be changed immediately free of charge - so how long can it take to make a change needed for pathological and/or growth reasons ?

- It is inappropriate to define such a value - individual expert choice
- I don't know
- Number of months

7. Do you agree with this recommendation ? (numerical value will be inserted ONLY if felt appropriate by at least 80% of respondents)

- ◯ No
- ◯ Yes
- ◯ Yes, with suggestions

8. What is the strength of this recommendation ?

- ◯ it must be applied widely and to all patients with this specific need
- ◯ it is important, but can be applied not to all patients with this specific need
- ◯ less important, it can be applied on a voluntary basis
- ◯ very low importance

Bracing

It is recommended that out of brace x-rays are regularly performed to check the effectiveness of bracing treatment: the number of hours out of brace before x-ray taking should correspond to the daily weaning time

9. Do you agree with this recommendation ?

- ◯ No
- ◯ Yes
- ◯ Yes, with suggestions

10. Do you agree to leave the second part of the recommendation "the number of hours out of brace before x-ray taking should correspond to the daily weaning time" ?

- ◯ No
- ◯ Yes
- ◯ Yes, with suggestions

11. What is the strength of this recommendation ?

- ◯ it must be applied widely and to all patients with this specific need
- ◯ it is important, but can be applied not to all patients with this specific need
- ◯ less important, it can be applied on a voluntary basis
- ◯ very low importance

Bracing

It is recommended that brace is definitively weaned when bone maturity is reached and results are checked with an x--ray after 48 hours without wearing the brace

12. Do you agree to define:

- [ ] bone growth until Risser 3
- [ ] bone maturity until Risser 5 or ringaposhysis closure
- [ ] none of the above

13. Do you agree with this recommendation ?

- ◯ No
- ◯ Yes
- ◯ Yes, with suggestions

14. What is the strength of this recommendation ?

- ◯ it must be applied widely and to all patients with this specific need
- ◯ it is important, but can be applied not to all patients with this specific need
- ◯ less important, it can be applied on a voluntary basis
- ◯ very low importance

Physiotherapeutic Scoliosis Specific Exercises

It is recommended that therapists implement a compliance system for exercise tracking

15. Do you agree with this recommendation ?

- ◯ No
- ◯ Yes
- ◯ Yes, with suggestons

16. What is the strength of this recommendation ?

- ◯ it must be applied widely and to all patients with this specific need
- ◯ it is important, but can be applied not to all patients with this specific need
- ◯ less important, it can be applied on a voluntary basis
- ◯ very low importance

Physiotherapeutic Scoliosis Specific Exercises

It is recommended that therapists regularly assess patients quality of Physiotherapeutic Scoliosis Specific Exercises performed by the patients

17. Do you agree with this recommendation ?

- No
- Yes
- Yes, with suggestons

18. What is the strength of this recommendation ?

- it must be applied widely and to all patients with this specific need
- it is important, but can be applied not to all patients with this specific need
- less important, it can be applied on a voluntary basis
- very low importance

Physiotherapeutic Scoliosis Specific Exercises

It is recommented that Physiotherapeutic Scoliosis Specific Exercises difficulty is progressively increased according to patient ability

19. Do you agree with this recommendation ?

- No
- Yes
- Yes, with the suggestions

20. What is the strength of this recommendation ?

- it must be applied widely and to all patients with this specific need
- it is important, but can be applied not to all patients with this specific need
- less important, it can be applied on a voluntary basis
- very low importance

Physiotherapeutic Scoliosis Specific Exercises

It is recommended that Physiotherapeutic Scoliosis Specific Exercises are taught individually in a 1 to 1 relationship to assure individualized care, while regular performance could also be at home or in little groups

21. Do you agree with this recommendation ?

- ◯ No
- ◯ Yes
- ◯ Yes, with suggestions

22. What is the strength of this recommendation ?

- ◯ it must be applied widely and to all patients with this specific need
- ◯ it is important, but can be applied not to all patients with this specific need
- ◯ less important, it can be applied on a voluntary basis
- ◯ very low importance

Assessment

It is recommended to regularly perform quality of life assessment through validated questionnaires

23. Do you agree with this recommendation ?

- No
- Yes
- Yes, with suggestions

24. What is the strength of this recommendation ?

- it must be applied widely and to all patients with this specific need
- it is important, but can be applied not to all patients with this specific need
- less important, it can be applied on a voluntary basis
- very low importance
