# Supplementary material for: 2016 SOSORT guidelines: orthopaedic and rehabilitation treatment of idiopathic scoliosis during growth
Source: Scoliosis Spinal Disord. 2018 Jan 10;13:3. doi: 10.1186/s13013-017-0145-8 (PMC5795289; doi:10.1186/s13013-017-0145-8)
Supplement: Supplementary file 5 — Questionnaires used for Dephi procedure, from Round 1 to round 3. (PDF 2150 kb) [file 13013_2017_145_MOESM5_ESM.pdf]

Welcome to the Consensus Process for the 2016 SOSORT Guidelines

Dear colleagues

this Consensus process is part of the development of the 2016 SOSORT Guidelines that will adjourne and substitute the 2011 Guidelines (http://scoliosisjournal.biomedcentral.com/articles/10.1186/1748-7161-7-3) and will be published in our official journal Scoliosis and Spinal Deformity.

You will also receive in a second moment the recommendations to be approved.

Please fill in accurately this questionnaire so to make our Guidelines stronger and really representative of our community of expert in conservative orthopedic and rehabilitation scoliosis treatment.

Thank you for participating in our Consensus. Your feedback is important.

Introduction

1. What is your family name ?

2. What is your first name?

3. What is your gender?

- ◯ Female
- ◯ Male

4. What is your age?

- ◯ 18 to 24
- ◯ 25 to 34
- ◯ 35 to 44
- ◯ 45 to 54
- ◯ 55 to 64
- ◯ 65 to 74
- ◯ 75 or older

5. What is your profession ?

- ☐ MD, orthopedic surgeon
- ☐ MD, physical and rehabilitation medicine
- ☐ MD, others
- ☐ Physiotherapist
- ☐ Orthotist
- ☐ PhD
- ☐ Other (please specify)

6. In what country do you work?

Age classification

7. Can you accept the following classification as clinically relevant for conservative orthopaedic and rehabilitation treatment of idiopathic scoliosis during growth (ie: you change your clinical decisions according to this classification) ?

Chronological (age at diagnosis)
- Infantile 0-2 years 11 months
- Juvenile 3-9 years 11 months
- Adolescent 10-17 years 11 months
- Adult 18 or more

- ◯ Yes
- ◯ No
- ◯ Other (please specify)

Angular classification

8. With the aim to produce an angular classification of idiopathic scoliosis during growth, how would you define a curve below 15° ? (you can use more than one choice)

- [ ] Low degree
- [ ] Moderate degree
- [ ] Severe degree
- [ ] Very severe degree

9. With the aim to produce an angular classification of idiopathic scoliosis during growth, how would you define a curve between 16° and 20° ? (you can use more than one choice)

- [ ] Low degree
- [ ] Moderate degree
- [ ] Severe degree
- [ ] Very severe degree

10. With the aim to produce an angular classification of idiopathic scoliosis during growth, how would you define a curve between 21° and 25° ? (you can use more than one choice)

- [ ] Low degree
- [ ] Moderate degree
- [ ] Severe degree
- [ ] Very severe degree

11. With the aim to produce an angular classification of idiopathic scoliosis during growth, how would you define a curve between 26° and 30° ? (you can use more than one choice)

- [ ] Low degree
- [ ] Moderate degree
- [ ] Severe degree
- [ ] Very severe degree

12. With the aim to produce an angular classification of idiopathic scoliosis during growth, how would you define a curve between 31° and 35° ? (you can use more than one choice)

- [ ] Low degree
- [ ] Moderate degree
- [ ] Severe degree
- [ ] Very severe degree

13. With the aim to produce an angular classification of idiopathic scoliosis during growth, how would you define a curve between 36° and 40° ? (you can use more than one choice)

- [ ] Low degree
- [ ] Moderate degree
- [ ] Severe degree
- [ ] Very severe degree

14. With the aim to produce an angular classification of idiopathic scoliosis during growth, how would you define a curve between 41° and 45° ? (you can use more than one choice)

- [ ] Low degree
- [ ] Moderate degree
- [ ] Severe degree
- [ ] Very severe degree

15. With the aim to produce an angular classification of idiopathic scoliosis during growth, how would you define a curve between 46° and 50° ? (you can use more than one choice)

- [ ] Low degree
- [ ] Moderate degree
- [ ] Severe degree
- [ ] Very severe degree

16. With the aim to produce an angular classification of idiopathic scoliosis during growth, how would you define a curve between 51° and 55° ? (you can use more than one choice)

- [ ] Low degree
- [ ] Moderate degree
- [ ] Severe degree
- [ ] Very severe degree

17. With the aim to produce an angular classification of idiopathic scoliosis during growth, how would you define a curve between 56° and 60° ? (you can use more than one choice)

- [ ] Low degree
- [ ] Moderate degree
- [ ] Severe degree
- [ ] Very severe degree

18. With the aim to produce an angular classification of idiopathic scoliosis during growth, how would you define a curve above 60° ? (you can use more than one choice)

- [ ] Low degree
- [ ] Moderate degree
- [ ] Severe degree
- [ ] Very severe degree

Other classifications

19. Can you accept the following classification of Ponseti as clinically relevant for conservative otrhopaedic and rehabilitation treatment of idiopathic scoliosis during growth (ie: you change your clinical decisions according to this classification) ?

Topographic (Apex)
- Cervical (to disc C6-7)
- Cervico-thoracic (C7 to T1)
- Thoracic (disc T1-2 to disc T11-12)
- Thoraco-lumbar (T12 to L1)
- Lumbar (from disc L1-2)

○ Yes

○ No

○ Other (please specify)

20. Which one of the following classifications do you think is clinically relevant for conservative orthopaedic and rehabilitation treatment of idiopathic scoliosis during growth ?

(tick the classifications that you think are clinically relevant)

☐ Rigo Classification (clinical + radiological)

☐ Schroth Classification

☐ Lenke classification

☐ King classification

☐ Schwab classification

☐ Aebi classification

☐ SpineCor classification

☐ Other (please specify)

Aims of treatment during growth

21. Tick the main aims of conservative (orthopedic and rehabilitation) scoliosis treatment during growth - ie the aims that you would like to reach independently of the radiograpihc degree of the curve

- [ ] Avoid surgery
- [ ] Improve aesthetics
- [ ] Improve quality of life
- [ ] Reduce disability
- [ ] Reduce pain
- [ ] Avoid pulmonary complications
- [ ] Others (please specify)

22. Tick the primary radiographic aims of conservative (orthopedic and rehabilitation) scoliosis treatment in curves of LOW degree during growth - ie what realistically you would like to reach with your patients

- ○ Straighten the spine (below 10°)
- ○ Remain below 20°
- ○ Remain below 30°
- ○ Remain below 45°
- ○ Others (please specify)

23. Tick the secondary radiographic aims of conservative (orthopedic and rehabilitation) scoliosis treatment in curves of LOW degree during growth - ie what you would like to reach as a second option if the primary aim is not possible due to progression

- ○ Straighten the spine (below 10°)
- ○ Remain below 20°
- ○ Remain below 30°
- ○ Remain below 45°
- ○ Others (please specify)

24. Tick the primary radiographic aims of conservative (orthopedic and rehabilitation) scoliosis treatment in curves of MODERATE degree during growth - ie what realistically you would like to reach with your patients

- ◯ Straighten the spine (below 10°)
- ◯ Remain below 20°
- ◯ Remain below 30°
- ◯ Remain below 45°
- ◯ Yes with the following changes

25. Tick the secondary radiographic aims of conservative (orthopedic and rehabilitation) scoliosis treatment in curves of MODERATE degree during growth - ie what you would like to reach as a second option if the primary aim is not possible due to progression

- ◯ Straighten the spine (below 10°)
- ◯ Remain below 20°
- ◯ Remain below 30°
- ◯ Remain below 45°
- ◯ Yes with the following changes

26. Tick the primary radiographic aims of conservative (orthopedic and rehabilitation) scoliosis treatment in curves of SEVERE degree during growth - ie what realistically you would like to reach with your patients

- ◯ Straighten the spine (below 10°)
- ◯ Remain below 20°
- ◯ Remain below 30°
- ◯ Remain below 45°
- ◯ Yes with the following changes

27. Tick the secondary radiographic aims of conservative (orthopedic and rehabilitation) scoliosis treatment in curves of SEVERE degree during growth - ie what you would like to reach as a second option if the primary aim is not possible due to progression

- ◯ Straighten the spine (below 10°)
- ◯ Remain below 20°
- ◯ Remain below 30°
- ◯ Remain below 45°
- ◯ Others (please specify)

Strength of treatments

We decided to make more simple the table of possible treatments for Idiopathic scoliosis of the 2011 Guidelines. For this puropose we propose to use the same Angular classification of Question 2 to reduce the cathegories. In the meantime we will maintain the minimum and maximum range of possible treatments as previously agreed.

We use here the same hypothetical classification of progression of strength of treatment as proposed in the previous Guidelines according to the Step-by-step Sibilla theory. We introduced the classification of Rigid Bracing timing approved in the SRS-SOSORT Consensus (Night Time, Home Time, Part Time, Full Time, Total Time). This progression of forces can be wrong, and finding an agreement on it proved to be impossible in the past. Consequently, we try again here, and we will then eventually adapt the list of treatments. If i twill not be possible to find an agreement, we will maintain this classification as a simple communication tool.

28. Classify in the table below the progression of forces you would consider for the treatments listed above. The actual proposal is reported from 1 (less intensive treatment) to 14 (more intensive treatment). Simply write the numbers from 1 (less intensive treatment) to 14 (more intensive treatment) according to your preference

- [ ] Obs36 - Observation every 36 months
- [ ] Obs12 - Observation every 12 months
- [ ] Obs8 - Observation every 8 months
- [ ] Obs6 - Observation every 6 months
- [ ] Obs3 - Observation every 3 months
- [ ] PSSE - Physiotherapeutic Scoliosis-Specific Exercises
- [ ] SIR - Scoliosis Inpatient Rehabilitation
- [ ] NTRB - Night-Time Rigid Bracing (in bed only)
- [ ] HTRB - Home-Time Rigid Bracing (up to 14 hours/day)
- [ ] SSB - Specific Soft Bracing
- [ ] PTRB - Part-Time Rigid Bracing (15-18 hours/day)
- [ ] FTRB - Full-Time Rigid Bracing (19-22 hours/day)
- [ ] TTRB - Total-Time Rigid Bracing (23-25 hours/day)- Casting
- [ ] Su - Surgery

INFANTILE IDIOPATHIC SCOLIOSIS

29. Tick the MINIMUM treatment that you would use in INFANTILE SCOLIOSIS - Low degree

- ◯ Nothing
- ◯ Observation every 36 months
- ◯ Observation every 12 months
- ◯ Observation every 8 months
- ◯ Observation every 6 months
- ◯ Observation every 3 months
- ◯ Physiotherapeutic Scoliosis-Specific Exercises
- ◯ Scoliosis Inpatient Rehabilitation
- ◯ Night-Time Rigid Bracing (in bed only)
- ◯ Home-Time Rigid Bracing (up to 14 hours/day)
- ◯ Specific Soft Bracing
- ◯ Part-Time Rigid Bracing (15-18 hours/day)
- ◯ Full-Time Rigid Bracing (19-22 hours/day)
- ◯ Total-Time Rigid Bracing (23-25 hours/day)- Casting
- ◯ Surgery

30. Tick the MOST STRONG treatment that you would use in INFANTILE SCOLIOSIS - Low degree

- ◯ Nothing
- ◯ Observation every 36 months
- ◯ Observation every 12 months
- ◯ Observation every 8 months
- ◯ Observation every 6 months
- ◯ Observation every 3 months
- ◯ Physiotherapeutic Scoliosis-Specific Exercises
- ◯ Scoliosis Inpatient Rehabilitation
- ◯ Night-Time Rigid Bracing (in bed only)
- ◯ Home-Time Rigid Bracing (up to 14 hours/day)
- ◯ Specific Soft Bracing
- ◯ Part-Time Rigid Bracing (15-18 hours/day)
- ◯ Full-Time Rigid Bracing (19-22 hours/day)
- ◯ Total-Time Rigid Bracing (23-25 hours/day)- Casting
- ◯ Surgery

31. Tick the MINIMUM treatment that you would use in INFANTILE SCOLIOSIS - Moderate degree

- ◯ Nothing
- ◯ Observation every 36 months
- ◯ Observation every 12 months
- ◯ Observation every 8 months
- ◯ Observation every 6 months
- ◯ Observation every 3 months
- ◯ Physiotherapeutic Scoliosis-Specific Exercises
- ◯ Scoliosis Inpatient Rehabilitation
- ◯ Night-Time Rigid Bracing (in bed only)
- ◯ Home-Time Rigid Bracing (up to 14 hours/day)
- ◯ Specific Soft Bracing
- ◯ Part-Time Rigid Bracing (15-18 hours/day)
- ◯ Full-Time Rigid Bracing (19-22 hours/day)
- ◯ Total-Time Rigid Bracing (23-25 hours/day)- Casting
- ◯ Surgery

32. Tick the MOST STRONG treatment that you would use in INFANTILE SCOLIOSIS - Moderate degree

- ◯ Nothing
- ◯ Observation every 36 months
- ◯ Observation every 12 months
- ◯ Observation every 8 months
- ◯ Observation every 6 months
- ◯ Observation every 3 months
- ◯ Physiotherapeutic Scoliosis-Specific Exercises
- ◯ Scoliosis Inpatient Rehabilitation
- ◯ Night-Time Rigid Bracing (in bed only)
- ◯ Home-Time Rigid Bracing (up to 14 hours/day)
- ◯ Specific Soft Bracing
- ◯ Part-Time Rigid Bracing (15-18 hours/day)
- ◯ Full-Time Rigid Bracing (19-22 hours/day)
- ◯ Total-Time Rigid Bracing (23-25 hours/day)- Casting
- ◯ Surgery

33. Tick the MINIMUM treatment that you would use in INFANTILE SCOLIOSIS - Severe degree

- ◯ Nothing
- ◯ Observation every 36 months
- ◯ Observation every 12 months
- ◯ Observation every 8 months
- ◯ Observation every 6 months
- ◯ Observation every 3 months
- ◯ Physiotherapeutic Scoliosis-Specific Exercises
- ◯ Scoliosis Inpatient Rehabilitation
- ◯ Night-Time Rigid Bracing (in bed only)
- ◯ Home-Time Rigid Bracing (up to 14 hours/day)
- ◯ Specific Soft Bracing
- ◯ Part-Time Rigid Bracing (15-18 hours/day)
- ◯ Full-Time Rigid Bracing (19-22 hours/day)
- ◯ Total-Time Rigid Bracing (23-25 hours/day)- Casting
- ◯ Surgery

34. Tick the MOST STRONG treatment that you would use in INFANTILE SCOLIOSIS - Severe degree

- Nothing
- Observation every 36 months
- Observation every 12 months
- Observation every 8 months
- Observation every 6 months
- Observation every 3 months
- Physiotherapeutic Scoliosis-Specific Exercises
- Scoliosis Inpatient Rehabilitation
- Night-Time Rigid Bracing (in bed only)
- Home-Time Rigid Bracing (up to 14 hours/day)
- Specific Soft Bracing
- Part-Time Rigid Bracing (15-18 hours/day)
- Full-Time Rigid Bracing (19-22 hours/day)
- Total-Time Rigid Bracing (23-25 hours/day)- Casting
- Surgery

35. Tick the MINIMUM treatment that you would use in JUVENILE SCOLIOSIS - Low degree

- Nothing
- Observation every 36 months
- Observation every 12 months
- Observation every 8 months
- Observation every 6 months
- Observation every 3 months
- Physiotherapeutic Scoliosis-Specific Exercises
- Scoliosis Inpatient Rehabilitation
- Night-Time Rigid Bracing (in bed only)
- Home-Time Rigid Bracing (up to 14 hours/day)
- Specific Soft Bracing
- Part-Time Rigid Bracing (15-18 hours/day)
- Full-Time Rigid Bracing (19-22 hours/day)
- Total-Time Rigid Bracing (23-25 hours/day)- Casting
- Surgery

36. Tick the MOST STRONG treatment that you would use in JUVENILE SCOLIOSIS - Low degree

- Nothing
- Observation every 36 months
- Observation every 12 months
- Observation every 8 months
- Observation every 6 months
- Observation every 3 months
- Physiotherapeutic Scoliosis-Specific Exercises
- Scoliosis Inpatient Rehabilitation
- Night-Time Rigid Bracing (in bed only)
- Home-Time Rigid Bracing (up to 14 hours/day)
- Specific Soft Bracing
- Part-Time Rigid Bracing (15-18 hours/day)
- Full-Time Rigid Bracing (19-22 hours/day)
- Total-Time Rigid Bracing (23-25 hours/day)- Casting
- Surgery

37. Tick the MINIMUM treatment that you would use in JUVENILE SCOLIOSIS - Moderate degree

- Nothing
- Observation every 36 months
- Observation every 12 months
- Observation every 8 months
- Observation every 6 months
- Observation every 3 months
- Physiotherapeutic Scoliosis-Specific Exercises
- Scoliosis Inpatient Rehabilitation
- Night-Time Rigid Bracing (in bed only)
- Home-Time Rigid Bracing (up to 14 hours/day)
- Specific Soft Bracing
- Part-Time Rigid Bracing (15-18 hours/day)
- Full-Time Rigid Bracing (19-22 hours/day)
- Total-Time Rigid Bracing (23-25 hours/day)- Casting
- Surgery

38. Tick the MOST STRONG treatment that you would use in JUVENILE SCOLIOSIS - Moderate degree

- ◯ Nothing
- ◯ Observation every 36 months
- ◯ Observation every 12 months
- ◯ Observation every 8 months
- ◯ Observation every 6 months
- ◯ Observation every 3 months
- ◯ Physiotherapeutic Scoliosis-Specific Exercises
- ◯ Scoliosis Inpatient Rehabilitation
- ◯ Night-Time Rigid Bracing (in bed only)
- ◯ Home-Time Rigid Bracing (up to 14 hours/day)
- ◯ Specific Soft Bracing
- ◯ Part-Time Rigid Bracing (15-18 hours/day)
- ◯ Full-Time Rigid Bracing (19-22 hours/day)
- ◯ Total-Time Rigid Bracing (23-25 hours/day)- Casting
- ◯ Surgery

39. Tick the MINIMUM treatment that you would use in JUVENILE SCOLIOSIS - Severe degree

- ◯ Nothing
- ◯ Observation every 36 months
- ◯ Observation every 12 months
- ◯ Observation every 8 months
- ◯ Observation every 6 months
- ◯ Observation every 3 months
- ◯ Physiotherapeutic Scoliosis-Specific Exercises
- ◯ Scoliosis Inpatient Rehabilitation
- ◯ Night-Time Rigid Bracing (in bed only)
- ◯ Home-Time Rigid Bracing (up to 14 hours/day)
- ◯ Specific Soft Bracing
- ◯ Part-Time Rigid Bracing (15-18 hours/day)
- ◯ Full-Time Rigid Bracing (19-22 hours/day)
- ◯ Total-Time Rigid Bracing (23-25 hours/day)- Casting
- ◯ Surgery

40. Tick the MOST STRONG treatment that you would use in JUVENILE SCOLIOSIS - Severe degree

- Nothing
- Observation every 36 months
- Observation every 12 months
- Observation every 8 months
- Observation every 6 months
- Observation every 3 months
- Physiotherapeutic Scoliosis-Specific Exercises
- Scoliosis Inpatient Rehabilitation
- Night-Time Rigid Bracing (in bed only)
- Home-Time Rigid Bracing (up to 14 hours/day)
- Specific Soft Bracing
- Part-Time Rigid Bracing (15-18 hours/day)
- Full-Time Rigid Bracing (19-22 hours/day)
- Total-Time Rigid Bracing (23-25 hours/day)- Casting
- Surgery

ADOLESCENT IDIOPATHIC SCOLIOSIS - Risser 0

41. Tick the MINIMUM treatment that you would use in ADOLESCENT SCOLIOSIS Risser 0 - Low degree

- ◯ Nothing
- ◯ Observation every 36 months
- ◯ Observation every 12 months
- ◯ Observation every 8 months
- ◯ Observation every 6 months
- ◯ Observation every 3 months
- ◯ Physiotherapeutic Scoliosis-Specific Exercises
- ◯ Scoliosis Inpatient Rehabilitation
- ◯ Night-Time Rigid Bracing (in bed only)
- ◯ Home-Time Rigid Bracing (up to 14 hours/day)
- ◯ Specific Soft Bracing
- ◯ Part-Time Rigid Bracing (15-18 hours/day)
- ◯ Full-Time Rigid Bracing (19-22 hours/day)
- ◯ Total-Time Rigid Bracing (23-25 hours/day)- Casting
- ◯ Surgery

42. Tick the MOST STRONG treatment that you would use in ADOLESCENT SCOLIOSIS Risser 0 - Low degree

- ◯ Nothing
- ◯ Observation every 36 months
- ◯ Observation every 12 months
- ◯ Observation every 8 months
- ◯ Observation every 6 months
- ◯ Observation every 3 months
- ◯ Physiotherapeutic Scoliosis-Specific Exercises
- ◯ Scoliosis Inpatient Rehabilitation
- ◯ Night-Time Rigid Bracing (in bed only)
- ◯ Home-Time Rigid Bracing (up to 14 hours/day)
- ◯ Specific Soft Bracing
- ◯ Part-Time Rigid Bracing (15-18 hours/day)
- ◯ Full-Time Rigid Bracing (19-22 hours/day)
- ◯ Total-Time Rigid Bracing (23-25 hours/day)- Casting
- ◯ Surgery

43. Tick the MINIMUM treatment that you would use in ADOLESCENT SCOLIOSIS Risser 0 - Moderate degree

- Nothing
- Observation every 36 months
- Observation every 12 months
- Observation every 8 months
- Observation every 6 months
- Observation every 3 months
- Physiotherapeutic Scoliosis-Specific Exercises
- Scoliosis Inpatient Rehabilitation
- Night-Time Rigid Bracing (in bed only)
- Home-Time Rigid Bracing (up to 14 hours/day)
- Specific Soft Bracing
- Part-Time Rigid Bracing (15-18 hours/day)
- Full-Time Rigid Bracing (19-22 hours/day)
- Total-Time Rigid Bracing (23-25 hours/day)- Casting
- Surgery

44. Tick the MOST STRONG treatment that you would use in ADOLESCENT SCOLIOSIS Risser 0 - Moderate degree

- ◯ Nothing
- ◯ Observation every 36 months
- ◯ Observation every 12 months
- ◯ Observation every 8 months
- ◯ Observation every 6 months
- ◯ Observation every 3 months
- ◯ Physiotherapeutic Scoliosis-Specific Exercises
- ◯ Scoliosis Inpatient Rehabilitation
- ◯ Night-Time Rigid Bracing (in bed only)
- ◯ Home-Time Rigid Bracing (up to 14 hours/day)
- ◯ Specific Soft Bracing
- ◯ Part-Time Rigid Bracing (15-18 hours/day)
- ◯ Full-Time Rigid Bracing (19-22 hours/day)
- ◯ Total-Time Rigid Bracing (23-25 hours/day)- Casting
- ◯ Surgery

45. Tick the MINIMUM treatment that you would use in ADOLESCENT SCOLIOSIS Risser 0 - Severe degree

- ◯ Nothing
- ◯ Observation every 36 months
- ◯ Observation every 12 months
- ◯ Observation every 8 months
- ◯ Observation every 6 months
- ◯ Observation every 3 months
- ◯ Physiotherapeutic Scoliosis-Specific Exercises
- ◯ Scoliosis Inpatient Rehabilitation
- ◯ Night-Time Rigid Bracing (in bed only)
- ◯ Home-Time Rigid Bracing (up to 14 hours/day)
- ◯ Specific Soft Bracing
- ◯ Part-Time Rigid Bracing (15-18 hours/day)
- ◯ Full-Time Rigid Bracing (19-22 hours/day)
- ◯ Total-Time Rigid Bracing (23-25 hours/day)- Casting
- ◯ Surgery

46. Tick the MOST STRONG treatment that you would use in ADOLESCENT SCOLIOSIS Risser 0 - Severe degree

- Nothing
- Observation every 36 months
- Observation every 12 months
- Observation every 8 months
- Observation every 6 months
- Observation every 3 months
- Physiotherapeutic Scoliosis-Specific Exercises
- Scoliosis Inpatient Rehabilitation
- Night-Time Rigid Bracing (in bed only)
- Home-Time Rigid Bracing (up to 14 hours/day)
- Specific Soft Bracing
- Part-Time Rigid Bracing (15-18 hours/day)
- Full-Time Rigid Bracing (19-22 hours/day)
- Total-Time Rigid Bracing (23-25 hours/day)- Casting
- Surgery

ADOLESCENT IDIOPATHIC SCOLIOSIS - Risser 1

47. Tick the MINIMUM treatment that you would use in ADOLESCENT SCOLIOSIS Risser 1 - Low degree

- Nothing
- Observation every 36 months
- Observation every 12 months
- Observation every 8 months
- Observation every 6 months
- Observation every 3 months
- Physiotherapeutic Scoliosis-Specific Exercises
- Scoliosis Inpatient Rehabilitation
- Night-Time Rigid Bracing (in bed only)
- Home-Time Rigid Bracing (up to 14 hours/day)
- Specific Soft Bracing
- Part-Time Rigid Bracing (15-18 hours/day)
- Full-Time Rigid Bracing (19-22 hours/day)
- Total-Time Rigid Bracing (23-25 hours/day)- Casting
- Surgery

48. Tick the MOST STRONG treatment that you would use in ADOLESCENT SCOLIOSIS Risser 1 - Low degree

- Nothing
- Observation every 36 months
- Observation every 12 months
- Observation every 8 months
- Observation every 6 months
- Observation every 3 months
- Physiotherapeutic Scoliosis-Specific Exercises
- Scoliosis Inpatient Rehabilitation
- Night-Time Rigid Bracing (in bed only)
- Home-Time Rigid Bracing (up to 14 hours/day)
- Specific Soft Bracing
- Part-Time Rigid Bracing (15-18 hours/day)
- Full-Time Rigid Bracing (19-22 hours/day)
- Total-Time Rigid Bracing (23-25 hours/day)- Casting
- Surgery

49. Tick the MINIMUM treatment that you would use in ADOLESCENT SCOLIOSIS Risser 1 - Moderate degree

- Nothing
- Observation every 36 months
- Observation every 12 months
- Observation every 8 months
- Observation every 6 months
- Observation every 3 months
- Physiotherapeutic Scoliosis-Specific Exercises
- Scoliosis Inpatient Rehabilitation
- Night-Time Rigid Bracing (in bed only)
- Home-Time Rigid Bracing (up to 14 hours/day)
- Specific Soft Bracing
- Part-Time Rigid Bracing (15-18 hours/day)
- Full-Time Rigid Bracing (19-22 hours/day)
- Total-Time Rigid Bracing (23-25 hours/day)- Casting
- Surgery

50. Tick the MOST STRONG treatment that you would use in ADOLESCENT SCOLIOSIS Risser 1 - Moderate degree

- Nothing
- Observation every 36 months
- Observation every 12 months
- Observation every 8 months
- Observation every 6 months
- Observation every 3 months
- Physiotherapeutic Scoliosis-Specific Exercises
- Scoliosis Inpatient Rehabilitation
- Night-Time Rigid Bracing (in bed only)
- Home-Time Rigid Bracing (up to 14 hours/day)
- Specific Soft Bracing
- Part-Time Rigid Bracing (15-18 hours/day)
- Full-Time Rigid Bracing (19-22 hours/day)
- Total-Time Rigid Bracing (23-25 hours/day)- Casting
- Surgery

51. Tick the MINIMUM treatment that you would use in ADOLESCENT SCOLIOSIS Risser 1 - Severe degree

- Nothing
- Observation every 36 months
- Observation every 12 months
- Observation every 8 months
- Observation every 6 months
- Observation every 3 months
- Physiotherapeutic Scoliosis-Specific Exercises
- Scoliosis Inpatient Rehabilitation
- Night-Time Rigid Bracing (in bed only)
- Home-Time Rigid Bracing (up to 14 hours/day)
- Specific Soft Bracing
- Part-Time Rigid Bracing (15-18 hours/day)
- Full-Time Rigid Bracing (19-22 hours/day)
- Total-Time Rigid Bracing (23-25 hours/day)- Casting
- Surgery

52. Tick the MOST STRONG treatment that you would use in ADOLESCENT SCOLIOSIS Risser 1 - Severe degree

- Nothing
- Observation every 36 months
- Observation every 12 months
- Observation every 8 months
- Observation every 6 months
- Observation every 3 months
- Physiotherapeutic Scoliosis-Specific Exercises
- Scoliosis Inpatient Rehabilitation
- Night-Time Rigid Bracing (in bed only)
- Home-Time Rigid Bracing (up to 14 hours/day)
- Specific Soft Bracing
- Part-Time Rigid Bracing (15-18 hours/day)
- Full-Time Rigid Bracing (19-22 hours/day)
- Total-Time Rigid Bracing (23-25 hours/day)- Casting
- Surgery

ADOLESCENT IDIOPATHIC SCOLIOSIS - Risser 2

53. Tick the MINIMUM treatment that you would use in ADOLESCENT SCOLIOSIS Risser 2 - Low degree

- Nothing
- Observation every 36 months
- Observation every 12 months
- Observation every 8 months
- Observation every 6 months
- Observation every 3 months
- Physiotherapeutic Scoliosis-Specific Exercises
- Scoliosis Inpatient Rehabilitation
- Night-Time Rigid Bracing (in bed only)
- Home-Time Rigid Bracing (up to 14 hours/day)
- Specific Soft Bracing
- Part-Time Rigid Bracing (15-18 hours/day)
- Full-Time Rigid Bracing (19-22 hours/day)
- Total-Time Rigid Bracing (23-25 hours/day)- Casting
- Surgery

54. Tick the MOST STRONG treatment that you would use in ADOLESCENT SCOLIOSIS Risser 2 - Low degree

- ◯ Nothing
- ◯ Observation every 36 months
- ◯ Observation every 12 months
- ◯ Observation every 8 months
- ◯ Observation every 6 months
- ◯ Observation every 3 months
- ◯ Physiotherapeutic Scoliosis-Specific Exercises
- ◯ Scoliosis Inpatient Rehabilitation
- ◯ Night-Time Rigid Bracing (in bed only)
- ◯ Home-Time Rigid Bracing (up to 14 hours/day)
- ◯ Specific Soft Bracing
- ◯ Part-Time Rigid Bracing (15-18 hours/day)
- ◯ Full-Time Rigid Bracing (19-22 hours/day)
- ◯ Total-Time Rigid Bracing (23-25 hours/day)- Casting
- ◯ Surgery

55. Tick the MINIMUM treatment that you would use in ADOLESCENT SCOLIOSIS Risser 2 - Moderate degree

- Nothing
- Observation every 36 months
- Observation every 12 months
- Observation every 8 months
- Observation every 6 months
- Observation every 3 months
- Physiotherapeutic Scoliosis-Specific Exercises
- Scoliosis Inpatient Rehabilitation
- Night-Time Rigid Bracing (in bed only)
- Home-Time Rigid Bracing (up to 14 hours/day)
- Specific Soft Bracing
- Part-Time Rigid Bracing (15-18 hours/day)
- Full-Time Rigid Bracing (19-22 hours/day)
- Total-Time Rigid Bracing (23-25 hours/day)- Casting
- Surgery

56. Tick the MOST STRONG treatment that you would use in ADOLESCENT SCOLIOSIS Risser 2 - Moderate degree

- Nothing
- Observation every 36 months
- Observation every 12 months
- Observation every 8 months
- Observation every 6 months
- Observation every 3 months
- Physiotherapeutic Scoliosis-Specific Exercises
- Scoliosis Inpatient Rehabilitation
- Night-Time Rigid Bracing (in bed only)
- Home-Time Rigid Bracing (up to 14 hours/day)
- Specific Soft Bracing
- Part-Time Rigid Bracing (15-18 hours/day)
- Full-Time Rigid Bracing (19-22 hours/day)
- Total-Time Rigid Bracing (23-25 hours/day)- Casting
- Surgery

57. Tick the MINIMUM treatment that you would use in ADOLESCENT SCOLIOSIS Risser 2 - Severe degree

- Nothing
- Observation every 36 months
- Observation every 12 months
- Observation every 8 months
- Observation every 6 months
- Observation every 3 months
- Physiotherapeutic Scoliosis-Specific Exercises
- Scoliosis Inpatient Rehabilitation
- Night-Time Rigid Bracing (in bed only)
- Home-Time Rigid Bracing (up to 14 hours/day)
- Specific Soft Bracing
- Part-Time Rigid Bracing (15-18 hours/day)
- Full-Time Rigid Bracing (19-22 hours/day)
- Total-Time Rigid Bracing (23-25 hours/day)- Casting
- Surgery

58. Tick the MOST STRONG treatment that you would use in ADOLESCENT SCOLIOSIS Risser 2 - Severe degree

- ◯ Nothing
- ◯ Observation every 36 months
- ◯ Observation every 12 months
- ◯ Observation every 8 months
- ◯ Observation every 6 months
- ◯ Observation every 3 months
- ◯ Physiotherapeutic Scoliosis-Specific Exercises
- ◯ Scoliosis Inpatient Rehabilitation
- ◯ Night-Time Rigid Bracing (in bed only)
- ◯ Home-Time Rigid Bracing (up to 14 hours/day)
- ◯ Specific Soft Bracing
- ◯ Part-Time Rigid Bracing (15-18 hours/day)
- ◯ Full-Time Rigid Bracing (19-22 hours/day)
- ◯ Total-Time Rigid Bracing (23-25 hours/day)- Casting
- ◯ Surgery

ADOLESCENT IDIOPATHIC SCOLIOSIS - Risser 3

59. Tick the MINIMUM treatment that you would use in ADOLESCENT SCOLIOSIS Risser 3 - Low degree

- Nothing
- Observation every 36 months
- Observation every 12 months
- Observation every 8 months
- Observation every 6 months
- Observation every 3 months
- Physiotherapeutic Scoliosis-Specific Exercises
- Scoliosis Inpatient Rehabilitation
- Night-Time Rigid Bracing (in bed only)
- Home-Time Rigid Bracing (up to 14 hours/day)
- Specific Soft Bracing
- Part-Time Rigid Bracing (15-18 hours/day)
- Full-Time Rigid Bracing (19-22 hours/day)
- Total-Time Rigid Bracing (23-25 hours/day)- Casting
- Surgery

60. Tick the MOST STRONG treatment that you would use in ADOLESCENT SCOLIOSIS Risser 3 - Low degree

- ◯ Nothing
- ◯ Observation every 36 months
- ◯ Observation every 12 months
- ◯ Observation every 8 months
- ◯ Observation every 6 months
- ◯ Observation every 3 months
- ◯ Physiotherapeutic Scoliosis-Specific Exercises
- ◯ Scoliosis Inpatient Rehabilitation
- ◯ Night-Time Rigid Bracing (in bed only)
- ◯ Home-Time Rigid Bracing (up to 14 hours/day)
- ◯ Specific Soft Bracing
- ◯ Part-Time Rigid Bracing (15-18 hours/day)
- ◯ Full-Time Rigid Bracing (19-22 hours/day)
- ◯ Total-Time Rigid Bracing (23-25 hours/day)- Casting
- ◯ Surgery

61. Tick the MINIMUM treatment that you would use in ADOLESCENT SCOLIOSIS Risser 3 - Moderate degree

- ◯ Nothing
- ◯ Observation every 36 months
- ◯ Observation every 12 months
- ◯ Observation every 8 months
- ◯ Observation every 6 months
- ◯ Observation every 3 months
- ◯ Physiotherapeutic Scoliosis-Specific Exercises
- ◯ Scoliosis Inpatient Rehabilitation
- ◯ Night-Time Rigid Bracing (in bed only)
- ◯ Home-Time Rigid Bracing (up to 14 hours/day)
- ◯ Specific Soft Bracing
- ◯ Part-Time Rigid Bracing (15-18 hours/day)
- ◯ Full-Time Rigid Bracing (19-22 hours/day)
- ◯ Total-Time Rigid Bracing (23-25 hours/day)- Casting
- ◯ Surgery

62. Tick the MOST STRONG treatment that you would use in ADOLESCENT SCOLIOSIS Risser 3 - Moderate degree

- Nothing
- Observation every 36 months
- Observation every 12 months
- Observation every 8 months
- Observation every 6 months
- Observation every 3 months
- Physiotherapeutic Scoliosis-Specific Exercises
- Scoliosis Inpatient Rehabilitation
- Night-Time Rigid Bracing (in bed only)
- Home-Time Rigid Bracing (up to 14 hours/day)
- Specific Soft Bracing
- Part-Time Rigid Bracing (15-18 hours/day)
- Full-Time Rigid Bracing (19-22 hours/day)
- Total-Time Rigid Bracing (23-25 hours/day)- Casting
- Surgery

63. Tick the MINIMUM treatment that you would use in ADOLESCENT SCOLIOSIS Risser 3 - Severe degree

- ◯ Nothing
- ◯ Observation every 36 months
- ◯ Observation every 12 months
- ◯ Observation every 8 months
- ◯ Observation every 6 months
- ◯ Observation every 3 months
- ◯ Physiotherapeutic Scoliosis-Specific Exercises
- ◯ Scoliosis Inpatient Rehabilitation
- ◯ Night-Time Rigid Bracing (in bed only)
- ◯ Home-Time Rigid Bracing (up to 14 hours/day)
- ◯ Specific Soft Bracing
- ◯ Part-Time Rigid Bracing (15-18 hours/day)
- ◯ Full-Time Rigid Bracing (19-22 hours/day)
- ◯ Total-Time Rigid Bracing (23-25 hours/day)- Casting
- ◯ Surgery

64. Tick the MOST STRONG treatment that you would use in ADOLESCENT SCOLIOSIS Risser 3 - Severe degree

- ◯ Nothing
- ◯ Observation every 36 months
- ◯ Observation every 12 months
- ◯ Observation every 8 months
- ◯ Observation every 6 months
- ◯ Observation every 3 months
- ◯ Physiotherapeutic Scoliosis-Specific Exercises
- ◯ Scoliosis Inpatient Rehabilitation
- ◯ Night-Time Rigid Bracing (in bed only)
- ◯ Home-Time Rigid Bracing (up to 14 hours/day)
- ◯ Specific Soft Bracing
- ◯ Part-Time Rigid Bracing (15-18 hours/day)
- ◯ Full-Time Rigid Bracing (19-22 hours/day)
- ◯ Total-Time Rigid Bracing (23-25 hours/day)- Casting
- ◯ Surgery

ADOLESCENT IDIOPATHIC SCOLIOSIS - Risser 4

65. Tick the MINIMUM treatment that you would use in ADOLESCENT SCOLIOSIS Risser 4 - Low degree

- Nothing
- Observation every 36 months
- Observation every 12 months
- Observation every 8 months
- Observation every 6 months
- Observation every 3 months
- Physiotherapeutic Scoliosis-Specific Exercises
- Scoliosis Inpatient Rehabilitation
- Night-Time Rigid Bracing (in bed only)
- Home-Time Rigid Bracing (up to 14 hours/day)
- Specific Soft Bracing
- Part-Time Rigid Bracing (15-18 hours/day)
- Full-Time Rigid Bracing (19-22 hours/day)
- Total-Time Rigid Bracing (23-25 hours/day)- Casting
- Surgery

66. Tick the MOST STRONG treatment that you would use in ADOLESCENT SCOLIOSIS Risser 4 - Low degree

- ◯ Nothing
- ◯ Observation every 36 months
- ◯ Observation every 12 months
- ◯ Observation every 8 months
- ◯ Observation every 6 months
- ◯ Observation every 3 months
- ◯ Physiotherapeutic Scoliosis-Specific Exercises
- ◯ Scoliosis Inpatient Rehabilitation
- ◯ Night-Time Rigid Bracing (in bed only)
- ◯ Home-Time Rigid Bracing (up to 14 hours/day)
- ◯ Specific Soft Bracing
- ◯ Part-Time Rigid Bracing (15-18 hours/day)
- ◯ Full-Time Rigid Bracing (19-22 hours/day)
- ◯ Total-Time Rigid Bracing (23-25 hours/day)- Casting
- ◯ Surgery

67. Tick the MINIMUM treatment that you would use in ADOLESCENT SCOLIOSIS Risser 4 - Moderate degree

- Nothing
- Observation every 36 months
- Observation every 12 months
- Observation every 8 months
- Observation every 6 months
- Observation every 3 months
- Physiotherapeutic Scoliosis-Specific Exercises
- Scoliosis Inpatient Rehabilitation
- Night-Time Rigid Bracing (in bed only)
- Home-Time Rigid Bracing (up to 14 hours/day)
- Specific Soft Bracing
- Part-Time Rigid Bracing (15-18 hours/day)
- Full-Time Rigid Bracing (19-22 hours/day)
- Total-Time Rigid Bracing (23-25 hours/day)- Casting
- Surgery

68. Tick the MOST STRONG treatment that you would use in ADOLESCENT SCOLIOSIS Risser 4 - Moderate degree

- Nothing
- Observation every 36 months
- Observation every 12 months
- Observation every 8 months
- Observation every 6 months
- Observation every 3 months
- Physiotherapeutic Scoliosis-Specific Exercises
- Scoliosis Inpatient Rehabilitation
- Night-Time Rigid Bracing (in bed only)
- Home-Time Rigid Bracing (up to 14 hours/day)
- Specific Soft Bracing
- Part-Time Rigid Bracing (15-18 hours/day)
- Full-Time Rigid Bracing (19-22 hours/day)
- Total-Time Rigid Bracing (23-25 hours/day)- Casting
- Surgery

69. Tick the MINIMUM treatment that you would use in ADOLESCENT SCOLIOSIS Risser 4 - Severe degree

- ◯ Nothing
- ◯ Observation every 36 months
- ◯ Observation every 12 months
- ◯ Observation every 8 months
- ◯ Observation every 6 months
- ◯ Observation every 3 months
- ◯ Physiotherapeutic Scoliosis-Specific Exercises
- ◯ Scoliosis Inpatient Rehabilitation
- ◯ Night-Time Rigid Bracing (in bed only)
- ◯ Home-Time Rigid Bracing (up to 14 hours/day)
- ◯ Specific Soft Bracing
- ◯ Part-Time Rigid Bracing (15-18 hours/day)
- ◯ Full-Time Rigid Bracing (19-22 hours/day)
- ◯ Total-Time Rigid Bracing (23-25 hours/day)- Casting
- ◯ Surgery

70. Tick the MOST STRONG treatment that you would use in ADOLESCENT SCOLIOSIS Risser 4 - Severe degree

- Nothing
- Observation every 36 months
- Observation every 12 months
- Observation every 8 months
- Observation every 6 months
- Observation every 3 months
- Physiotherapeutic Scoliosis-Specific Exercises
- Scoliosis Inpatient Rehabilitation
- Night-Time Rigid Bracing (in bed only)
- Home-Time Rigid Bracing (up to 14 hours/day)
- Specific Soft Bracing
- Part-Time Rigid Bracing (15-18 hours/day)
- Full-Time Rigid Bracing (19-22 hours/day)
- Total-Time Rigid Bracing (23-25 hours/day)- Casting
- Surgery

ADULT IDIOPATHIC SCOLIOSIS - up to 25 years of age

71. Tick the MINIMUM treatment that you would use in ADULT SCOLIOSIS up to 25 years of age - Low degree

- ◯ Nothing
- ◯ Observation every 36 months
- ◯ Observation every 12 months
- ◯ Observation every 8 months
- ◯ Observation every 6 months
- ◯ Observation every 3 months
- ◯ Physiotherapeutic Scoliosis-Specific Exercises
- ◯ Scoliosis Inpatient Rehabilitation
- ◯ Night-Time Rigid Bracing (in bed only)
- ◯ Home-Time Rigid Bracing (up to 14 hours/day)
- ◯ Specific Soft Bracing
- ◯ Part-Time Rigid Bracing (15-18 hours/day)
- ◯ Full-Time Rigid Bracing (19-22 hours/day)
- ◯ Total-Time Rigid Bracing (23-25 hours/day)- Casting
- ◯ Surgery

72. Tick the MOST STRONG treatment that you would use in ADULT SCOLIOSIS up to 25 years of age - Low degree

- Nothing
- Observation every 36 months
- Observation every 12 months
- Observation every 8 months
- Observation every 6 months
- Observation every 3 months
- Physiotherapeutic Scoliosis-Specific Exercises
- Scoliosis Inpatient Rehabilitation
- Night-Time Rigid Bracing (in bed only)
- Home-Time Rigid Bracing (up to 14 hours/day)
- Specific Soft Bracing
- Part-Time Rigid Bracing (15-18 hours/day)
- Full-Time Rigid Bracing (19-22 hours/day)
- Total-Time Rigid Bracing (23-25 hours/day)- Casting
- Surgery

73. Tick the MINIMUM treatment that you would use in ADULT SCOLIOSIS up to 25 years of age - Moderate degree

- ◯ Nothing
- ◯ Observation every 36 months
- ◯ Observation every 12 months
- ◯ Observation every 8 months
- ◯ Observation every 6 months
- ◯ Observation every 3 months
- ◯ Physiotherapeutic Scoliosis-Specific Exercises
- ◯ Scoliosis Inpatient Rehabilitation
- ◯ Night-Time Rigid Bracing (in bed only)
- ◯ Home-Time Rigid Bracing (up to 14 hours/day)
- ◯ Specific Soft Bracing
- ◯ Part-Time Rigid Bracing (15-18 hours/day)
- ◯ Full-Time Rigid Bracing (19-22 hours/day)
- ◯ Total-Time Rigid Bracing (23-25 hours/day)- Casting
- ◯ Surgery

74. Tick the MOST STRONG treatment that you would use in ADULT SCOLIOSIS up to 25 years of age - Moderate degree

- Nothing
- Observation every 36 months
- Observation every 12 months
- Observation every 8 months
- Observation every 6 months
- Observation every 3 months
- Physiotherapeutic Scoliosis-Specific Exercises
- Scoliosis Inpatient Rehabilitation
- Night-Time Rigid Bracing (in bed only)
- Home-Time Rigid Bracing (up to 14 hours/day)
- Specific Soft Bracing
- Part-Time Rigid Bracing (15-18 hours/day)
- Full-Time Rigid Bracing (19-22 hours/day)
- Total-Time Rigid Bracing (23-25 hours/day)- Casting
- Surgery

75. Tick the MINIMUM treatment that you would use in ADULT SCOLIOSIS up to 25 years of age - Severe degree

- Nothing
- Observation every 36 months
- Observation every 12 months
- Observation every 8 months
- Observation every 6 months
- Observation every 3 months
- Physiotherapeutic Scoliosis-Specific Exercises
- Scoliosis Inpatient Rehabilitation
- Night-Time Rigid Bracing (in bed only)
- Home-Time Rigid Bracing (up to 14 hours/day)
- Specific Soft Bracing
- Part-Time Rigid Bracing (15-18 hours/day)
- Full-Time Rigid Bracing (19-22 hours/day)
- Total-Time Rigid Bracing (23-25 hours/day)- Casting
- Surgery

76. Tick the MOST STRONG treatment that you would use in ADULT SCOLIOSIS up to 25 years of age - Severe degree

- Nothing
- Observation every 36 months
- Observation every 12 months
- Observation every 8 months
- Observation every 6 months
- Observation every 3 months
- Physiotherapeutic Scoliosis-Specific Exercises
- Scoliosis Inpatient Rehabilitation
- Night-Time Rigid Bracing (in bed only)
- Home-Time Rigid Bracing (up to 14 hours/day)
- Specific Soft Bracing
- Part-Time Rigid Bracing (15-18 hours/day)
- Full-Time Rigid Bracing (19-22 hours/day)
- Total-Time Rigid Bracing (23-25 hours/day)- Casting
- Surgery

ADULT IDIOPATHIC SCOLIOSIS - no pain

77. Tick the MINIMUM treatment that you would use in ADULT SCOLIOSIS no pain - Low degree

- Nothing
- Observation every 36 months
- Observation every 12 months
- Observation every 8 months
- Observation every 6 months
- Observation every 3 months
- Physiotherapeutic Scoliosis-Specific Exercises
- Scoliosis Inpatient Rehabilitation
- Night-Time Rigid Bracing (in bed only)
- Home-Time Rigid Bracing (up to 14 hours/day)
- Specific Soft Bracing
- Part-Time Rigid Bracing (15-18 hours/day)
- Full-Time Rigid Bracing (19-22 hours/day)
- Total-Time Rigid Bracing (23-25 hours/day)- Casting
- Surgery

78. Tick the MOST STRONG treatment that you would use in ADULT SCOLIOSIS no pain - Low degree

- Nothing
- Observation every 36 months
- Observation every 12 months
- Observation every 8 months
- Observation every 6 months
- Observation every 3 months
- Physiotherapeutic Scoliosis-Specific Exercises
- Scoliosis Inpatient Rehabilitation
- Night-Time Rigid Bracing (in bed only)
- Home-Time Rigid Bracing (up to 14 hours/day)
- Specific Soft Bracing
- Part-Time Rigid Bracing (15-18 hours/day)
- Full-Time Rigid Bracing (19-22 hours/day)
- Total-Time Rigid Bracing (23-25 hours/day)- Casting
- Surgery

79. Tick the MINIMUM treatment that you would use in ADULT SCOLIOSIS no pain - Moderate degree

- Nothing
- Observation every 36 months
- Observation every 12 months
- Observation every 8 months
- Observation every 6 months
- Observation every 3 months
- Physiotherapeutic Scoliosis-Specific Exercises
- Scoliosis Inpatient Rehabilitation
- Night-Time Rigid Bracing (in bed only)
- Home-Time Rigid Bracing (up to 14 hours/day)
- Specific Soft Bracing
- Part-Time Rigid Bracing (15-18 hours/day)
- Full-Time Rigid Bracing (19-22 hours/day)
- Total-Time Rigid Bracing (23-25 hours/day)- Casting
- Surgery

80. Tick the MOST STRONG treatment that you would use in ADULT SCOLIOSIS no pain - Moderate degree

- Nothing
- Observation every 36 months
- Observation every 12 months
- Observation every 8 months
- Observation every 6 months
- Observation every 3 months
- Physiotherapeutic Scoliosis-Specific Exercises
- Scoliosis Inpatient Rehabilitation
- Night-Time Rigid Bracing (in bed only)
- Home-Time Rigid Bracing (up to 14 hours/day)
- Specific Soft Bracing
- Part-Time Rigid Bracing (15-18 hours/day)
- Full-Time Rigid Bracing (19-22 hours/day)
- Total-Time Rigid Bracing (23-25 hours/day)- Casting
- Surgery

81. Tick the MINIMUM treatment that you would use in ADULT SCOLIOSIS no pain - Severe degree

- Nothing
- Observation every 36 months
- Observation every 12 months
- Observation every 8 months
- Observation every 6 months
- Observation every 3 months
- Physiotherapeutic Scoliosis-Specific Exercises
- Scoliosis Inpatient Rehabilitation
- Night-Time Rigid Bracing (in bed only)
- Home-Time Rigid Bracing (up to 14 hours/day)
- Specific Soft Bracing
- Part-Time Rigid Bracing (15-18 hours/day)
- Full-Time Rigid Bracing (19-22 hours/day)
- Total-Time Rigid Bracing (23-25 hours/day)- Casting
- Surgery

82. Tick the MOST STRONG treatment that you would use in ADULT SCOLIOSIS no pain - Severe degree

- Nothing
- Observation every 36 months
- Observation every 12 months
- Observation every 8 months
- Observation every 6 months
- Observation every 3 months
- Physiotherapeutic Scoliosis-Specific Exercises
- Scoliosis Inpatient Rehabilitation
- Night-Time Rigid Bracing (in bed only)
- Home-Time Rigid Bracing (up to 14 hours/day)
- Specific Soft Bracing
- Part-Time Rigid Bracing (15-18 hours/day)
- Full-Time Rigid Bracing (19-22 hours/day)
- Total-Time Rigid Bracing (23-25 hours/day)- Casting
- Surgery

ADULT IDIOPATHIC SCOLIOSIS - chronic pain

83. Tick the MINIMUM treatment that you would use in ADULT SCOLIOSIS chronic pain - Low degree curvature

- Nothing
- Observation every 36 months
- Observation every 12 months
- Observation every 8 months
- Observation every 6 months
- Observation every 3 months
- Physiotherapeutic Scoliosis-Specific Exercises
- Scoliosis Inpatient Rehabilitation
- Night-Time Rigid Bracing (in bed only)
- Home-Time Rigid Bracing (up to 14 hours/day)
- Specific Soft Bracing
- Part-Time Rigid Bracing (15-18 hours/day)
- Full-Time Rigid Bracing (19-22 hours/day)
- Total-Time Rigid Bracing (23-25 hours/day)- Casting
- Surgery

84. Tick the MOST STRONG treatment that you would use in ADULT SCOLIOSIS chronic pain - Low degree curvature

- Nothing
- Observation every 36 months
- Observation every 12 months
- Observation every 8 months
- Observation every 6 months
- Observation every 3 months
- Physiotherapeutic Scoliosis-Specific Exercises
- Scoliosis Inpatient Rehabilitation
- Night-Time Rigid Bracing (in bed only)
- Home-Time Rigid Bracing (up to 14 hours/day)
- Specific Soft Bracing
- Part-Time Rigid Bracing (15-18 hours/day)
- Full-Time Rigid Bracing (19-22 hours/day)
- Total-Time Rigid Bracing (23-25 hours/day)- Casting
- Surgery

85. Tick the MINIMUM treatment that you would use in ADULT SCOLIOSIS chronic pain - Moderate degree curvature

- Nothing
- Observation every 36 months
- Observation every 12 months
- Observation every 8 months
- Observation every 6 months
- Observation every 3 months
- Physiotherapeutic Scoliosis-Specific Exercises
- Scoliosis Inpatient Rehabilitation
- Night-Time Rigid Bracing (in bed only)
- Home-Time Rigid Bracing (up to 14 hours/day)
- Specific Soft Bracing
- Part-Time Rigid Bracing (15-18 hours/day)
- Full-Time Rigid Bracing (19-22 hours/day)
- Total-Time Rigid Bracing (23-25 hours/day)- Casting
- Surgery

86. Tick the MOST STRONG treatment that you would use in ADULT SCOLIOSIS chronic pain - Moderate degree curvature

- Nothing
- Observation every 36 months
- Observation every 12 months
- Observation every 8 months
- Observation every 6 months
- Observation every 3 months
- Physiotherapeutic Scoliosis-Specific Exercises
- Scoliosis Inpatient Rehabilitation
- Night-Time Rigid Bracing (in bed only)
- Home-Time Rigid Bracing (up to 14 hours/day)
- Specific Soft Bracing
- Part-Time Rigid Bracing (15-18 hours/day)
- Full-Time Rigid Bracing (19-22 hours/day)
- Total-Time Rigid Bracing (23-25 hours/day)- Casting
- Surgery

87. Tick the MINIMUM treatment that you would use in ADULT SCOLIOSIS chronic pain - Severe degree curvature

- ◯ Nothing
- ◯ Observation every 36 months
- ◯ Observation every 12 months
- ◯ Observation every 8 months
- ◯ Observation every 6 months
- ◯ Observation every 3 months
- ◯ Physiotherapeutic Scoliosis-Specific Exercises
- ◯ Scoliosis Inpatient Rehabilitation
- ◯ Night-Time Rigid Bracing (in bed only)
- ◯ Home-Time Rigid Bracing (up to 14 hours/day)
- ◯ Specific Soft Bracing
- ◯ Part-Time Rigid Bracing (15-18 hours/day)
- ◯ Full-Time Rigid Bracing (19-22 hours/day)
- ◯ Total-Time Rigid Bracing (23-25 hours/day)- Casting
- ◯ Surgery

88. Tick the MOST STRONG treatment that you would use in ADULT SCOLIOSIS chronic pain - Severe degree curvature

- ◯ Nothing
- ◯ Observation every 36 months
- ◯ Observation every 12 months
- ◯ Observation every 8 months
- ◯ Observation every 6 months
- ◯ Observation every 3 months
- ◯ Physiotherapeutic Scoliosis-Specific Exercises
- ◯ Scoliosis Inpatient Rehabilitation
- ◯ Night-Time Rigid Bracing (in bed only)
- ◯ Home-Time Rigid Bracing (up to 14 hours/day)
- ◯ Specific Soft Bracing
- ◯ Part-Time Rigid Bracing (15-18 hours/day)
- ◯ Full-Time Rigid Bracing (19-22 hours/day)
- ◯ Total-Time Rigid Bracing (23-25 hours/day)- Casting
- ◯ Surgery

ELDERLY IDIOPATHIC SCOLIOSIS - no pain

89. Tick the MINIMUM treatment that you would use in ELDERLY SCOLIOSIS no pain - Low degree

- Nothing
- Observation every 36 months
- Observation every 12 months
- Observation every 8 months
- Observation every 6 months
- Observation every 3 months
- Physiotherapeutic Scoliosis-Specific Exercises
- Scoliosis Inpatient Rehabilitation
- Night-Time Rigid Bracing (in bed only)
- Home-Time Rigid Bracing (up to 14 hours/day)
- Specific Soft Bracing
- Part-Time Rigid Bracing (15-18 hours/day)
- Full-Time Rigid Bracing (19-22 hours/day)
- Total-Time Rigid Bracing (23-25 hours/day)- Casting
- Surgery

90. Tick the MOST STRONG treatment that you would use in ELDERLY SCOLIOSIS no pain - Low degree

- Nothing
- Observation every 36 months
- Observation every 12 months
- Observation every 8 months
- Observation every 6 months
- Observation every 3 months
- Physiotherapeutic Scoliosis-Specific Exercises
- Scoliosis Inpatient Rehabilitation
- Night-Time Rigid Bracing (in bed only)
- Home-Time Rigid Bracing (up to 14 hours/day)
- Specific Soft Bracing
- Part-Time Rigid Bracing (15-18 hours/day)
- Full-Time Rigid Bracing (19-22 hours/day)
- Total-Time Rigid Bracing (23-25 hours/day)- Casting
- Surgery

91. Tick the MINIMUM treatment that you would use in ELDERLY SCOLIOSIS no pain - Moderate degree

- ◯ Nothing
- ◯ Observation every 36 months
- ◯ Observation every 12 months
- ◯ Observation every 8 months
- ◯ Observation every 6 months
- ◯ Observation every 3 months
- ◯ Physiotherapeutic Scoliosis-Specific Exercises
- ◯ Scoliosis Inpatient Rehabilitation
- ◯ Night-Time Rigid Bracing (in bed only)
- ◯ Home-Time Rigid Bracing (up to 14 hours/day)
- ◯ Specific Soft Bracing
- ◯ Part-Time Rigid Bracing (15-18 hours/day)
- ◯ Full-Time Rigid Bracing (19-22 hours/day)
- ◯ Total-Time Rigid Bracing (23-25 hours/day)- Casting
- ◯ Surgery

92. Tick the MOST STRONG treatment that you would use in ELDERLY SCOLIOSIS no pain - Moderate degree

- Nothing
- Observation every 36 months
- Observation every 12 months
- Observation every 8 months
- Observation every 6 months
- Observation every 3 months
- Physiotherapeutic Scoliosis-Specific Exercises
- Scoliosis Inpatient Rehabilitation
- Night-Time Rigid Bracing (in bed only)
- Home-Time Rigid Bracing (up to 14 hours/day)
- Specific Soft Bracing
- Part-Time Rigid Bracing (15-18 hours/day)
- Full-Time Rigid Bracing (19-22 hours/day)
- Total-Time Rigid Bracing (23-25 hours/day)- Casting
- Surgery

93. Tick the MINIMUM treatment that you would use in ELDERLY SCOLIOSIS no pain - Severe degree

- Nothing
- Observation every 36 months
- Observation every 12 months
- Observation every 8 months
- Observation every 6 months
- Observation every 3 months
- Physiotherapeutic Scoliosis-Specific Exercises
- Scoliosis Inpatient Rehabilitation
- Night-Time Rigid Bracing (in bed only)
- Home-Time Rigid Bracing (up to 14 hours/day)
- Specific Soft Bracing
- Part-Time Rigid Bracing (15-18 hours/day)
- Full-Time Rigid Bracing (19-22 hours/day)
- Total-Time Rigid Bracing (23-25 hours/day)- Casting
- Surgery

94. Tick the MOST STRONG treatment that you would use in ELDERLY SCOLIOSIS no pain - Severe degree

- Nothing
- Observation every 36 months
- Observation every 12 months
- Observation every 8 months
- Observation every 6 months
- Observation every 3 months
- Physiotherapeutic Scoliosis-Specific Exercises
- Scoliosis Inpatient Rehabilitation
- Night-Time Rigid Bracing (in bed only)
- Home-Time Rigid Bracing (up to 14 hours/day)
- Specific Soft Bracing
- Part-Time Rigid Bracing (15-18 hours/day)
- Full-Time Rigid Bracing (19-22 hours/day)
- Total-Time Rigid Bracing (23-25 hours/day)- Casting
- Surgery

ELDERLY IDIOPATHIC SCOLIOSIS - chronic pain

95. Tick the MINIMUM treatment that you would use in ELDERLY SCOLIOSIS chronic pain - Low degree curvature

- Nothing
- Observation every 36 months
- Observation every 12 months
- Observation every 8 months
- Observation every 6 months
- Observation every 3 months
- Physiotherapeutic Scoliosis-Specific Exercises
- Scoliosis Inpatient Rehabilitation
- Night-Time Rigid Bracing (in bed only)
- Home-Time Rigid Bracing (up to 14 hours/day)
- Specific Soft Bracing
- Part-Time Rigid Bracing (15-18 hours/day)
- Full-Time Rigid Bracing (19-22 hours/day)
- Total-Time Rigid Bracing (23-25 hours/day)- Casting
- Surgery

96. Tick the MOST STRONG treatment that you would use in ELDERLY SCOLIOSIS chronic pain - Low degree curvature

- Nothing
- Observation every 36 months
- Observation every 12 months
- Observation every 8 months
- Observation every 6 months
- Observation every 3 months
- Physiotherapeutic Scoliosis-Specific Exercises
- Scoliosis Inpatient Rehabilitation
- Night-Time Rigid Bracing (in bed only)
- Home-Time Rigid Bracing (up to 14 hours/day)
- Specific Soft Bracing
- Part-Time Rigid Bracing (15-18 hours/day)
- Full-Time Rigid Bracing (19-22 hours/day)
- Total-Time Rigid Bracing (23-25 hours/day)- Casting
- Surgery

97. Tick the MINIMUM treatment that you would use in ELDERLY SCOLIOSIS chronic pain - Moderate degree curvature

- ◯ Nothing
- ◯ Observation every 36 months
- ◯ Observation every 12 months
- ◯ Observation every 8 months
- ◯ Observation every 6 months
- ◯ Observation every 3 months
- ◯ Physiotherapeutic Scoliosis-Specific Exercises
- ◯ Scoliosis Inpatient Rehabilitation
- ◯ Night-Time Rigid Bracing (in bed only)
- ◯ Home-Time Rigid Bracing (up to 14 hours/day)
- ◯ Specific Soft Bracing
- ◯ Part-Time Rigid Bracing (15-18 hours/day)
- ◯ Full-Time Rigid Bracing (19-22 hours/day)
- ◯ Total-Time Rigid Bracing (23-25 hours/day)- Casting
- ◯ Surgery

98. Tick the MOST STRONG treatment that you would use in ELDERLY SCOLIOSIS chronic pain - Moderate degree curvature

- ◯ Nothing
- ◯ Observation every 36 months
- ◯ Observation every 12 months
- ◯ Observation every 8 months
- ◯ Observation every 6 months
- ◯ Observation every 3 months
- ◯ Physiotherapeutic Scoliosis-Specific Exercises
- ◯ Scoliosis Inpatient Rehabilitation
- ◯ Night-Time Rigid Bracing (in bed only)
- ◯ Home-Time Rigid Bracing (up to 14 hours/day)
- ◯ Specific Soft Bracing
- ◯ Part-Time Rigid Bracing (15-18 hours/day)
- ◯ Full-Time Rigid Bracing (19-22 hours/day)
- ◯ Total-Time Rigid Bracing (23-25 hours/day)- Casting
- ◯ Surgery

99. Tick the MINIMUM treatment that you would use in ELDERLY SCOLIOSIS chronic pain - Severe degree curvature

- Nothing
- Observation every 36 months
- Observation every 12 months
- Observation every 8 months
- Observation every 6 months
- Observation every 3 months
- Physiotherapeutic Scoliosis-Specific Exercises
- Scoliosis Inpatient Rehabilitation
- Night-Time Rigid Bracing (in bed only)
- Home-Time Rigid Bracing (up to 14 hours/day)
- Specific Soft Bracing
- Part-Time Rigid Bracing (15-18 hours/day)
- Full-Time Rigid Bracing (19-22 hours/day)
- Total-Time Rigid Bracing (23-25 hours/day)- Casting
- Surgery

100. Tick the MOST STRONG treatment that you would use in ELDERLY SCOLIOSIS chronic pain - Severe degree curvature

- Nothing
- Observation every 36 months
- Observation every 12 months
- Observation every 8 months
- Observation every 6 months
- Observation every 3 months
- Physiotherapeutic Scoliosis-Specific Exercises
- Scoliosis Inpatient Rehabilitation
- Night-Time Rigid Bracing (in bed only)
- Home-Time Rigid Bracing (up to 14 hours/day)
- Specific Soft Bracing
- Part-Time Rigid Bracing (15-18 hours/day)
- Full-Time Rigid Bracing (19-22 hours/day)
- Total-Time Rigid Bracing (23-25 hours/day)- Casting
- Surgery

ELDERLY IDIOPATHIC SCOLIOSIS - trunk decompensation

101. Tick the MINIMUM treatment that you would use in ELDERLY SCOLIOSIS trunk decompensation - Low degree curvature

- ○ Nothing
- ○ Observation every 36 months
- ○ Observation every 12 months
- ○ Observation every 8 months
- ○ Observation every 6 months
- ○ Observation every 3 months
- ○ Physiotherapeutic Scoliosis-Specific Exercises
- ○ Scoliosis Inpatient Rehabilitation
- ○ Night-Time Rigid Bracing (in bed only)
- ○ Home-Time Rigid Bracing (up to 14 hours/day)
- ○ Specific Soft Bracing
- ○ Part-Time Rigid Bracing (15-18 hours/day)
- ○ Full-Time Rigid Bracing (19-22 hours/day)
- ○ Total-Time Rigid Bracing (23-25 hours/day)- Casting
- ○ Surgery

102. Tick the MOST STRONG treatment that you would use in ELDERLY SCOLIOSIS trunk decompensation - Low degree curvature

- ◯ Nothing
- ◯ Observation every 36 months
- ◯ Observation every 12 months
- ◯ Observation every 8 months
- ◯ Observation every 6 months
- ◯ Observation every 3 months
- ◯ Physiotherapeutic Scoliosis-Specific Exercises
- ◯ Scoliosis Inpatient Rehabilitation
- ◯ Night-Time Rigid Bracing (in bed only)
- ◯ Home-Time Rigid Bracing (up to 14 hours/day)
- ◯ Specific Soft Bracing
- ◯ Part-Time Rigid Bracing (15-18 hours/day)
- ◯ Full-Time Rigid Bracing (19-22 hours/day)
- ◯ Total-Time Rigid Bracing (23-25 hours/day)- Casting
- ◯ Surgery

103. Tick the MINIMUM treatment that you would use in ELDERLY SCOLIOSIS trunk decompensation - Moderate degree curvature

- ◯ Nothing
- ◯ Observation every 36 months
- ◯ Observation every 12 months
- ◯ Observation every 8 months
- ◯ Observation every 6 months
- ◯ Observation every 3 months
- ◯ Physiotherapeutic Scoliosis-Specific Exercises
- ◯ Scoliosis Inpatient Rehabilitation
- ◯ Night-Time Rigid Bracing (in bed only)
- ◯ Home-Time Rigid Bracing (up to 14 hours/day)
- ◯ Specific Soft Bracing
- ◯ Part-Time Rigid Bracing (15-18 hours/day)
- ◯ Full-Time Rigid Bracing (19-22 hours/day)
- ◯ Total-Time Rigid Bracing (23-25 hours/day)- Casting
- ◯ Surgery

104. Tick the MOST STRONG treatment that you would use in ELDERLY SCOLIOSIS trunk decompensation - Moderate degree curvature

- Nothing
- Observation every 36 months
- Observation every 12 months
- Observation every 8 months
- Observation every 6 months
- Observation every 3 months
- Physiotherapeutic Scoliosis-Specific Exercises
- Scoliosis Inpatient Rehabilitation
- Night-Time Rigid Bracing (in bed only)
- Home-Time Rigid Bracing (up to 14 hours/day)
- Specific Soft Bracing
- Part-Time Rigid Bracing (15-18 hours/day)
- Full-Time Rigid Bracing (19-22 hours/day)
- Total-Time Rigid Bracing (23-25 hours/day)- Casting
- Surgery

105. Tick the MINIMUM treatment that you would use in ELDERLY SCOLIOSIS trunk decompensation - Severe degree curvature

- Nothing
- Observation every 36 months
- Observation every 12 months
- Observation every 8 months
- Observation every 6 months
- Observation every 3 months
- Physiotherapeutic Scoliosis-Specific Exercises
- Scoliosis Inpatient Rehabilitation
- Night-Time Rigid Bracing (in bed only)
- Home-Time Rigid Bracing (up to 14 hours/day)
- Specific Soft Bracing
- Part-Time Rigid Bracing (15-18 hours/day)
- Full-Time Rigid Bracing (19-22 hours/day)
- Total-Time Rigid Bracing (23-25 hours/day)- Casting
- Surgery

106. Tick the MOST STRONG treatment that you would use in ELDERLY SCOLIOSIS trunk decompensation - Severe degree curvature

- Nothing
- Observation every 36 months
- Observation every 12 months
- Observation every 8 months
- Observation every 6 months
- Observation every 3 months
- Physiotherapeutic Scoliosis-Specific Exercises
- Scoliosis Inpatient Rehabilitation
- Night-Time Rigid Bracing (in bed only)
- Home-Time Rigid Bracing (up to 14 hours/day)
- Specific Soft Bracing
- Part-Time Rigid Bracing (15-18 hours/day)
- Full-Time Rigid Bracing (19-22 hours/day)
- Total-Time Rigid Bracing (23-25 hours/day)- Casting
- Surgery
