# Supplementary material for: 2016 SOSORT guidelines: orthopaedic and rehabilitation treatment of idiopathic scoliosis during growth
Source: Scoliosis Spinal Disord. 2018 Jan 10;13:3. doi: 10.1186/s13013-017-0145-8 (PMC5795289; doi:10.1186/s13013-017-0145-8)
Supplement: Supplementary file 6 — Questionnaires used for Dephi procedure, from Round 1 to round 3. (PDF 1270 kb) [file 13013_2017_145_MOESM6_ESM.pdf]

2016 recommendations - third round

Welcome to the Consensus on Recommendations for the 2016 SOSORT Guidelines

Dear colleagues

this Consensus process is part of the development of the 2016 SOSORT Guidelines that will adjourne and substitute the 2011 Guidelines (http://scoliosisjournal.biomedcentral.com/articles/10.1186/1748-7161-7-3) and will be published in our official journal Scoliosis and Spinal Deformity.

After the previous Consensus on classifications and treatments, it is now the time to reach a Consensus on the recommendations. You will have to approve or reject the recommendations, and rate their importance. Moreover you can still give suggestions for other recommendations, and or to change and improve the actual version of the recommendations.

Please fill in accurately this questionnaire so to make our Guideliens stronger and really representative of our community of expert in conservative orthopedic and rehabilitation scoliosis treatment.

IMPORTANT NOTE. When there is some evidence on the recommendation, that has been specified using LoE (Level of Evidence) with a number as follow:
- I : Multiple Randomized Controlled Trials or Systematic Reviews of such studies
- II: One Randomized Controlled Trial
- III: Multiple Controlled nonrandomized Studies or Systematic Reviews of such studies
- IV: Other studies
Other two Levels will be defined according to this Consensus results
- V: SOSORT Consensus with more than 90% of agreement
- VI: SOSORT Consensus with 70 to 89% of agreement

When you do not find LoE number, that means that it will be defined after this Consensus.

When there is a literature LoE on a specific recommendation, by definition what can be done is only a rephrasing the recommendation but it cannot be simply rejected.

PLEASE CONSIDER THAT EACH RECOMMENDATION MUST BE A SINGLE STATEMENT. WE CANNOT GIVE TOO MUCH DETAILS (eg Cobb degree limits or other adjunctive treatments) UNLESS ABSOLUTELY NECESSARY. DETAILS WILL BE GIVEN OR WITH OTHER RECOMMENDATIONS OR IN THE TEXT OF THE GUIDELINES

Thank you for participating in our Consensus. Your feedback is important.

2016 recommendations - third round

1. What is your family name ?

2. What is your first name?

3. What is your gender?

- Female
- Male

4. What is your age?

- 18 to 24
- 25 to 34
- 35 to 44
- 45 to 54
- 55 to 64
- 65 to 74
- 75 or older

5. What is your profession ?

- MD, orthopedic surgeon
- MD, physical and rehabilitation medicine
- MD, others
- Physiotherapist
- Orthotist
- PhD
- Other (please specify)

6. In what country do you work?

2016 recommendations - third round

Bracing

Bracing is recommended to treat adolescent idiopathic scoliosis
LoE: I

7. Do you agree with this recommendation ?

- ◯ No
- ◯ Yes
- ◯ Yes, with suggestions

8. What is the strength of this recommendation ?

- ◯ it must be applied widely and to all patients with this specific need
- ◯ it is important, but can be applied not to all patients with this specific need
- ◯ less important, it can be applied on a voluntary basis
- ◯ very low importance

2016 recommendations - third round

Bracing

Bracing is recommended to treat juvenile and infantile idiopathic scoliosis as the first step in an attempt to avoid or at least postpone surgery to a more adequate age

9. Do you agree with this recommendation ?

- ◯ No
- ◯ Yes
- ◯ Yes, with suggestions

10. What is the strength of this recommendation ?

- ◯ it must be applied widely and to all patients with this specific need
- ◯ it is important, but can be applied not to all patients with this specific need
- ◯ less important, it can be applied on a voluntary basis
- ◯ very low importance

2016 recommendations - third round

Bracing

Casting (or very rigid bracing) is recommended to treat infantile idiopathic scoliosis to try stabilizing the curve

11. Do you agree to consider very rigid bracing effective as casting in infantile idiopathic scoliosis ?

- ◯ Yes: leave the recommendation as it is
- ◯ No, but you can leave the recommendation as it is
- ◯ No, and you should change the recommendation accordingly

12. Do you agree with this recommendation ?

- ◯ No
- ◯ Yes
- ◯ Yes, with suggestions

13. What is the strength of this recommendation ?

- ◯ it must be applied widely and to all patients with this specific need
- ◯ it is important, but can be applied not to all patients with this specific need
- ◯ less important, it can be applied on a voluntary basis
- ◯ very low importance

2016 recommendations - third round

Bracing

It is recommended not to apply bracing to treat patients with curves below 15±5° Cobb, unless otherwise justified in the opinion of a clinician specialized in conservative treatment of spinal deformities

14. Do you agree with this recommendation ?

- ◯ No
- ◯ Yes
- ◯ Yes, with suggestions

15. What is the strength of this recommendation ?

- ◯ it must be applied widely and to all patients with this specific need
- ◯ it is important, but can be applied not to all patients with this specific need
- ◯ less important, it can be applied on a voluntary basis
- ◯ very low importance

2016 recommendations - third round

Bracing

Bracing is recommended to treat patients with curves above 20±5° Cobb, still growing, and demonstrated progression of deformity or elevated risk of worsening, unless otherwise justified in the opinion of a clinician specialized in conservative treatment of spinal deformities
LoE I

16. Do you agree with this recommendation ?

- ◯ No
- ◯ Yes
- ◯ Yes, with suggestions

17. What is the strength of this recommendation ?

- ◯ it must be applied widely and to all patients with this specific need
- ◯ it is important, but can be applied not to all patients with this specific need
- ◯ less important, it can be applied on a voluntary basis
- ◯ very low importance

2016 recommendations - third round

Bracing

Very hard rigid bracing (casting) is recommended to treat patients with curves between 45° and 60° to try avoiding surgery
LoE IV

18. Do you agree with this recommendation ?

- ◯ No
- ◯ Yes
- ◯ Yes, with suggestions

19. What is the strength of this recommendation ?

- ◯ it must be applied widely and to all patients with this specific need
- ◯ it is important, but can be applied not to all patients with this specific need
- ◯ less important, it can be applied on a voluntary basis
- ◯ very low importance

2016 recommendations - third round

Bracing

It is recommended that each treating team provide the brace that they have the most experience and expertise with and are most prepared to manage; due to lack of comparative evidence there is no brace that can be recommended over the others.

20. Do you agree with this recommendation ?

- ◯ No
- ◯ Yes
- ◯ Yes, with suggestions

21. What is the strength of this recommendation ?

- ◯ it must be applied widely and to all patients with this specific need
- ◯ it is important, but can be applied not to all patients with this specific need
- ◯ less important, it can be applied on a voluntary basis
- ◯ very low importance

2016 recommendations - third round

Bracing

It is recommended that braces are worn full time or no less than 18 hours per day at the beginning of treatment, unless otherwise justified in the opinion of a clinician specialized in conservative treatment of spinal deformities
LoE: II

22. Do you agree with this recommendation ?

- ○ No
- ○ Yes
- ○ Yes, with suggestions

23. What is the strength of this recommendation ?

- ○ it must be applied widely and to all patients with this specific need
- ○ it is important, but can be applied not to all patients with this specific need
- ○ less important, it can be applied on a voluntary basis
- ○ very low importance

Bracing

It is recommended that daily brace wear is proportionate to the deformity severity, age of patient, scoliosis stage, aim and overall results of treatment, and the expected compliance
LoE: II

24. Do you agree with this recommendation ?

- ◯ No
- ◯ Yes
- ◯ Yes, with suggestions

25. What is the strength of this recommendation ?

- ◯ it must be applied widely and to all patients with this specific need
- ◯ it is important, but can be applied not to all patients with this specific need
- ◯ less important, it can be applied on a voluntary basis
- ◯ very low importance

2016 recommendations - third round

Bracing

It is recommended that braces are worn until the end of vertebral bone growth, and then the wearing time is gradually reduced until full bone maturity is reached, unless otherwise justified in the opinion of a clinician specialized in conservative treatment of spinal deformities

26. Do you agree with this recommendation ?

- ◯ No
- ◯ Yes
- ◯ Yes, with suggestions

27. What is the strength of this recommendation ?

- ◯ it must be applied widely and to all patients with this specific need
- ◯ it is important, but can be applied not to all patients with this specific need
- ◯ less important, it can be applied on a voluntary basis
- ◯ very low importance

2016 recommendations - third round

Bracing

It is recommended that the wearing time of the brace is gradually reduced, while performing stabilizing exercises, to allow adaptation of the postural system and maintain results

28. Do you agree with this recommendation ?

- No
- Yes
- Yes, with suggestions

29. What is the strength of this recommendation ?

- it must be applied widely and to all patients with this specific need
- it is important, but can be applied not to all patients with this specific need
- less important, it can be applied on a voluntary basis
- very low importance

2016 recommendations - third round

Bracing

It is recommended that any mean is used to encourage compliance, including a careful adherence to the recommendations defined in the SOSORT Guidelines for Bracing Management

30. Do you agree with this recommendation ?

- No
- Yes
- Yes, with suggestions

31. What is the strength of this recommendation ?

- it must be applied widely and to all patients with this specific need
- it is important, but can be applied not to all patients with this specific need
- less important, it can be applied on a voluntary basis
- very low importance

2016 recommendations - third round

Bracing

It is recommended that compliance to bracing is regularly checked through compliance monitor devices

32. Do you agree with this recommendation ?

- ◯ No
- ◯ Yes
- ◯ Yes, with suggestions

33. What is the strength of this recommendation ?

- ◯ it must be applied widely and to all patients with this specific need
- ◯ it is important, but can be applied not to all patients with this specific need
- ◯ less important, it can be applied on a voluntary basis
- ◯ very low importance

2016 recommendations - third round

Bracing

It is recommended that the effectiveness of each brace is evaluated with an in-brace x-ray, and improvements be made if warranted

34. Do you agree with this recommendation ?

- ○ No
- ○ Yes
- ○ Yes, with suggestions

35. What is the strength of this recommendation ?

- ○ it must be applied widely and to all patients with this specific need
- ○ it is important, but can be applied not to all patients with this specific need
- ○ less important, it can be applied on a voluntary basis
- ○ very low importance

2016 recommendations - third round

Bracing

It is recommended that the prescribing physician and the constructing orthotist are experts according to the criteria defined in the SOSORT Guidelines for Bracing Management

36. Do you agree with this recommendation ?

- No
- Yes
- Yes, with suggestions

37. What is the strength of this recommendation ?

- it must be applied widely and to all patients with this specific need
- it is important, but can be applied not to all patients with this specific need
- less important, it can be applied on a voluntary basis
- very low importance

Bracing

It is recommended that bracing is applied by a scoliosis expert therapeutic multidisciplinary team, including a physician, an orthotist and a therapist, according to the criteria defined in the SOSORT Guidelines for Bracing Management

38. Do you agree with this recommendation ?

- No
- Yes
- Yes, with suggestions

39. What is the strength of this recommendation ?

- it must be applied widely and to all patients with this specific need
- it is important, but can be applied not to all patients with this specific need
- less important, it can be applied on a voluntary basis
- very low importance

2016 recommendations - third round

Bracing

It is recommended that the criteria defined in the SOSORT Guidelines for Bracing Management are carefully followed throughout brace construction (prescription, construction, check, correction, follow-up) for each brace

40. Do you agree with this recommendation ?

- No
- Yes
- Yes, with suggestions

41. What is the strength of this recommendation ?

- it must be applied widely and to all patients with this specific need
- it is important, but can be applied not to all patients with this specific need
- less important, it can be applied on a voluntary basis
- very low importance

2016 recommendations - third round

Bracing

It is recommended that the brace is specifically designed for the type of the curve to be treated

42. Do you agree with this recommendation ?

- No
- Yes
- Yes, with suggestions

43. What is the strength of this recommendation ?

- it must be applied widely and to all patients with this specific need
- it is important, but can be applied not to all patients with this specific need
- less important, it can be applied on a voluntary basis
- very low importance

2016 recommendations - third round

Bracing

It is recommended that the brace prescribed to treat a scoliotic deformity takes into account all the three planes: frontal, horizontal and sagittal

44. Do you agree with this recommendation ?

- No
- Yes
- Yes, with suggestions

45. What is the strength of this recommendation ?

- it must be applied widely and to all patients with this specific need
- it is important, but can be applied not to all patients with this specific need
- less important, it can be applied on a voluntary basis
- very low importance

2016 recommendations - third round

Bracing

It is recommended to use the least invasive brace in relation to the clinical situation, provided the same effectiveness, to reduce the psychological impact and to ensure better patient compliance

46. Do you agree with this recommendation ?

- ◯ No
- ◯ Yes
- ◯ Yes, with suggestion

47. What is the strength of this recommendation ?

- ◯ it must be applied widely and to all patients with this specific need
- ◯ it is important, but can be applied not to all patients with this specific need
- ◯ less important, it can be applied on a voluntary basis
- ◯ very low importance

2016 recommendations - third round

Bracing

It is recommended that braces do not restrict thorax excursion in a way that reduces respiratory function

48. Do you agree with this recommendation ?

- ◯ No
- ◯ Yes
- ◯ Yes, with suggestions

49. What is the strength of this recommendation ?

- ◯ it must be applied widely and to all patients with this specific need
- ◯ it is important, but can be applied not to all patients with this specific need
- ◯ less important, it can be applied on a voluntary basis
- ◯ very low importance

2016 recommendations - third round

Bracing

It is recommended that braces are prescribed, constructed and fitted in an out-patient setting

50. Do you agree with this recommendation ?

- No
- Yes
- Yes, with suggestions

51. What is the strength of this recommendation ?

- it must be applied widely and to all patients with this specific need
- it is important, but can be applied not to all patients with this specific need
- less important, it can be applied on a voluntary basis
- very low importance

2016 recommendations - third round

Bracing

It is recommended that braces are regularly changed according to growth and/or specific pathological needs as judged by a scoliosis expert physician

52. Do you agree with this recommendation ?

- ◯ No
- ◯ Yes
- ◯ Yes, with suggestions

53. What is the strength of this recommendation ?

- ◯ it must be applied widely and to all patients with this specific need
- ◯ it is important, but can be applied not to all patients with this specific need
- ◯ less important, it can be applied on a voluntary basis
- ◯ very low importance

2016 recommendations - third round

Bracing

It is recommended that out of brace x-rays are regularly performed to check the effectiveness of bracing treatment: the number of hours out of brace before x-ray taking should correspond to the daily weaning time

54. Do you agree with this recommendation ?

- No
- Yes
- Yes, with suggestions

55. What is the strength of this recommendation ?

- it must be applied widely and to all patients with this specific need
- it is important, but can be applied not to all patients with this specific need
- less important, it can be applied on a voluntary basis
- very low importance

2016 recommendations - third round

Bracing

It is recommended that brace is definitively weaned when bone maturity is reached and results are checked with an x--ray after 48 hours without wearing the brace

56. Do you agree with this recommendation ?

- No
- Yes
- Yes, with suggestions

57. What is the strength of this recommendation ?

- it must be applied widely and to all patients with this specific need
- it is important, but can be applied not to all patients with this specific need
- less important, it can be applied on a voluntary basis
- very low importance

2016 recommendations - third round

Bracing

It is recommended that scoliosis specific exercises are prescribed to patients wearing braces

58. Do you agree with this recommendation ?

- ◯ No
- ◯ Yes
- ◯ Yes, with suggestions

59. What is the strength of this recommendation ?

- ◯ it must be applied widely and to all patients with this specific need
- ◯ it is important, but can be applied not to all patients with this specific need
- ◯ less important, it can be applied on a voluntary basis
- ◯ very low importance

2016 recommendations - third round

Physiotherapeutic Scoliosis Specific Exercises

Physiotherapeutic Scoliosis Specific Exercises are recommended as the first step to treat idiopathic scoliosis patients to prevent/limit progression of the deformity and bracing
LoE: I

60. Do you agree with this recommendation ?

- ○ No
- ○ Yes
- ○ Yes, with suggestions

61. What is the strength of this recommendation ?

- ○ it must be applied widely and to all patients with this specific need
- ○ it is important, but can be applied not to all patients with this specific need
- ○ less important, it can be applied on a voluntary basis
- ○ very low importance

2016 recommendations - third round

Physiotherapeutic Scoliosis Specific Exercises

It is recommended that Physiotherapeutic Scoliosis Specific Exercises follow SOSORT Consensus and are based on auto-correction in 3D, training in ADL, stabilizing the corrected posture, and patient education
LoE: II

62. Do you agree with this recommendation ?

- ◯ No
- ◯ Yes
- ◯ Yes, with suggestons

63. What is the strength of this recommendation ?

- ◯ it must be applied widely and to all patients with this specific need
- ◯ it is important, but can be applied not to all patients with this specific need
- ◯ less important, it can be applied on a voluntary basis
- ◯ very low importance

2016 recommendations - third round

Physiotherapeutic Scoliosis Specific Exercises

It is recommended that Physiotherapeutic Scoliosis Specific Exercises follow one of the Schools that have shown the effectiveness of their approach with scientific studies
LoE: III

64. Do you agree with this recommendation ?

- No
- Yes
- Yes, with suggestons

65. What is the strength of this recommendation ?

- it must be applied widely and to all patients with this specific need
- it is important, but can be applied not to all patients with this specific need
- less important, it can be applied on a voluntary basis
- very low importance

2016 recommendations - third round

Physiotherapeutic Scoliosis Specific Exercises

It is recommended that Physiotherapeutic Scoliosis Specific Exercise programs are designed and applied by therapists specifically trained in Scoliosis Specific Exercises

66. Do you agree with this recommendation ?

- ◯ No
- ◯ Yes
- ◯ Yes, with the suggestions

67. What is the strength of this recommendation ?

- ◯ it must be applied widely and to all patients with this specific need
- ◯ it is important, but can be applied not to all patients with this specific need
- ◯ less important, it can be applied on a voluntary basis
- ◯ very low importance

2016 recommendations - third round

Physiotherapeutic Scoliosis Specific Exercises

It is recommended that Physiotherapeutic Scoliosis Specific Exercises are proposed by therapists included in scoliosis treatment teams, with close cooperation between all members

68. Do you agree with this recommendation ?

- No
- Yes
- Yes, with suggestions

69. What is the strength of this recommendation ?

- it must be applied widely and to all patients with this specific need
- it is important, but can be applied not to all patients with this specific need
- less important, it can be applied on a voluntary basis
- very low importance

2016 recommendations - third round

Physiotherapeutic Scoliosis Specific Exercises

It is recommended that Physiotherapeutic Scoliosis Specific Exercises are individualized according to other treatments in place, assessment of patient, curve pattern and size, risk of progression, treatment phase, patients needs and family considerations

70. Do you agree with this recommendation ?

- ○ No
- ○ Yes
- ○ Yes, with suggestions

71. What is the strength of this recommendation ?

- ○ it must be applied widely and to all patients with this specific need
- ○ it is important, but can be applied not to all patients with this specific need
- ○ less important, it can be applied on a voluntary basis
- ○ very low importance

2016 recommendations - third round

Physiotherapeutic Scoliosis Specific Exercises

It is recommended that Physiotherapeutic Scoliosis Specific Exercises are always individualized even if performed in small groups

72. Do you agree with this recommendation ?

- ◯ No
- ◯ Yes
- ◯ Yes, with suggestions

73. What is the strength of this recommendation ?

- ◯ it must be applied widely and to all patients with this specific need
- ◯ it is important, but can be applied not to all patients with this specific need
- ◯ less important, it can be applied on a voluntary basis
- ◯ very low importance

2016 recommendations - third round

Physiotherapeutic Scoliosis Specific Exercises

It is recommended that Physiotherapeutic Scoliosis Specific Exercises are performed regularly throughout treatment to achieve best results

74. Do you agree with this recommendation ?

- No
- Yes
- Yes, with suggestions

75. What is the strength of this recommendation ?

- it must be applied widely and to all patients with this specific need
- it is important, but can be applied not to all patients with this specific need
- less important, it can be applied on a voluntary basis
- very low importance

2016 recommendations - third round

Physiotherapeutic Scoliosis Specific Exercises

It is recommended that therapists implement a compliance system for exercise tracking

76. Do you agree with this recommendation ?

- ◯ No
- ◯ Yes
- ◯ Yes, with suggestions

77. What is the strength of this recommendation ?

- ◯ it must be applied widely and to all patients with this specific need
- ◯ it is important, but can be applied not to all patients with this specific need
- ◯ less important, it can be applied on a voluntary basis
- ◯ very low importance

2016 recommendations - third round

Physiotherapeutic Scoliosis Specific Exercises

It is recommended that therapists regularly assess patients quality of Physiotherapeutic Scoliosis Specific Exercises performed by the patients

78. Do you agree with this recommendation ?

- ◯ No
- ◯ Yes
- ◯ Yes, with suggestions

79. What is the strength of this recommendation ?

- ◯ it must be applied widely and to all patients with this specific need
- ◯ it is important, but can be applied not to all patients with this specific need
- ◯ less important, it can be applied on a voluntary basis
- ◯ very low importance

2016 recommendations - third round

Physiotherapeutic Scoliosis Specific Exercises

It is recommented that Physiotherapeutic Scoliosis Specific Exercises difficulty is progressively increased according to patient ability

80. Do you agree with this recommendation ?

- No
- Yes
- Yes, with suggestions

81. What is the strength of this recommendation ?

- it must be applied widely and to all patients with this specific need
- it is important, but can be applied not to all patients with this specific need
- less important, it can be applied on a voluntary basis
- very low importance

2016 recommendations - third round

Physiotherapeutic Scoliosis Specific Exercises

It is recommended that Physiotherapeutic Scoliosis Specific Exercises are taught individually in a 1 to 1 relationship to assure individualized care, while regular performance could also be at home or in little groups

82. Do you agree with this recommendation ?

- ◯ No
- ◯ Yes
- ◯ Yes, with suggestions

83. What is the strength of this recommendation ?

- ◯ it must be applied widely and to all patients with this specific need
- ◯ it is important, but can be applied not to all patients with this specific need
- ◯ less important, it can be applied on a voluntary basis
- ◯ very low importance

2016 recommendations - third round

Physiotherapeutic Scoliosis Specific Exercises during brace treatment and surgical therapy

It is recommended that Physiotherapeutic Scoliosis Specific Exercises are performed during brace treatment
LoE: II

84. Do you agree with this recommendation ?

- ◯ No
- ◯ Yes
- ◯ Yes, with suggestions

85. What is the strength of this recommendation ?

- ◯ it must be applied widely and to all patients with this specific need
- ◯ it is important, but can be applied not to all patients with this specific need
- ◯ less important, it can be applied on a voluntary basis
- ◯ very low importance

2016 recommendations - third round

Physiotherapeutic Scoliosis Specific Exercises during brace treatment and surgical therapy

It is recommended that, while treating with Physiotherapeutic Scoliosis Specific Exercises, therapists strongly encourage patients to comply with the brace treatment
LoE: II

86. Do you agree with this recommendation ?

- ○ No
- ○ Yes
- ○ Yes, with suggestions

87. What is the strength of this recommendation ?

- ○ it must be applied widely and to all patients with this specific need
- ○ it is important, but can be applied not to all patients with this specific need
- ○ less important, it can be applied on a voluntary basis
- ○ very low importance

2016 recommendations - third round

Physiotherapeutic Scoliosis Specific Exercises during brace treatment and surgical therapy

It is recommended that spinal mobilization Physiotherapeutic Scoliosis Specific Exercises are used in preparation to bracing

88. Do you agree with this recommendation ?

- ◯ No
- ◯ Yes
- ◯ Yes, with the suggestions

89. What is the strength of this recommendation ?

- ◯ it must be applied widely and to all patients with this specific need
- ◯ it is important, but can be applied not to all patients with this specific need
- ◯ less important, it can be applied on a voluntary basis
- ◯ very low importance

2016 recommendations - third round

Physiotherapeutic Scoliosis Specific Exercises during brace treatment and surgical therapy

It is recommended that stabilization Physiotherapeutic Scoliosis Specific Exercises in autocorrection are used during brace weaning period

90. Do you agree with this recommendation ?

- No
- Yes
- Yes, with the suggestions

91. What is the strength of this recommendation ?

- it must be applied widely and to all patients with this specific need
- it is important, but can be applied not to all patients with this specific need
- less important, it can be applied on a voluntary basis
- very low importance

2016 recommendations - third round

Physiotherapeutic Scoliosis Specific Exercises during brace treatment and surgical therapy

It is recommended that Physiotherapeutic Scoliosis Specific Exercises (eventually with additional interventions) are used in operated patients to reduce pain, increase function and posture, optimize protection of unfused segments, and improve respiratory function

92. Do you agree with this recommendation ?

- ◯ No
- ◯ Yes
- ◯ Yes, with suggestions

93. What is the strength of this recommendation ?

- ◯ it must be applied widely and to all patients with this specific need
- ◯ it is important, but can be applied not to all patients with this specific need
- ◯ less important, it can be applied on a voluntary basis
- ◯ very low importance

2016 recommendations - third round

Physiotherapeutic Scoliosis Specific Exercises during brace treatment and surgical therapy

It is recommended that aerobic therapy training is used prior to surgery.
LoE: II

94. Do you agree with this recommendation ?

- No
- Yes
- Yes, with suggestions

95. What is the strength of this recommendation ?

- it must be applied widely and to all patients with this specific need
- it is important, but can be applied not to all patients with this specific need
- less important, it can be applied on a voluntary basis
- very low importance

2016 recommendations - third round

Other conservative treatments

It is recommended that manual therapy (gentle, short-term mobilization, or releasing soft tissues techniques) is proposed only if associated with stabilization Physiotherapeutic Scoliosis Specific Exercises, unless otherwise justified in the opinion of a clinician specialized in conservative treatment of spinal deformities

96. Do you agree with this recommendation ?

- ◯ No
- ◯ Yes
- ◯ Yes, with suggestions

97. What is the strength of this recommendation ?

- ◯ it must be applied widely and to all patients with this specific need
- ◯ it is important, but can be applied not to all patients with this specific need
- ◯ less important, it can be applied on a voluntary basis
- ◯ very low importance

2016 recommendations - third round

Other conservative treatments

It is recommended that correction of real leg length discrepancy, if needed, is prescribed by a clinician specialized in conservative treatment of spinal deformities

98. Do you agree with this recommendation ?

- No
- Yes
- Yes, with suggestions

99. What is the strength of this recommendation ?

- it must be applied widely and to all patients with this specific need
- it is important, but can be applied not to all patients with this specific need
- less important, it can be applied on a voluntary basis
- very low importance

2016 recommendations - third round

Respiratory function and exercises

It is recommended that, when needed, exercises to improve respiratory function are used

100. Do you agree with this recommendation ?

- No
- Yes
- Yes, with suggestions

101. What is the strength of this recommendation ?

- it must be applied widely and to all patients with this specific need
- it is important, but can be applied not to all patients with this specific need
- less important, it can be applied on a voluntary basis
- very low importance

2016 recommendations - third round

Respiratory function and exercises

It is recommended during brace treatment to use exercises to improve respiratory function

102. Do you agree with this recommendation ?

- No
- Yes
- Yes, with suggestions

103. What is the strength of this recommendation ?

- it must be applied widely and to all patients with this specific need
- it is important, but can be applied not to all patients with this specific need
- less important, it can be applied on a voluntary basis
- very low importance

2016 recommendations - third round

Respiratory function and exercises

It is recommended the use of Physiotherapeutic Scoliosis Specific Exercises to improve respiratory function and train regional respiratory strategies to promote the expansion and ventilation of specific lung compartments

104. Do you agree with this recommendation ?

- No
- Yes
- Yes, with suggestions

105. What is the strength of this recommendation ?

- it must be applied widely and to all patients with this specific need
- it is important, but can be applied not to all patients with this specific need
- less important, it can be applied on a voluntary basis
- very low importance

2016 recommendations - third round

Sport activities

It is recommended that sports is not prescribed as a treatment for idiopathic scoliosis
LoE: III

106. Do you agree with this recommendation ?

- ○ No
- ○ Yes
- ○ Yes, with suggestions

107. What is the strength of this recommendation ?

- ○ it must be applied widely and to all patients with this specific need
- ○ it is important, but can be applied not to all patients with this specific need
- ○ less important, it can be applied on a voluntary basis
- ○ very low importance

2016 recommendations - third round

Sport activities

It is recommended that general sports activities are performed because of the benefits they offer to patients in terms of psychological, neuromotor and general organic well-being

108. Do you agree with this recommendation ?

- No
- Yes
- Yes, with suggestions

109. What is the strength of this recommendation ?

- it must be applied widely and to all patients with this specific need
- it is important, but can be applied not to all patients with this specific need
- less important, it can be applied on a voluntary basis
- very low importance

2016 recommendations - third round

Sport activities

It is recommended that, during all treatment phases, physical education at school is continued

110. Do you agree with this recommendation ?

- No
- Yes
- Yes, with suggestions

111. What is the strength of this recommendation ?

- it must be applied widely and to all patients with this specific need
- it is important, but can be applied not to all patients with this specific need
- less important, it can be applied on a voluntary basis
- very low importance

2016 recommendations - third round

Sport activities

It is recommended that sports activities are continued also during brace treatment because of the physical and psychological benefits these activities provide

112. Do you agree with this recommendation ?

- No
- Yes
- Yes, with suggestions

113. What is the strength of this recommendation ?

- it must be applied widely and to all patients with this specific need
- it is important, but can be applied not to all patients with this specific need
- less important, it can be applied on a voluntary basis
- very low importance

2016 recommendations - third round

Sport activities

It is recommended that, during brace treatment, contact or highly dynamic sport activities are performed with caution

114. Do you agree with this recommendation ?

- ◯ No
- ◯ Yes
- ◯ Yes, with suggestions

115. What is the strength of this recommendation ?

- ◯ it must be applied widely and to all patients with this specific need
- ◯ it is important, but can be applied not to all patients with this specific need
- ◯ less important, it can be applied on a voluntary basis
- ◯ very low importance

2016 recommendations - third round

Sport activities

It is recommended that competitive activities that greatly mobilize the spine are avoided in patients with scoliosis at high risk of progression
LoE: III

116. Do you agree with this recommendation ?

- No
- Yes
- Yes, with suggestions

117. What is the strength of this recommendation ?

- it must be applied widely and to all patients with this specific need
- it is important, but can be applied not to all patients with this specific need
- less important, it can be applied on a voluntary basis
- very low importance

2016 recommendations - third round

Assessment

It is recommended to use school screening programs for the early diagnosis of idiopathic scoliosis

118. Do you agree with this recommendation ?

- No
- Yes
- Yes, with suggestions

119. What is the strength of this recommendation ?

- it must be applied widely and to all patients with this specific need
- it is important, but can be applied not to all patients with this specific need
- less important, it can be applied on a voluntary basis
- very low importance

2016 recommendations - third round

Assessment

It is recommended to use the Scoliometer (or other inclinometers) in forward bending (Adam`s test) to perform school screening programs

120. Do you agree with this recommendation ?

- ◯ No
- ◯ Yes
- ◯ Yes, with suggestions

121. What is the strength of this recommendation ?

- ◯ it must be applied widely and to all patients with this specific need
- ◯ it is important, but can be applied not to all patients with this specific need
- ◯ less important, it can be applied on a voluntary basis
- ◯ very low importance

2016 recommendations - third round

Assessment

It is recommended that for scoliosis screening programs 7° of angle of trunk rotation should be used as criteria for referral, and 5° for controls in time

122. Do you agree that 7° is good cut-off for referral and 5° to control again the patient in future screening activities ?

- ◯ No, leave only the 7° cut-off
- ◯ Yes, leave both cut-offs

123. Do you agree with this recommendation ?

- ◯ No
- ◯ Yes
- ◯ Yes, with suggestions

124. What is the strength of this recommendation ?

- ◯ it must be applied widely and to all patients with this specific need
- ◯ it is important, but can be applied not to all patients with this specific need
- ◯ less important, it can be applied on a voluntary basis
- ◯ very low importance

2016 recommendations - third round

Assessment

It is recommended that the angle of trunk rotation with using of scoliometer or smartphone aided application is promoted in the school community and among pediatricians, sports physicians and all people that are engaged in the health of children (parents included)

125. Do you agree with this recommendation ?

- No
- Yes
- Yes, with suggestions

126. What is the strength of this recommendation ?

- it must be applied widely and to all patients with this specific need
- it is important, but can be applied not to all patients with this specific need
- less important, it can be applied on a voluntary basis
- very low importance

2016 recommendations - third round

Assessment

It is recommended that patients are always examined by the same clinicians specialized in spinal deformities. In settings in which this is not possible, it is recommended regular standardization and validation processes of the methods used

127. Do you agree with this recommendation ?

- ◯ No
- ◯ Yes
- ◯ Yes, with suggestions

128. What is the strength of this recommendation ?

- ◯ it must be applied widely and to all patients with this specific need
- ◯ it is important, but can be applied not to all patients with this specific need
- ◯ less important, it can be applied on a voluntary basis
- ◯ very low importance

2016 recommendations - third round

Assessment

It is recommended not to perform x-rays if the Adam's test is negative and the Scoliometer value is below 5°, unless otherwise justified in the opinion of a clinician specialized in conservative treatment of spinal deformities

129. Do you agree with this recommendation ?

- ◯ No
- ◯ Yes
- ◯ Yes, with suggestions

130. What is the strength of this recommendation ?

- ◯ it must be applied widely and to all patients with this specific need
- ◯ it is important, but can be applied not to all patients with this specific need
- ◯ less important, it can be applied on a voluntary basis
- ◯ very low importance

2016 recommendations - third round

Assessment

It is recommended that, every time they evaluate children aged from 8 to 15 years, pediatricians, general practitioners and sports physicians perform the Adam’s test for scoliosis screening purposes, using the Scoliometer or another inclinometer

131. Do you agree with this recommendation ?

- No
- Yes
- Yes, with suggestions

132. What is the strength of this recommendation ?

- it must be applied widely and to all patients with this specific need
- it is important, but can be applied not to all patients with this specific need
- less important, it can be applied on a voluntary basis
- very low importance

2016 recommendations - third round

Assessment

It is recommended for clinical follow-up the use of validated methods of assessment and standard clinical data collection forms

133. Do you agree with this recommendation ?

- ◯ No
- ◯ Yes
- ◯ Yes, with suggestions

134. What is the strength of this recommendation ?

- ◯ it must be applied widely and to all patients with this specific need
- ◯ it is important, but can be applied not to all patients with this specific need
- ◯ less important, it can be applied on a voluntary basis
- ◯ very low importance

2016 recommendations - third round

Assessment

It is recommended to take into account the measurement error appointed for each method applied for the assessment of scoliosis patients

135. Do you agree with this recommendation ?

- ◯ No
- ◯ Yes
- ◯ Yes, with suggestions

136. What is the strength of this recommendation ?

- ◯ it must be applied widely and to all patients with this specific need
- ◯ it is important, but can be applied not to all patients with this specific need
- ◯ less important, it can be applied on a voluntary basis
- ◯ very low importance

2016 recommendations - third round

Assessment

It is recommended to clinically assess in scoliosis patients at least: angle of trunk rotation, aesthetics, and sagittal alignment of the spine. Other possible common evaluations include: pain, respiratory function, spine and joint flexibility and strength, leg length discrepancy, balance and coordination, quality of life

137. Do you agree with this recommendation ?

- ○ No
- ○ Yes
- ○ Yes, with suggestions

138. What is the strength of this recommendation ?

- ○ it must be applied widely and to all patients with this specific need
- ○ it is important, but can be applied not to all patients with this specific need
- ○ less important, it can be applied on a voluntary basis
- ○ very low importance

2016 recommendations - third round

Assessment

It is recommended that aesthetics is assessed with validated methods, objectively (clinically with TRACE, and/or instrumentally with surface topography or photography) and subjectively (questionnaires)

139. Do you agree with this recommendation ?

- No
- Yes
- Yes, with suggestions

140. What is the strength of this recommendation ?

- it must be applied widely and to all patients with this specific need
- it is important, but can be applied not to all patients with this specific need
- less important, it can be applied on a voluntary basis
- very low importance

2016 recommendations - third round

Assessment

It is recommended to asses the sagittal spinal balance with lateral X-ray

141. Do you agree with this recommendation ?

- No
- Yes
- Yes, with suggestions

142. What is the strength of this recommendation ?

- it must be applied widely and to all patients with this specific need
- it is important, but can be applied not to all patients with this specific need
- less important, it can be applied on a voluntary basis
- very low importance

2016 recommendations - third round

Assessment

It is recommended to objectively measure and follow up clinically the sagittal plane of patients (using inclinometers and/or plumb line distance)

143. Do you agree with this recommendation ?

- No
- Yes
- Yes, with suggestions

144. What is the strength of this recommendation ?

- it must be applied widely and to all patients with this specific need
- it is important, but can be applied not to all patients with this specific need
- less important, it can be applied on a voluntary basis
- very low importance

2016 recommendations - third round

Assessment

It is recommended to regularly perform quality of life assessment through validated questionnaires

145. Do you agree with this recommendation ?

- No
- Yes
- Yes, with suggestions

146. What is the strength of this recommendation ?

- it must be applied widely and to all patients with this specific need
- it is important, but can be applied not to all patients with this specific need
- less important, it can be applied on a voluntary basis
- very low importance

2016 recommendations - third round

Assessment

It is recommended to assess and measure joint hypermobility/laxity in children qualified to conservative treatment (especially Physiotherapeutic Scoliosis Specific Exercises)

147. Do you agree with this recommendation ?

- No
- Yes
- Yes, with suggestions

148. What is the strength of this recommendation ?

- it must be applied widely and to all patients with this specific need
- it is important, but can be applied not to all patients with this specific need
- less important, it can be applied on a voluntary basis
- very low importance

2016 recommendations - third round

Assessment

It is recommended to assess function of respiratory system and physical capacity in scoliosis patients
LoE: III

149. Do you agree with this recommendation ?

- No
- Yes
- Yes, with suggestions

150. What is the strength of this recommendation ?

- it must be applied widely and to all patients with this specific need
- it is important, but can be applied not to all patients with this specific need
- less important, it can be applied on a voluntary basis
- very low importance

2016 recommendations - third round

Assessment

It is recommended during growth that clinical follow-up examinations are performed twice per year, or more often during periods of rapid growth (pubertal spurt, first three years of life)

151. Do you agree with this recommendation ?

- No
- Yes
- Yes, with suggestions

152. What is the strength of this recommendation ?

- it must be applied widely and to all patients with this specific need
- it is important, but can be applied not to all patients with this specific need
- less important, it can be applied on a voluntary basis
- very low importance

2016 recommendations - third round

Assessment

It is recommended that the decision whether to perform a radiographic study should be made by a physician specialized in spinal deformities

153. Do you agree with this recommendation ?

- No
- Yes
- Yes, with suggestions

154. What is the strength of this recommendation ?

- it must be applied widely and to all patients with this specific need
- it is important, but can be applied not to all patients with this specific need
- less important, it can be applied on a voluntary basis
- very low importance

2016 recommendations - third round

Assessment

It is recommended that frontal radiographic studies are made postero-anteriorly, including visualization of the femoral heads and protection of the gonads, in standing position without the use of support aids or indication of correct posture, unless otherwise justified in the opinion of a clinician specialized in spinal deformities

155. Do you agree with this recommendation ?

- No
- Yes
- Yes, with suggestions

156. What is the strength of this recommendation ?

- it must be applied widely and to all patients with this specific need
- it is important, but can be applied not to all patients with this specific need
- less important, it can be applied on a voluntary basis
- very low importance

2016 recommendations - third round

Assessment

It is recommended that curve magnitude is measured using the Cobb method

157. Do you agree with this recommendation ?

- No
- Yes
- Yes, with suggestions

158. What is the strength of this recommendation ?

- it must be applied widely and to all patients with this specific need
- it is important, but can be applied not to all patients with this specific need
- less important, it can be applied on a voluntary basis
- very low importance

2016 recommendations - third round

Assessment

It is recommended that vertebral rotation is measured on the apical vertebra using either the Perdriolle torsiometer or the Raimondi tables/ruler

159. Do you agree with this recommendation ?

- ◯ No
- ◯ Yes
- ◯ Yes, with suggestions

160. What is the strength of this recommendation ?

- ◯ it must be applied widely and to all patients with this specific need
- ◯ it is important, but can be applied not to all patients with this specific need
- ◯ less important, it can be applied on a voluntary basis
- ◯ very low importance

2016 recommendations - third round

Assessment

It is recommended that the first and last radiographic evaluation include also a standing lateral view

161. Do you agree with this recommendation ?

- ◯ No
- ◯ Yes
- ◯ Yes, with suggestions

162. What is the strength of this recommendation ?

- ◯ it must be applied widely and to all patients with this specific need
- ◯ it is important, but can be applied not to all patients with this specific need
- ◯ less important, it can be applied on a voluntary basis
- ◯ very low importance

2016 recommendations - third round

Assessment

It is recommended that, on radiographic lateral view, the patient's upper extremities be placed in a position to uncover the upper thoracic spine. They comprise: (1) 45° angle flexion of the arms, elbows extended and hands resting on a support to preserve the sagittal curvature of the spine, (2) the arms crossed over the breasts , (3) the hand resting on the ipsilateral shoulder without pressing it, (4) the hand resting on the zygomi without pressing them

163. Do you agree with this recommendation ?

- No
- Yes
- Yes, with suggestions

164. What is the strength of this recommendation ?

- it must be applied widely and to all patients with this specific need
- it is important, but can be applied not to all patients with this specific need
- less important, it can be applied on a voluntary basis
- very low importance

2016 recommendations - third round

Assessment

It is recommended, to reduce the invasiness, to use whenever possible a ultra-low dose x-rays technique (EOS). No more than 1 radiographic study every 5-6 cm of growth should be performed, unless it is truly necessary and is decided by a clinician specialized in spinal diseases

165. Do you agree with this recommendation ?

- No
- Yes
- Yes, with suggestions

166. What is the strength of this recommendation ?

- it must be applied widely and to all patients with this specific need
- it is important, but can be applied not to all patients with this specific need
- less important, it can be applied on a voluntary basis
- very low importance

2016 recommendations - third round

Assessment

It is recommended that the least number of projections is made on radiographic studies to reduce the invasiveness of follow-ups

167. Do you agree with this recommendation ?

- No
- Yes
- Yes, with suggestions

168. What is the strength of this recommendation ?

- it must be applied widely and to all patients with this specific need
- it is important, but can be applied not to all patients with this specific need
- less important, it can be applied on a voluntary basis
- very low importance

2016 recommendations - third round

Assessment

It is recommended that all idiopathic scoliosis patients, even if not treated, are regularly followed-up

169. Do you agree with this recommendation ?

- No
- Yes
- Yes, with suggestions

170. What is the strength of this recommendation ?

- it must be applied widely and to all patients with this specific need
- it is important, but can be applied not to all patients with this specific need
- less important, it can be applied on a voluntary basis
- very low importance
